# ITGBL1-MYH9 interaction in hepatic stellate cells acts as a mechanoregulator controlling liver fibrosis in mice

**DOI:** 10.1172/JCI203412

**Published:** 2026-07-16

**Authors:** Yixin Li, Yan Wang, Chenhao Tong, Xinghuan Fu, Ningning Ma, Yawen Hao, Zian Feng, Shijia Ling, Zequn Yin, Haodong Li, Shujun Ge, Siting Yang, Peng Xiao, Siyue Dong, Adrien Guillot, Yajun Duan, Yong He

**Affiliations:** 1 Department of Cardiology, The First Affiliated Hospital of USTC, Division of Life Sciences and Medicine, University of Science and Technology of China, Hefei, China.; 2 State Key Laboratory of Drug Research, Shanghai Institute of Materia Medica (SIMM), Chinese Academy of Sciences, Shanghai, China.; 3 Department of Infectious Diseases, Peking University First Hospital, Beijing, China.; 4 Hepatopancreatobiliary Surgery Department, the First Affiliated Hospital of Ningbo University, Ningbo, Zhejiang, China.; 5 University of Chinese Academy of Sciences, Beijing, China.; 6 School of Chinese Materia Medica, Nanjing University of Chinese Medicine, Nanjing, China.; 7 Tianjing University of Traditional Chinese Medicine, Tianjin, China.; 8 Department of Hepatology, the First Hospital of Jilin University, Changchun, China.; 9 Department of Hepatology & Gastroenterology, Charité-Universitätsmedizin Berlin, Campus Virchow-Klinikum and Campus Charité Mitte, Berlin, Germany.

**Keywords:** Cell biology, Hepatology, Fibrosis, Molecular biology, Molecular pathology

## Abstract

Hepatic stellate cell (HSC) activation can lead to liver fibrosis, for which there are no effective treatments. Aberrant cytoskeletal reorganization is a central driver of HSC activation. Non-muscle myosin II (NM II) is known to regulate cytoskeleton remodeling via its actin cross-linking and contractile properties. However, the molecular players controlling actomyosin assembly and contractility in HSCs during liver fibrosis remain poorly defined. Here, we identified integrin β-like 1 (ITGBL1) as a gatekeeper of HSC quiescence by negatively regulating actomyosin contractility-driven mechanotransduction in HSCs. ITGBL1 expression was markedly elevated in activated HSCs found in patient and mouse fibrotic livers. Unexpectedly, HSC-specific *Itgbl1* deficiency worsened liver fibrosis, whereas ITGBL1 overexpression in HSCs limited it, suggesting a protective role for ITGBL1 against a pathogenic HSC activation. Multiomics and functional analyses revealed that ITGBL1 impaired F-actin filament organization in HSCs by disrupting actomyosin assembly dependent on myosin heavy chain 9 (MYH9, also named NM II heavy chain A). In line, HSC-specific *Myh9* deficiency or silencing of *Myh9* in HSCs alleviated liver fibrosis. Taken together, our findings unveil that ITGBL1-MYH9 interaction acts as a critical mechanoregulatory brake that maintains cytoskeletal equilibrium and mechanical homeostasis in HSCs, providing a promising therapeutic strategy to combat liver fibrosis.

## Abstract 2

ITGBL1-MYH9 interaction in HSCs acts as a critical mechano-regulatory brake that limits liver fibrogenesis in mice.

## Graphical abstract

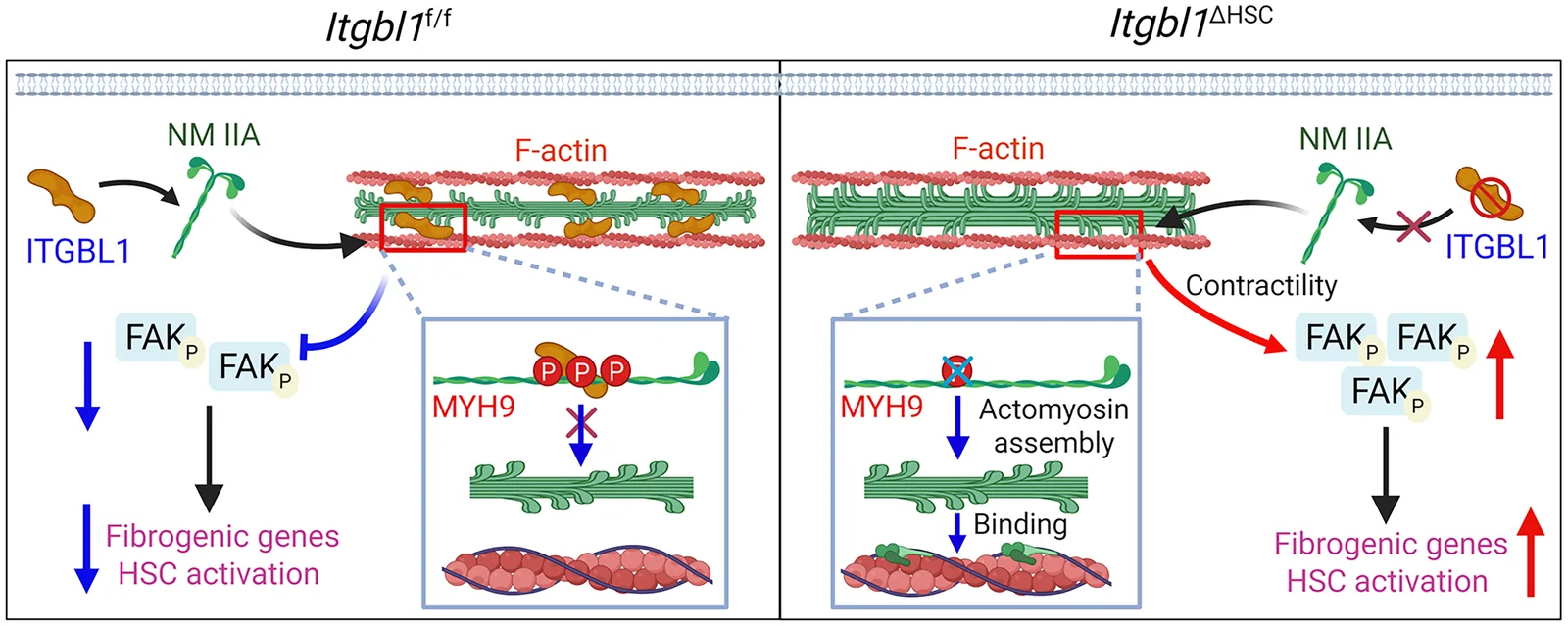

## Introduction

Chronic liver injury, from diverse etiologies including viral hepatitis, alcohol abuse, and metabolic dysfunction, is characterized by hepatocyte damage, inflammation, and an excessive wound healing response marked by progressive fibrosis (1). Accumulating evidence suggests that unresolved fibrosis disrupts liver architecture, ultimately leading to cirrhosis and hepatocellular carcinoma, 2 main causes of liver-related mortality worldwide (2). The key event in this process is hepatic stellate cell (HSC) activation, during which HSCs transdifferentiate from quiescent, vitamin A–storing cells into proliferative, contractile, myofibroblast-like cells that secrete extracellular matrix (ECM) proteins, especially collagen types I and III (3, 4). Despite the clinical burden, there is no FDA-approved therapy for the treatment of liver fibrosis, which emphasizes the urgent need to clarify the molecular mechanism driving HSC activation and fibrogenesis.

Cytoskeleton, a complex interconnected protein filamentous meshwork, consists of intermediate filaments, microtubules, and microfilaments, which provide structural support and mechanical strength and maintain cell polarity (5). The main cytoskeletal protein of most cells is actin, which polymerizes to form actin filaments, playing a critical role in cellular architecture, particularly at the cell periphery and cortex (6). Myosins constitute a superfamily of motor proteins that are involved in several cellular processes that require force and translocation, including cell adhesion, cell migration, and cell activation, by converting chemical energy into translational or rotational movement (7). Most myosins belong to class II, named non-muscle myosin II (NM II), which generates most mechanical force in eukaryotic cells. Mammalian cells have 3 isoforms of NM II, termed NM IIA, NM IIB, and NM IIC, encoded by 3 genes including myosin heavy chain 9 (*MYH9*), *MYH10*, and *MYH14* (8). Despite considerable homology, NM II isoforms exhibit differences in kinetic properties, subcellular localization, and tissue expression patterns (9). NM II has a fundamental role in processes that require cellular reshaping and movement due to its actin cross-linking and contractile properties (10). Actomyosin, referring to the actin-myosin complex, is inherently contractile, with the myosin motor protein able to pull on actin filaments. NM II–mediated actomyosin contractility is a highly conserved mechanism for generating mechanical stress in animal cells and underlies cell migration, cell division, and tissue morphogenesis (11). Recent studies have highlighted the importance of mechanical cues in fibrotic diseases (12, 13). A key consequence of mechanosensing during liver fibrosis is cytoskeletal remodeling in HSCs: activated HSCs reorganize their actin cytoskeleton and increase actomyosin contractility, thus activating profibrotic signaling pathways such as focal adhesion kinase (FAK) signaling, YAP/TAZ signaling, and RhoA/ROCK signaling (14, 15). However, the regulatory factors controlling actomyosin assembly and contractility in HSCs that affect liver fibrosis progression remain poorly understood.

Integrin β-like 1 (ITGBL1) is a structurally unique, β-integrin–related protein characterized by 10 EGF-like repeats but lacks both a transmembrane domain and a classical Arg-Gly-Asp–binding (RGD-binding) motif (16). Although originally indicated in chondrogenesis and tumor progression (17, 18), its functional role in the development of liver fibrosis remains unknown. In this study, we identified ITGBL1 as a critical negative regulator of actomyosin contractility-driven mechanotransduction during HSC activation. First, ITGBL1 was markedly elevated in activated HSCs during both choline-deficient, l-amino acid–defined high-fat diet– (CDAHFD) and CCl_4_-induced experimental liver fibrosis. Strikingly, HSC-specific deletion of *Itgbl1* exacerbated experimental mouse liver fibrosis, whereas overexpression of ITGBL1 restrained HSC activation by disrupting actin cytoskeleton reorganization. Multitranscriptomic and proteomics analysis revealed that ITGBL1 preferentially disrupted F-actin filament organization in HSCs by impairing actomyosin assembly mediated by MYH9 (also named NM IIA heavy chain, NMHC IIA), thus limiting HSC activation. Notably, HSC-specific *Myh9* deficiency or silencing of *Myh9* in HSCs mitigated liver fibrosis, reinforcing the key functional importance of ITGBL1-MYH9 interaction during HSC activation. In conclusion, our findings uncover that the ITGBL1-MYH9 interaction acts as a key mechanoregulator that governs cytoskeletal dynamics and mechanical homeostasis in HSCs, presenting a promising therapeutic direction for the treatment of liver fibrosis.

## Results

### ITGBL1 is mainly distributed in HSCs,and its deficiency in HSCs worsens liver fibrosis.

**Figure 1:**
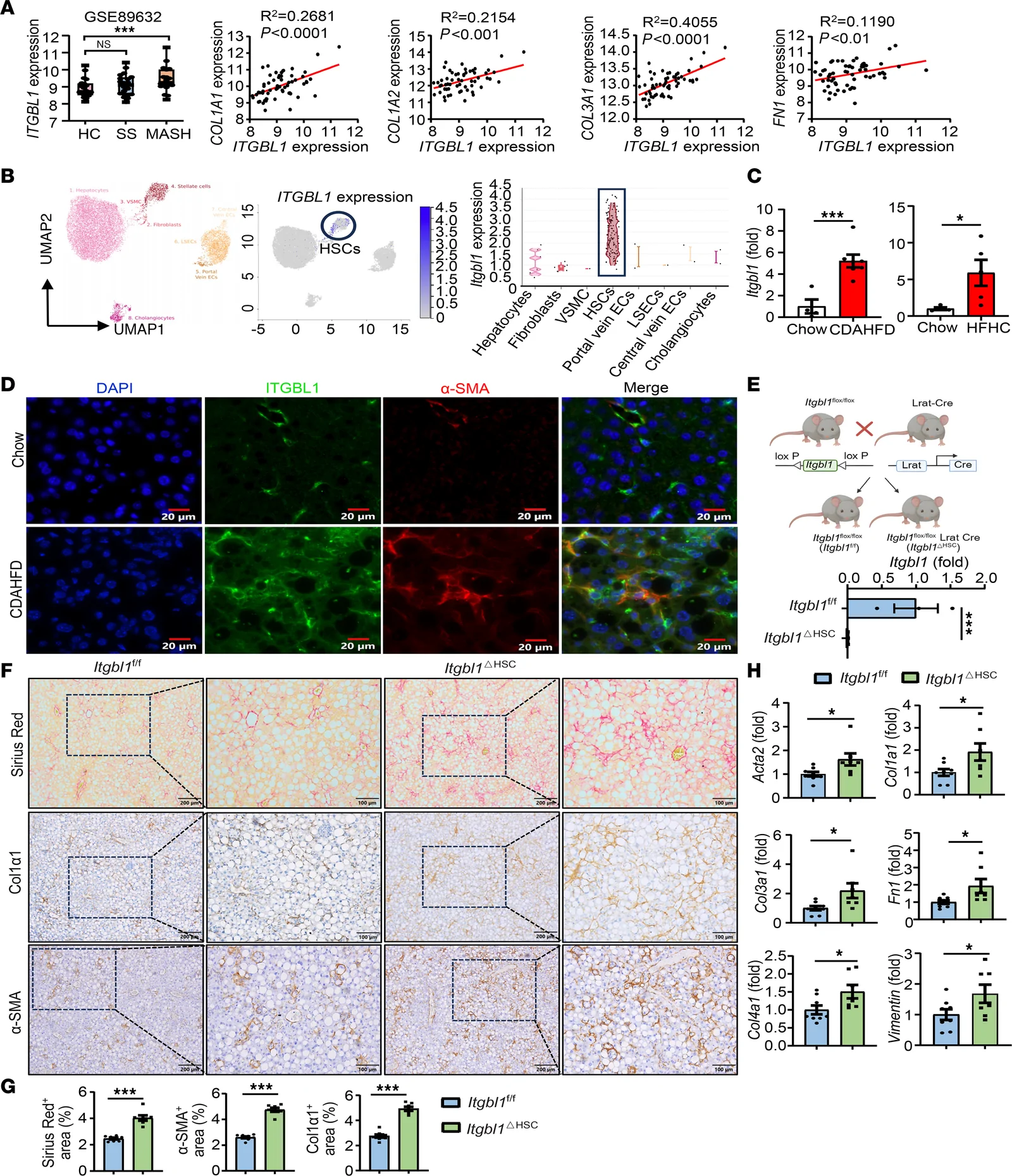
Loss of *Itgbl1* in HSCs aggravates MASH-related liver fibrosis. (**A**) The hepatic expression of *ITGBL1* in the livers of patients with SS and MASH was analyzed in a public dataset (NCBI GEO GSE89632). The correlation between *ITGBL1* and liver fibrogenic genes including *COL1A1*, *COL1A2*, *COL3A1*, and *FN1* was analyzed. (**B**) Hepatic *ITGBL1* expression was analyzed by scRNA-seq from Liver Cell Atlas database (https://www.livercellatlas.org). UMAP, uniform manifold approximation and projection; VSMC, vascular smooth muscle cells; LSEC, liver sinusoidal endothelial cells. (**C**) RT-qPCR analysis of hepatic *Itgbl1* mRNA levels in CDAHFD- and HFHC-induced liver fibrosis. (**D**) Representative images of immunofluorescence double-staining of ITGBL1 (green) and α-SMA (red) in CDAHFD-fed mice are shown. (**E**) *Itgbl1*-floxed mice were crossed with Lrat Cre mice for several generations to generate *Itgbl1*^ΔHSC^ mice. The knockout efficiency was verified by RT-qPCR analysis by isolating primary mouse HSCs from *Itgbl1*^fl/fl^ and *Itgbl1*^ΔHSC^ mice. (**F**) Representative images of Sirius red staining and IHC staining of Col1α1 and α-SMA are shown. (**G**) Quantification of Sirius red–, Col1α1-, and α-SMA–positive area. (**H**) The fibrotic genes were detected by RT-qPCR analysis. Values represent means ± SEM. Significance was calculated using a 2-tailed Student’s *t* test (**A**, **C**, **G**, and **H**). *P < 0.05, ***P < 0.001.

To investigate whether ITGBL1 may be associated with the progression of MASH-related liver fibrosis, we first analyzed its gene expression in liver tissues from the National Center for Biotechnology Information (NCBI) Gene Expression Omnibus (GEO) public database, including patients with simple steatosis (SS) and metabolic dysfunction–associated steatohepatitis (MASH). As shown in Figure 1A and Supplemental Figure 1A; supplemental material available online with this article; https://doi.org/10.1172/JCI203412DS1, hepatic *ITGBL1* mRNA levels were significantly upregulated in patients with MASH, but not in patients with SS, and positively correlated with the expression of several liver fibrogenic genes including *COL1A1*, *COL2A1*, *COL3A1*, and *FN1*. Furthermore, single-cell RNA-sequencing (scRNA-seq) analysis from Liver Cell Atlas database (https://www.livercellatlas.org) revealed that hepatic *ITGBL1* expression in human liver was restricted to HSCs (Figure 1B). Moreover, RT-qPCR and immunohistochemical staining demonstrated that mRNA and protein levels of ITGBL1 were markedly upregulated in experimental mouse models of MASH-related liver fibrosis induced by CDAHFD and high-fat, high-cholesterol (HFHC) diet feeding (Figure 1C and Supplemental Figure 1B). Immunofluorescence double-staining confirmed colocalization of ITGBL1 with α–smooth muscle actin (α-SMA), a marker for activated HSCs, in the livers of mice subjected to CDAHFD and HFHC feeding (Figure 1D and Supplemental Figure 1C). Collectively, these findings indicate that ITGBL1 may be involved in the development of MASH.

To further elucidate the functional role of ITGBL1 in MASH-associated liver fibrosis, we generated HSC-specific *Itgbl1*-knockout mice (*Itgbl1*^ΔHSC^) and validated the knockout efficiency in HSCs (Figure 1E). Unexpectedly, after CDAHFD feeding for 4 weeks, *Itgbl1*^ΔHSC^ mice exhibited greater degree of liver fibrosis compared with control mice (*Itgbl1*^fl/fl^), as evidenced by increased collagen deposition and higher α-SMA–positive areas in *Itgbl1*^ΔHSC^ mice (Figure 1, F and G). These observations were further confirmed by RT-qPCR and Western blot analyses (Figure 1H and Supplemental Figure 2A). In addition, there was an increased accumulation of F4/80-positive macrophages in the liver tissues of *Itgbl1*^ΔHSC^ mice, whereas no significant difference in liver steatosis and neutrophil infiltration was observed between *Itgbl1*^fl/fl^ and *Itgbl1*^ΔHSC^ mice (Supplemental Figure 2, B and C). Notably, immunohistochemical analysis of E-cadherin and glutamine synthetase demonstrated that liver zonation and liver architectural organization were not altered by specific deletion of *Itgbl1* in HSCs after CDAHFD feeding (Supplemental Figure 2D). These results suggest that ITGBL1 plays a protective role in the progression of MASH-related liver fibrosis.

Since CDAHFD-induced MASH-related liver fibrosis is mainly caused by metabolic dysfunction–associated factors (19), we wondered whether ITGBL1 was involved in the development of chronic liver injury–driven liver fibrosis. To answer this question, we first analyzed the expression of ITGBL1 in liver fibrosis caused by hepatitis B virus (HBV). As shown in Supplemental Figure 3A, hepatic *ITGBL1* mRNA levels were markedly increased in patients with HBV infection and showed a correlation with the expression of fibrosis-related genes, such as *ACTA2* and *COL3A1*. Meanwhile, the expression of *Itgbl1* was also significantly elevated in the livers of fibrotic mice induced by chronic CCl_4_ injection (Supplemental Figure 3, B and C). Single-cell transcriptomics analysis from a public database revealed a significant upregulation of *Itgbl1* expression in both a CCl_4_-induced liver fibrosis mouse model and HSCs from patients with liver cirrhosis (Supplemental Figure 3D). Next, we performed immunofluorescence staining on liver tissues from CCl_4_-induced fibrosis mice and observed the colocalization of ITGBL1 with the HSC marker desmin (Supplemental Figure 3E).

Next, *Itgbl1*^fl/fl^ and *Itgbl1*^ΔHSC^ mice were subjected to CCl_4_-induced liver fibrosis (Supplemental Figure 3F). Strikingly, immunohistochemical staining analysis demonstrated that *Itgbl1* deficiency in HSCs resulted in a marked increase in Sirius red–, α-SMA–, and Col1α1-positive areas following chronic CCl_4_ injection, indicating enhanced HSC activation and accelerated fibrosis progression in *Itgbl1*^ΔHSC^ mice compared with *Itgbl1*^fl/fl^ mice (Supplemental Figure 3G). Consistently, loss of *Itgbl1* in HSCs markedly elevated the levels of liver fibrosis–related proteins, including α-SMA, Col1α1, and Col3α1, as demonstrated by Western blotting (Supplemental Figure 3H). In contrast, *Itgbl1* deletion in HSCs did not markedly affect hepatic inflammatory responses in the CCl_4_ model, as shown by comparable inflammatory marker expression between groups (Supplemental Figure 4, A and B), nor did it alter spleen weight (Supplemental Figure 4C), suggesting that ITGBL1 in HSCs did not influence systemic inflammatory response and portal hypertension–associated changes during liver fibrosis development. Furthermore, to define whether deletion of *Itgbl1* in HSCs will lead to spontaneous liver fibrosis in mice, *Itgbl1*^fl/fl^ and *Itgbl1*^ΔHSC^ mice were kept on chow diet for 1 year. As shown in Supplemental Figure 4, D and E, deletion of *Itgbl1* in HSCs did not spontaneously lead to the development of liver fibrosis in mice, though hepatic levels of several liver fibrosis–related genes were slightly upregulated in *Itgbl1*^ΔHSC^ mice, suggesting that ITGBL1 exerts its function in pathogenic liver fibrosis development and progression.

### ITGBL1 suppresses HSC activation via regulation of cytoskeletal reorganization.

**Figure 2:**
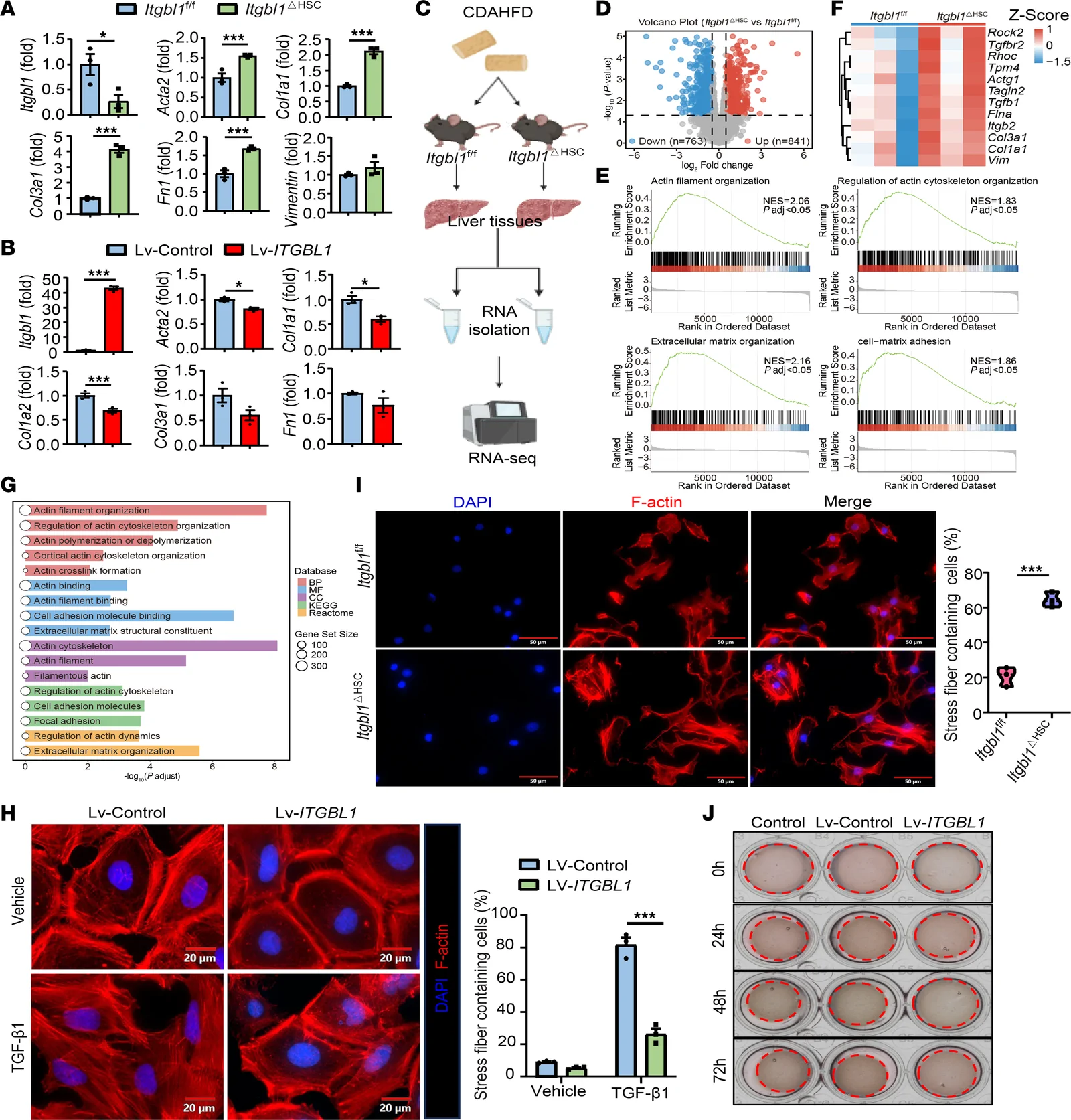
ITGBL1 inhibits liver fibrosis by regulating cytoskeleton remodeling in HSCs. (**A**) Primary HSCs were isolated from *Itgbl1*^fl/fl^ and *Itgbl1*^ΔHSC^ mice and cultured for 3 days. RT-qPCR analyses of fibrosis-related genes. (**B**) Primary mouse HSCs were isolated from WT mice and infected with Lv-*Itgbl1* for 48 hours. RT-qPCR analyses of fibrosis-related genes in Lv-*Itgbl1*–infected primary HSCs. (**C**) Regimen of RNA-seq analysis of liver tissues from CDAHFD-fed *Itgbl1*^fl/fl^ and *Itgbl1*^ΔHSC^ mice. (**D**) Volcano plot from RNA-seq analysis is shown. (**E**) GSEA of transcriptome sequencing. (**F**) Heatmap of differentially expressed genes related to cytoskeleton remodeling. (**G**) KEGG analysis of differentially expressed genes related to actin filament organization, actin binding, cytoskeleton, and extracellular matrix. BP, biological process; MF, molecular function; CC, cellular component. (**H**) LX-2 cells were infected with Lv-*ITGBL1* for 24 hours and then treated with TGF-β1 (5 ng/mL) for 24 hours. Representative images of immunofluorescence staining of F-actin (red) are shown. The percentage of stress fiber–containing cells was quantified. (**I**) Primary HSCs were isolated from *Itgbl1*^fl/fl^ and *Itgbl1*^ΔHSC^ mice and cultured for 3 days. Representative images of immunofluorescence staining of F-actin (red) are shown. The percentage of stress fiber–containing cells was quantified. (**J**) LX-2 cells were infected with Lv-*ITGBL1*. HSC contraction activity was detected by collagen-based cell contraction assay. Values represent means ± SEM. Significance was calculated using a 1-way ANOVA for multiple groups (**A**, **B**, and **I**) and a 2-tailed Student’s *t* test for comparing 2 groups (**H**). *P < 0.05, ***P < 0.001.

To further investigate the functional role of ITGBL1 in HSCs, we isolated primary HSCs from *Itgbl1*^fl/fl^ and *Itgbl1*^ΔHSC^ mice. As demonstrated in Figure 2A, primary HSCs isolated from *Itgbl1*^ΔHSC^ mice developed more fibrogenesis as evidenced by increased fibrosis-related genes in HSCs from *Itgbl1*^ΔHSC^ mice compared with HSCs from *Itgbl1*^fl/fl^ mice. Interestingly, ITGBL1 protein expression was upregulated in primary HSCs during its spontaneous activation (Supplemental Figure 5A). Furthermore, we isolated primary HSCs from C57BL/6J mice and overexpressed ITGBL1 by infecting with lentivirus-*Itgbl1* (Lv-*Itgbl1*). Notably, ITGBL1 overexpression led to a significant downregulation of fibrosis-related genes, including *Acta2*, *Col1a1*, *Col3a1*, *Col1a2*, and *Fn1*, and an obvious decrease in α-SMA expression as evidenced by RT-qPCR analysis and immunofluorescence staining (Figure 2B and Supplemental Figure 5B). To evaluate dose-dependent effects, we established HSCs with low and high levels of ITGBL1 expression. Low levels of ITGBL1 exerted modest inhibitory effects on fibrogenic gene and protein expression, whereas high levels of ITGBL1 robustly decreased their expression (Supplemental Figure 5, C and D), suggesting a dose-dependent inhibitory effect on HSC activation by ITGBL1.

Next, to determine whether ITGBL1 upregulation in HSCs is regulated by some distinct profibrotic, inflammatory, metabolic, and mechanical cues, we examined ITGBL1 expression in HSCs under corresponding stimuli including TGF-β1, interleukin-1β (IL-1β), free fatty acids (FFAs), and stromal stiffness. As shown in Supplemental Figure 6A, TGF-β1 stimulation markedly elevated ITGBL1 expression, whereas IL-1β and FFA stimuli had minimal effects. Interestingly, increased matrix stiffness led to a reduction in ITGBL1 expression in HSCs. To further confirm the specificity of TGF-β1–induced ITGBL1 upregulation, we knocked down *TGFBR2* in HSCs, which is the essential type II receptor for TGF-β signaling, and then stimulated the cells with TGF-β1. As expected, TGF-β1–mediated increase in ITGBL1 expression was significantly diminished after blocking TGF-β1 signaling (Supplemental Figure 6B). Taken together, these findings indicate that ITGBL1 is selectively regulated by TGF-β1 signaling rather than general inflammatory or metabolic stress, suggesting a profibrotic stimulus–specific regulatory pattern in activated HSCs.

To better understand how *Itgbl1* deletion in HSCs exacerbates liver fibrosis, liver tissues from CDAHFD-fed *Itgbl1*^fl/fl^ and *Itgbl1*^ΔHSC^ mice were subjected to bulk RNA-seq analysis (Figure 2C). We identified 1,604 genes that were differentially expressed in the livers of *Itgbl1*^ΔHSC^ mice, of which 841 genes were significantly upregulated and 763 were significantly downregulated (Figure 2D). Interestingly, gene set enrichment analysis (GSEA) of differentially expressed genes revealed that actin filament organization, regulation of actin cytoskeleton organization, extracellular matrix organization, and cell-matrix adhesion were significantly upregulated in *Itgbl1*^ΔHSC^ mice compared with *Itgbl1*^ΔHSC^ mice (Figure 2E). In addition, genes involved in cytoskeleton remodeling were markedly elevated in *Itgbl1*^ΔHSC^ mice (Figure 2F). Kyoto Encyclopedia of Genes and Genomes (KEGG) pathway analysis further confirmed that the differentially expressed genes were obviously enriched in signaling pathways related to cytoskeletal organization, including actin filament organization, actin binding, and cytoskeleton structural organization (Figure 2G). To further characterize the intrinsic transcriptional alterations in HSCs upon *Itgbl1* deletion during liver fibrosis, primary HSCs were isolated from CDAHFD-fed *Itgbl1*^fl/fl^ and *Itgbl1*^ΔHSC^ mice and subjected to RNA-seq analysis. Principal component analysis demonstrated a clear separation between 2 groups, indicating distinct transcriptional profiles induced by *Itgbl1* deletion in HSCs (Supplemental Figure 6C). Consistently, multiple fibrosis- and cytoskeleton-associated genes, including Col1a1, Col1a2, Col3a1, Col4a1, Loxl2, Pdgfrb, Mmp2, Mmp9, Vim, Dcn, Bgn, Postn, Tagln, and Sparc, were markedly upregulated in Itgbl1-deficient HSCs compared with controls (Supplemental Figure 6D). Furthermore, pathway enrichment analysis demonstrated that the differentially expressed genes were predominantly enriched in biological processes associated with cell adhesion, ECM organization, smooth muscle cell proliferation and development, and calcium-mediated signaling pathways (Supplemental Figure 6E), which are closely associated with cytoskeletal remodeling and mechanotransduction (20). Taken together, these findings suggest that *Itgbl1* deficiency promotes profibrotic transcriptional programs in HSCs accompanied by signaling alterations linked to cytoskeletal remodeling during liver fibrosis.

To confirm that ITGBL1 indeed influences cytoskeleton remodeling, we overexpressed ITGBL1 in HSCs. As shown in Figure 2H, overexpression of ITGBL1 substantially limited TGF-β1–induced formation of F-actin stress fibers in LX-2 cells, a well-established human HSC line. Importantly, primary HSCs isolated from *Itgbl1*^ΔHSC^ mice displayed increased F-actin stress fibers compared with those from *Itgbl1*^fl/fl^ controls (Figure 2I), reinforcing the role of ITGBL1 in modulating cytoskeletal dynamics. Since FAK signaling is known to mediate actin filament binding and promote stress fiber formation (21), we assessed the activation of FAK signaling in ITGBL1-overexpressing LX-2 cells and found that ITGBL1 overexpression markedly suppressed TGF-β1–induced FAK signaling activation (Supplemental Figure 6F), as well as the expression of the fibrotic markers Col1α1 and Col3α1 (Supplemental Figure 6G). More interestingly, collagen-based cell contraction assay demonstrated that overexpression of ITGBL1 obviously suppressed HSC contraction as evidenced by larger size of collagen gel lattices in Lv-*ITGBL1*–treated HSCs (Figure 2J).

### Spatial transcriptomics analysis reveals that Itgbl1 deficiency in HSCs worsens liver fibrosis by selectively affecting actin filament organization in a distinct HSC subset.

**Figure 3:**
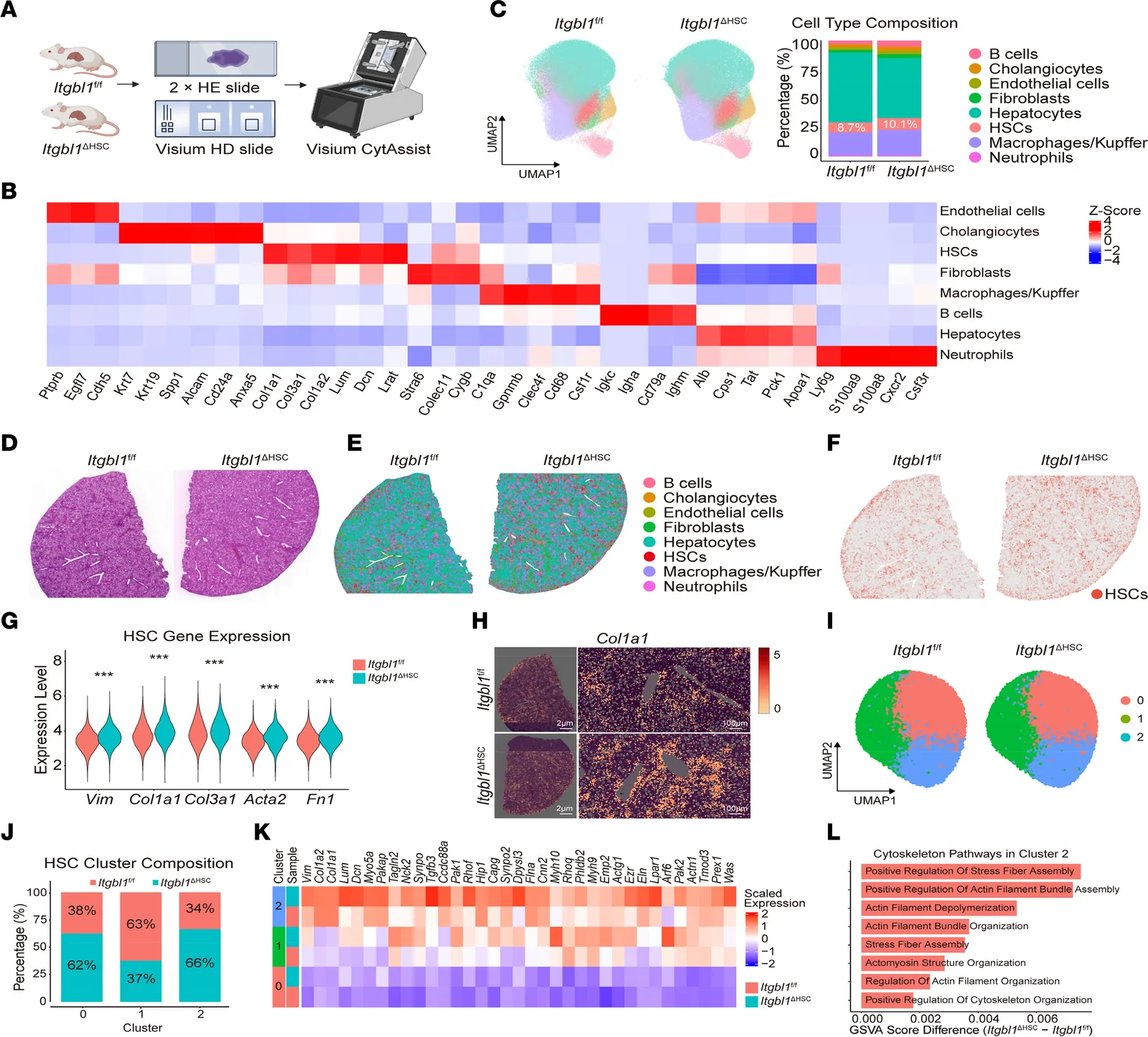
Spatial transcriptomics analysis reveals that *Itgbl1* deficiency in HSCs exacerbates liver fibrosis by exclusively disrupting actin filament organization. (**A**) Experimental workflow for spatial transcriptomics analysis. Liver tissues from *Itgbl1*^fl/fl^ and *Itgbl1*^ΔHSC^ mice were sectioned and processed using 10× Genomics Visium HD technology, followed by spatial gene expression profiling using the Visium CytAssist platform. (**B**) Heatmap shows expression profiles of canonical cell type marker genes across identified cell populations. Rows represent individual cell types. (**C**) UMAP dimensional reduction plots of integrated spatial transcriptomics data from *Itgbl1*^fl/fl^ and *Itgbl1*^ΔHSC^, with cells colored by annotated cell type identity. (**D**) H&E-stained liver sections from *Itgbl1*^fl/fl^ and *Itgbl1*^ΔHSC^ mice. (**E**) Spatial distribution of annotated cell types mapped onto tissue sections from *Itgbl1*^fl/fl^ and *Itgbl1*^ΔHSC^. Each cell is colored according to its assigned cell type identity. (**F**) Spatial distribution of HSCs (red) mapped onto tissue sections from *Itgbl1*^fl/fl^ and *Itgbl1*^ΔHSC^. Non-HSC cells are shown in gray. (**G**) Violin plots depicting expression distribution of HSC marker genes in *Itgbl1*^fl/fl^ and *Itgbl1*^ΔHSC^. The width of each violin represents the density of individual HSCs at a given expression level. Statistical comparisons performed using Wilcoxon’s rank-sum test. ***P < 0.001. (**H**) Spatial expression maps of *Col1a1* in *Itgbl1*^fl/fl^ and *Itgbl1*^ΔHSC^ liver tissues at low magnification (left) and high magnification (right) generated using Loupe Browser (10x Genomics). Expression intensity indicated by color gradient (scale: 0–5). (**I**) UMAP visualization of HSC subclusters (clusters 0–3) were identified through unsupervised clustering analysis. (**J**) Quantification of HSC subcluster composition in *Itgbl1*^fl/fl^ and *Itgbl1*^ΔHSC^. Stacked bar chart shows the percentage distribution of each cluster (clusters 0–3) within the total HSC population. (**K**) Heatmap displaying expression of cluster-specific marker genes across HSC subclusters. Color intensity represents scaled expression level. (**L**) Gene set variation analysis (GSVA) of cytoskeleton-related pathways in HSC cluster 2. Bar plot shows GSVA score differences between *Itgbl1*^ΔHSC^ and *Itgbl1*^fl/fl^ samples for indicated pathway terms.

To better elucidate how ITGBL1 affects cytoskeletal reorganization, liver tissues from CDAHFD-fed *Itgbl1*^fl/fl^ and *Itgbl1*^ΔHSC^ mice were subjected to spatial transcriptomics analysis (Figure 3A). We first performed cell type annotation based on canonical marker genes expressed across major hepatic cell populations (Figure 3B). UMAP visualization revealed distinct clustering of 8 major cell types including B cells, cholangiocytes, endothelial cells, fibroblasts, hepatocytes, HSCs, macrophages/Kupffer cells, and neutrophils in both *Itgbl1*^fl/fl^ and *Itgbl1*^ΔHSC^ samples (Figure 3C). Quantification of cell type composition demonstrated comparable distribution of major cell populations between the 2 genotypes. Representative H&E staining confirmed tissue morphology and quality for spatial transcriptomics analysis (Figure 3D). Spatial mapping demonstrated the distribution of annotated cell types across the tissue sections, revealing the architectural organization of different cell populations in both *Itgbl1*^fl/fl^ and *Itgbl1*^ΔHSC^ mice (Figure 3E). To specifically visualize HSC distribution in the spatial context, we highlighted HSCs and observed their localization (Figure 3F).

We next examined the hepatic expression of HSC marker genes in *Itgbl1*^fl/fl^ and *Itgbl1*^ΔHSC^ mice. Violin plot analysis revealed that the expression levels of key HSC markers, including Vim, Col1a1, Col3a1, Acta2, and Fn1, were significantly elevated in *Itgbl1*^ΔHSC^ samples compared with *Itgbl1*^fl/fl^ controls (Figure 3G). Notably, spatial expression maps of Col1a1 demonstrated markedly enhanced collagen deposition in fibrotic areas of *Itgbl1*^ΔHSC^ mice compared with controls at both low and high magnification (Figure 3H), further confirming the exacerbated fibrotic phenotype upon HSC-specific *Itgbl1* deletion.

To dissect HSC heterogeneity and identify specific subpopulations affected by *Itgbl1* deficiency, we performed unsupervised clustering analysis on HSCs from both genotypes, which revealed 3 distinct HSC subclusters (clusters 0–2) (Figure 3I). Quantification of HSC subcluster composition showed altered distribution patterns between *Itgbl1*^fl/fl^ and *Itgbl1*^ΔHSC^ samples, with cluster 2 showing a higher proportion in *Itgbl1*^ΔHSC^ mice (Figure 3J). Heatmap analysis of cluster-specific marker genes in cluster 2 revealed distinct transcriptional signatures between *Itgbl1*^fl/fl^ and *Itgbl1*^ΔHSC^ samples (Figure 3K). Most strikingly, gene set variation analysis (GSVA) identified remarkable enrichment of stress fiber assembly and actin filament organization pathways specifically in HSC cluster 2 from *Itgbl1*^ΔHSC^ mice, including positive regulation of stress fiber assembly and actin filament bundle assembly, actin filament depolymerization, actin filament bundle organization, stress fiber assembly, actomyosin structure organization, regulation of actin filament organization, and positive regulation of cytoskeleton organization (Figure 3L). These spatial transcriptomics findings provide compelling evidence that *Itgbl1* deficiency in HSCs preferentially disrupts cytoskeletal organization in a distinct HSC subpopulation, thereby exacerbating liver fibrosis progression. Collectively, these results reinforce our hypothesis that ITGBL1 plays a critical role in regulating cytoskeletal dynamics during HSC activation and liver fibrosis.

### ITGBL1 interacts with MYH9 in HSCs, thus affecting actin cytoskeleton organization.

**Figure 4:**
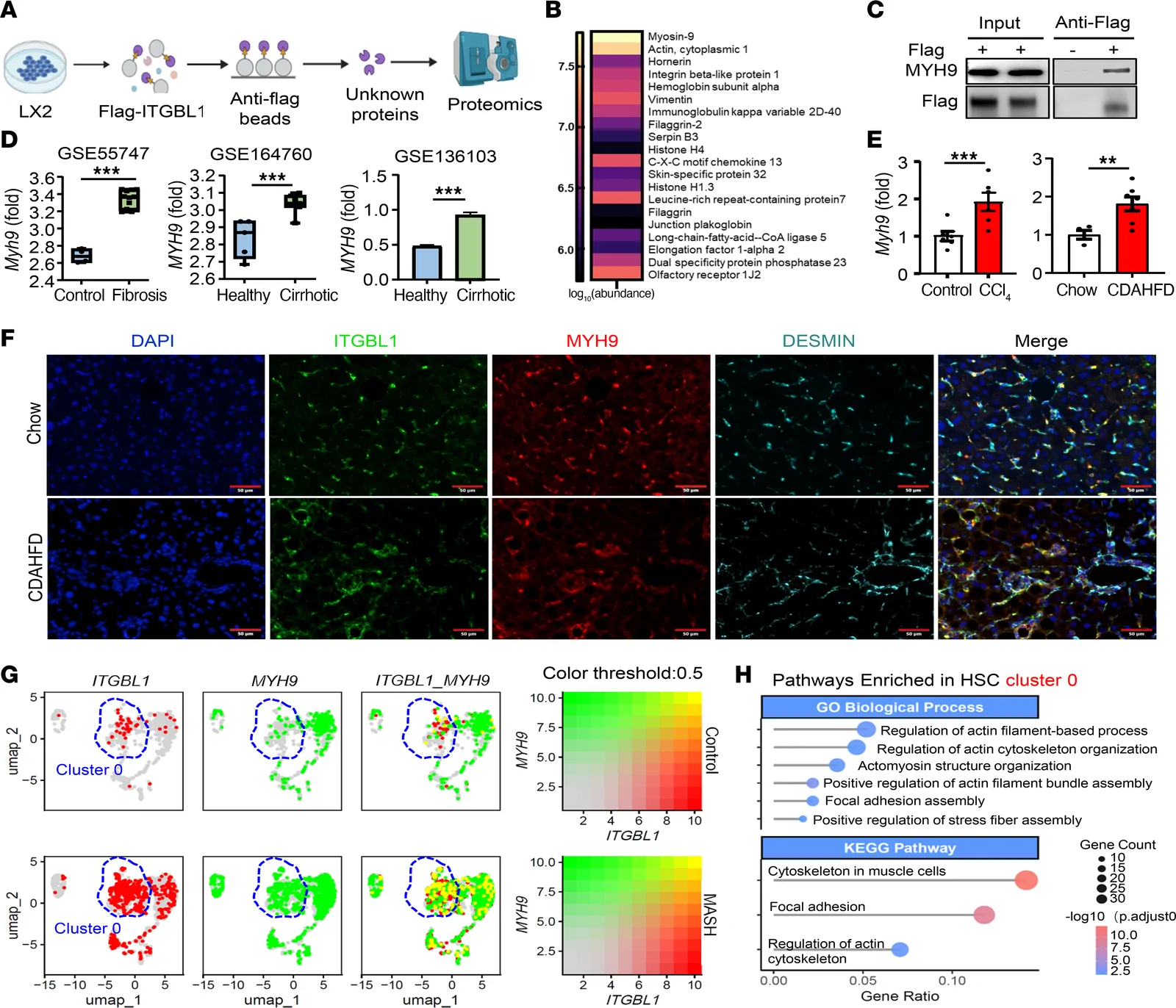
ITGBL1 interacts with MYH9 in HSCs, thus affecting actin cytoskeleton organization. (**A**) Schematic diagram of immunoprecipitation proteomics process. (**B**) Heatmap of ITGBL1-interacting proteins. (**C**) Co-IP assay was performed. Western blot analysis of protein levels of MYH9 and Flag. (**D**) Hepatic *MYH9* expression in the livers of mice with liver fibrosis and patients with liver cirrhosis was analyzed. (**E**) RT-qPCR analysis of hepatic *Myh9* mRNA levels in CCl_4_-treated and CDAHFD-fed mice. (**F**) Representative images of immunofluorescence staining of ITGBL1 (green), MYH9 (red), and desmin (teal) in the livers from CDAHFD-fed mice are shown. (**G**) UMAP plots display the coexpression analysis of *ITGBL1* and *MYH9* in HSCs from control (upper panels) and MASH human (lower panels) liver samples. The X-axis represents UMAP_1 dimensional reduction coordinate, Y-axis represents UMAP_2 coordinate. The left panels depict *ITGBL1* expression in red. The middle panels depict *MYH9* expression in green. The right panels display blended expression of both genes, where yellow indicates high coexpression of both genes. Dashed circles highlight HSC cluster 0. Color scales indicate normalized expression levels. (**H**) Gene Ontology (GO) biological process and KEGG pathway enrichment analyses were performed on differentially expressed genes in HSC cluster 0 from human liver samples. Dot size represents gene count. Color intensity indicates adjusted *P* value on a –log_10_ scale. The gene ratio on the *x* axis represents the proportion of genes enriched in each pathway. Values represent means ± SEM. Significance was calculated using a 2-tailed Student’s *t* test for comparing 2 groups (**D** and **E**). **P < 0.01, ***P < 0.001.

Emerging evidence indicates that FAK signaling pathway activation is dynamically regulated by integrin receptors, which serve as critical mediators of cell-matrix interactions (22). Recent studies have elucidated that ITGBL1 exerts modulatory effects on integrin receptor activity (17). To verify our conjecture and further elucidate the underlying mechanism by which ITGBL1 regulates cytoskeletal remodeling in HSCs, we used a lentiviral vector encoding FLAG-tagged ITGBL1. Co-immunoprecipitation (Co-IP) was performed to pull down proteins interacting with ITGBL1, and then we performed proteomics analysis (Figure 4A). Interestingly, we identified MYH9 as a potential interacting partner of ITGBL1, since MYH9 expression was the most abundant protein among these ITGBL1-interacting proteins (Figure 4B). Co-IP assay further confirmed the interaction between ITGBL1 and MYH9 in HSCs (Figure 4C). Next, we sought to determine whether MYH9 is involved in the progression of liver fibrosis. First, analysis of mouse liver fibrosis and human cirrhosis samples from the GEO database revealed a significant increase in hepatic *MYH9* expression compared with healthy controls (Figure 4D). Second, RT-qPCR analysis identified upregulation of *Myh9* mRNA levels in both CCl_4_- and CDAHFD-induced liver fibrosis mouse models (Figure 4E). scRNA-seq analysis from the Liver Cell Atlas database also revealed that hepatic *MYH9* expression in humans was high in HSCs (Supplemental Figure 7A). Notably, immunohistochemistry and immunofluorescence staining demonstrated that MYH9 expression was significantly elevated in CDAHFD- and CCl_4_-induced liver fibrosis (Supplemental Figure 7, B and C) and predominantly expressed in HSCs (Supplemental Figure 7, D and E). More importantly, MYH9 colocalized with ITGBL1 in HSCs in experimental mouse liver fibrosis models (Figure 4F and Supplemental Figure 7F).

To define whether ITGBL1 coexpressed with MYH9 in a distinct HSC subset in liver tissues from patients with MASH, we analyzed a scRNA-seq dataset (GSE212837) from a public database (23). As shown in Supplemental Figure 8A, *ITGBL1* was mainly expressed in HSCs and positively correlated with *MYH9*. Next, we obtained 4 HSC subsets (cluster 0–3) (Supplemental Figure 8B). Interestingly, *ITGBL1* was exclusively expressed in HSC cluster 0 and cluster 2 while *MYH9* was broadly expressed in all HSC clusters (Supplemental Figure 8C). More interestingly, a strong positive correlation between *ITGBL1* and *MYH9* was observed in HSC cluster 0 (Figure 4G). Most importantly, pathways enriched in HSC cluster 0 were associated with regulation of actin filament organization, regulation of actin cytoskeleton organization, and positive regulation of stress fiber assembly (Figure 4H). All of the above findings further support the interaction between ITGBL1 and MYH9 in a distinct subset of HSCs.

### Myh9 deficiency in HSCs alleviates liver fibrosis in CDAHFD-fed mice.

**Figure 5:**
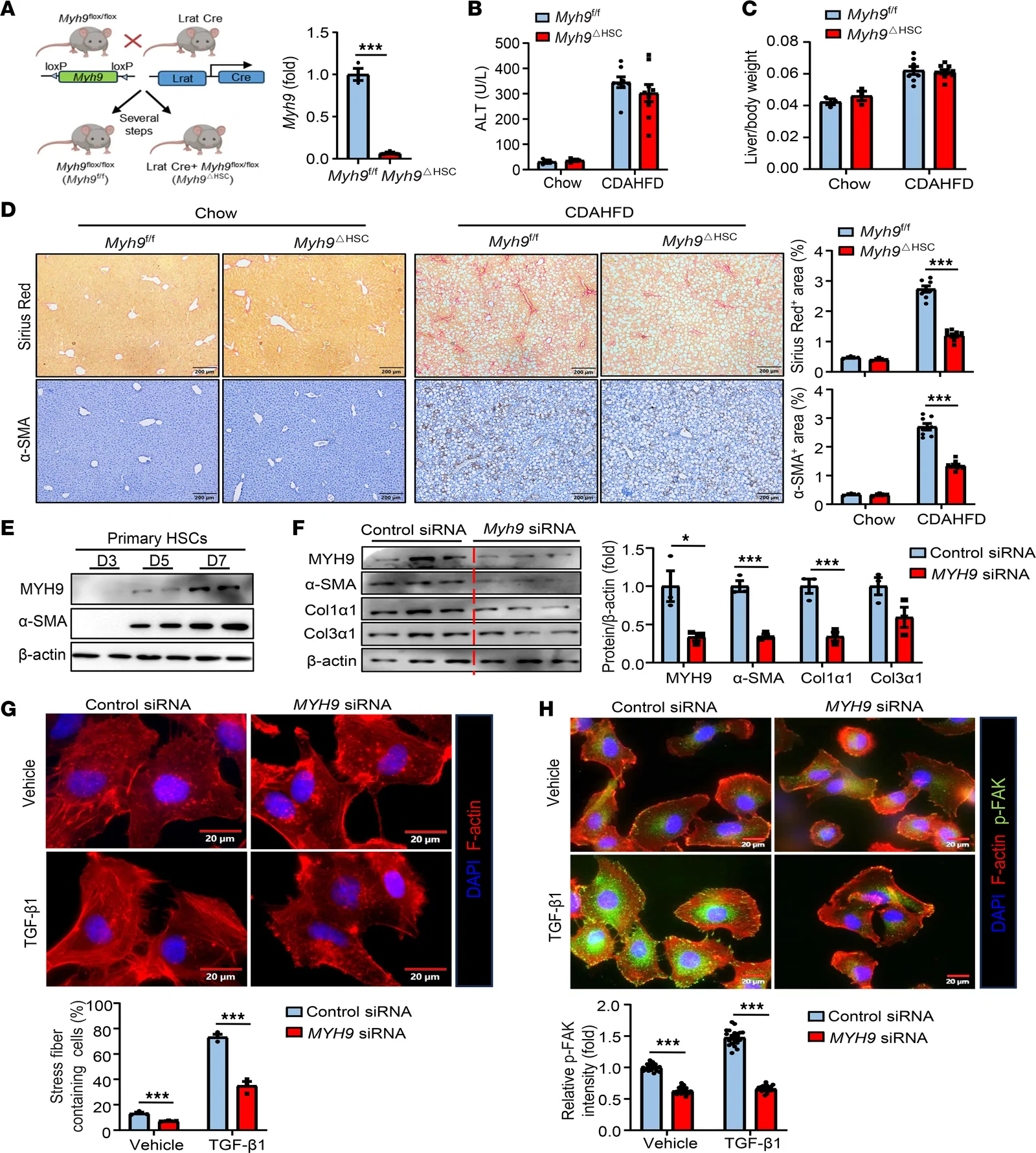
*Myh9* deficiency in HSCs ameliorates liver fibrosis in CDAHFD-fed mice. (**A**) *Myh9*-floxed mice were crossed with Lrat Cre mice for several generations to generate *Myh9*^ΔHSC^ mice. Knockout efficiency was verified by RT-qPCR analysis by isolating primary mouse HSCs from *Myh9*^fl/fl^ and *Myh9*^ΔHSC^ mice. (**B**) Serum alanine aminotransferase (ALT) levels were measured. (**C**) Liver/body weight ratio was analyzed. (**D**) Representative images of Sirius red and α-SMA staining are shown. The degree of liver fibrosis was quantified. (**E**) Primary HSCs were isolated from WT mice and cultured for 3, 5, 7 days. MYH9, α-SMA, and β-actin protein levels were measured. (**F**) Primary mouse HSCs were isolated from WT mice and transfected with *Myh9* siRNA to silence MYH9 expression. The expression of different proteins was measured by Western blotting. Relative protein expression was quantified. (**G** and **H**) LX-2 cells were transfected with *MYH9* siRNA for 24 hours and treated with TGF-β1 (5 ng/mL) for 24 hours. Representative images of immunofluorescence of F-actin (red) are shown. The percentage of stress fiber–containing cells was quantified (**G**). Representative images of immunofluorescence of p-FAK (green) and F-actin (red) are shown. Relative p-FAK intensity was quantified (**H**). Values represent means ± SEM. Significance was calculated using a 1-way ANOVA for multiple groups (**B**–**D**, **G**, and **H**) and a 2-tailed Student’s *t* test for comparing 2 groups (**F**). *P < 0.05, ***P < 0.001.

MYH9, also known as NMHC IIA, is a part of the actomyosin complex that generates mechanical force and tension to propel the sliding of actin filaments, thus being involved in essential cellular functions, including cell adhesion, cytokinesis, cell migration, and the maintenance of cell shape and polarity (24, 25). However, whether MYH9 in HSCs affects the development of liver fibrosis remains largely unknown. To answer this critical question, we generated HSC-specific *Myh9*-knockout mice (*Myh9*^ΔHSC^) and validated the knockout efficiency in HSCs (Figure 5A). *Myh9*^fl/fl^ and *Myh9*^ΔHSC^ were subjected to CDAHFD feeding for 4 weeks (Supplemental Figure 9A). As shown in Figure 5, B–D, and Supplemental Figure 9B, although serum ALT levels, liver/body ratio, and the degree of liver steatosis were comparable between *Myh9*^fl/fl^ mice and *Myh9*^ΔHSC^ mice after CDAHFD feeding, deletion of *Myh9* in HSCs markedly ameliorated liver fibrosis as evidenced by a significant reduction in Sirius red–positive and α-SMA–positive areas in the livers of *Myh9*^ΔHSC^ mice compared with *Myh9*^fl/fl^ mice. In addition, several fibrogenesis-related genes including *Col1a1*, *Col3a1*, and *Col4a1* were significantly downregulated in CDAHFD-fed *Myh9*^ΔHSC^ mice compared with CDAHFD-fed *Myh9*^fl/fl^ mice (Supplemental Figure 9C).

To further clarify the function of MYH9 in HSCs, we measured MYH9 expression during HSC activation and found that MYH9 expression was substantially elevated during HSC activation (Figure 5E). Interestingly, siRNA-mediated knockdown of *Myh9* in primary mouse HSCs decreased the expression of several fibrosis-related proteins, suggesting that MYH9 promoted HSC activation in vitro (Figure 5F). Subsequently, knockdown of *MYH9* in LX-2 cells also resulted in a significant reduction in the expression of fibrosis-associated genes at both the mRNA and protein levels (Supplemental Figure 10, A and B). More importantly, immunofluorescence analysis further revealed that silencing of *MYH9* in HSCs inhibited the TGF-β1–induced formation of F-actin stress fibers (Figure 5G), which was accompanied by reduced activation of the FAK signaling pathway (Figure 5H). Meanwhile, knockdown of *MYH9* suppressed TGF-β1–induced activation of the FAK signaling pathway and collagen synthesis (Supplemental Figure 10C). All these findings suggest that MYH9 elevation in HSCs exacerbates liver fibrosis.

### ITGBL1 impairs actomyosin assembly by blocking actin binding to myosin via promoting MYH9 phosphorylation.

**Figure 6:**
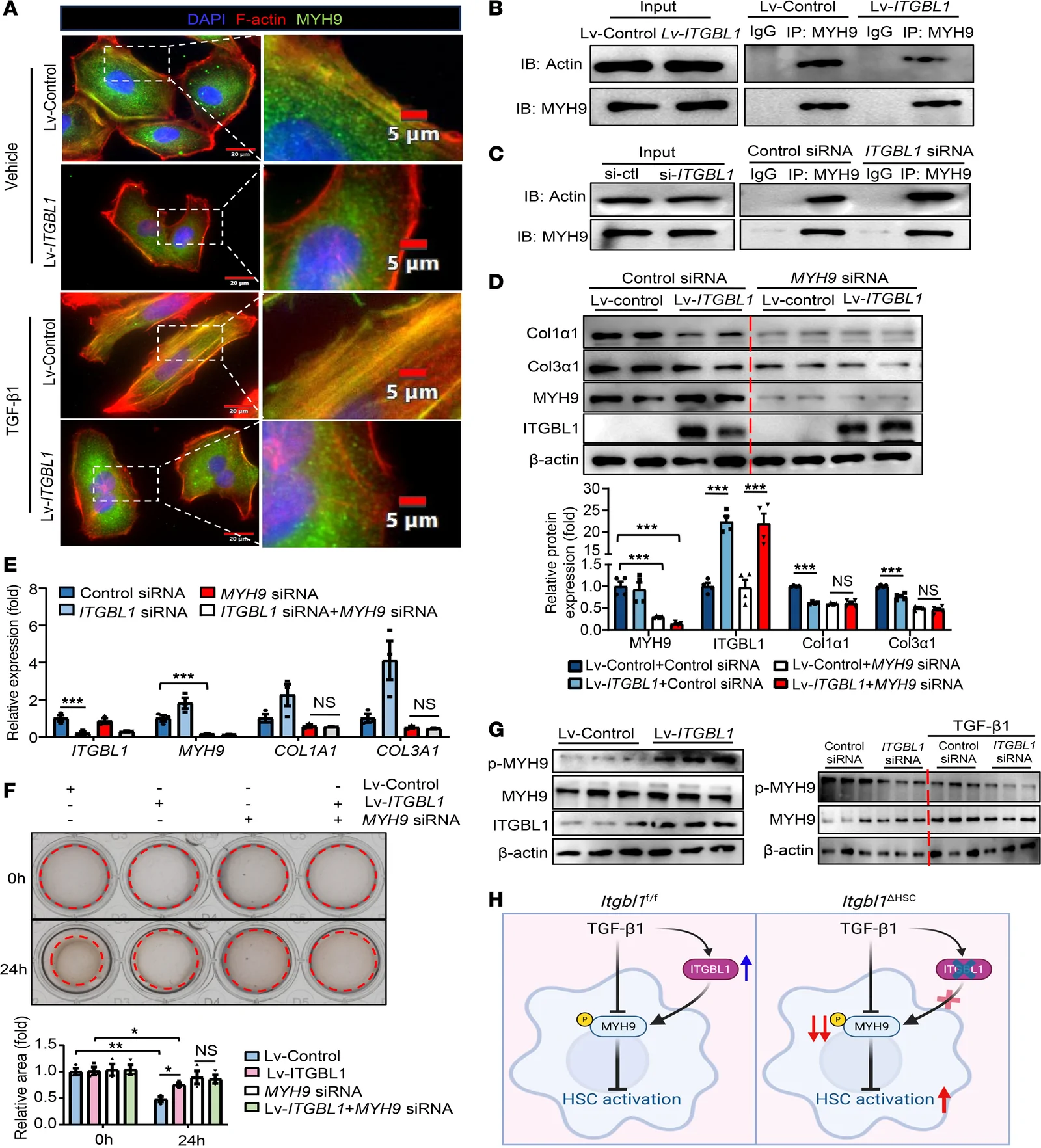
ITGBL1 disrupts actomyosin assembly by promoting MYH9 phosphorylation. (**A**) LX-2 cells were infected with Lv*-ITGBL1* for 48 hours and then treated with TGF-β1 (5 ng/mL) for 24 hours. Representative images of immunofluorescence of MYH9 (green) and F-actin (red) are shown. (**B**) Co-IP assay was performed in *ITGBL1*-overexpressed LX-2 cells. Western blot analysis of protein levels of MYH9 and actin. (**C**) Co-IP assay was performed in *ITGBL1* siRNA-infected LX-2 cells. Western blot analysis of protein levels of MYH9 and actin. (**D**) LX-2 cells were infected with Lv*-ITGBL1* and/or *MYH9* siRNA for 48 hours. The expression of different proteins was measured by Western blotting. Relative protein expression was quantified. (**E**) LX-2 cells were infected with *ITGBL1* siRNA and/or *MYH9* siRNA for 48 hours. The expression of different genes was measured by RT-qPCR analysis. (**F**) LX-2 cells were infected with Lv*-ITGBL1* and/or *MYH9* siRNA for 48 hours, and then these infected HSCs were mixed with Collagen Gel Working Solution. HSC contraction activity was measured by collagen-based cell contraction assay. (**G**) LX-2 cells were infected with Lv*-ITGBL1* or *ITGBL1* *siRNA* for 48 hours. The expression of different proteins was measured by Western blotting. Relative protein expression was quantified. (**H**) Scheme depicting the ITGBL1-MYH9 interaction in HSCs. TGF-β1 stimulation suppresses MYH9 phosphorylation (p-MYH9) and elevates ITGBL1 expression. This increase in ITGBL1 then forms a feedback loop that limits HSC overactivation by upregulating p-MYH9 levels. Conversely, *ITGBL1* deficiency lowers p-MYH9 levels, thus promoting HSC activation. Values represent means ± SEM. Significance was calculated using a 1-way ANOVA for multiple groups (**D**–**F**) and a 2-tailed Student’s *t* test for comparing 2 groups (**G**). *P < 0.05, **P < 0.01, ***P < 0.001.

Since MYH9 binding to actin filaments forms actomyosin cytoskeleton, and transcriptomics data above indicate that ITGBL1 may affect this process, we reasoned that ITGBL1 might influence actomyosin formation in HSCs, thus suppressing HSC activation. To test this hypothesis, we determined actomyosin assembly in HSCs after overexpression of ITGBL1 by immunofluorescence double-staining of MYH9 and F-actin. As shown in Figure 6A and Supplemental Figure 11A, ITGBL1 overexpression robustly disrupted the formation of actomyosin clusters, whereas *ITGBL1* knockdown enhanced MYH9–F-actin colocalization. To further validate this observation, we performed dose-dependent overexpression of ITGBL1. As shown in Supplemental Figure 11B, increasing ITGBL1 levels progressively disrupted actomyosin structures and markedly reduced the colocalization of MYH9 with F-actin. Moreover, *ITGBL1* knockout (both in vitro and in vivo) increased MYH9 expression, whereas *MYH9* knockdown did not affect ITGBL1 expression (Supplemental Figure 11, C and D). More importantly, Co‑IP assays showed that overexpression of ITGBL1 markedly reduced the interaction between MYH9 and F-actin, whereas knockdown of *ITGBL1* enhanced MYH9–F-actin binding (Figure 6, B and C), suggesting that ITGBL1 regulates myosin assembly by modulating the binding of MYH9 to F-actin. Furthermore, knockdown of *MYH9* substantially abolished ITGBL1-mediated antifibrotic effect in vitro (Figure 6D). In contrast, *ITGBL1* knockdown in HSCs increased the expression of fibrosis genes, which was also eliminated after MYH9 silencing (Figure 6E), suggesting that ITGBL1 inhibits HSC activation by disrupting MYH9-dependent myosin remodeling. Moreover, cell contraction assay showed that *MYH9* knockdown limited HSC contraction. Importantly, ITGBL1 suppressed HSC contraction in a MYH9-dependent manner (Figure 6F). Previous studies suggested that MYH9 phosphorylation is involved in actomyosin assembly (7, 24). Therefore, we examined MYH9 activation status under fibrotic stimulation. In both TGF-β1–treated HSCs and liver tissues from experimental liver fibrosis models, phosphorylated MYH9 (p-MYH9) was found to be markedly decreased compared with control conditions, suggesting a context-dependent regulation of MYH9 activation during liver fibrogenesis (Supplemental Figure 11, E and F). This is consistent with previous studies demonstrating that the phosphorylation state of NM II (MYH9) is dynamically regulated and closely associated with changes in cytoskeletal organization and cellular contractile activity (24). Strikingly, ITGBL1 overexpression promoted, while *ITGBL1* knockdown suppressed, the phosphorylation of MYH9 on Ser1943 (Figure 6G and Supplemental Figure 11G). Given that FOXQ1 and SNAIL have been previously reported as downstream transcriptional targets of ITGBL1 in other cellular contexts (26), we tried to examine their expression in HSCs. However, no significant changes were observed under ITGBL1 manipulation (Supplemental Figure 11H), suggesting that ITGBL1-mediated regulation in HSCs is primarily associated with cytoskeletal signaling rather than previously described transcriptional programs. Collectively, our results indicated that ITGBL1 inhibits HSC activation through impairing MYH9 actomyosin remodeling via promoting MYH9 phosphorylation at Ser1943, ultimately diminishing HSC contraction and fibrosis-related gene expression (Figure 6H).

### Itgbl1 deficiency in HSCs worsens liver fibrosis in a MYH9-dependent manner in vivo.

**Figure 7:**
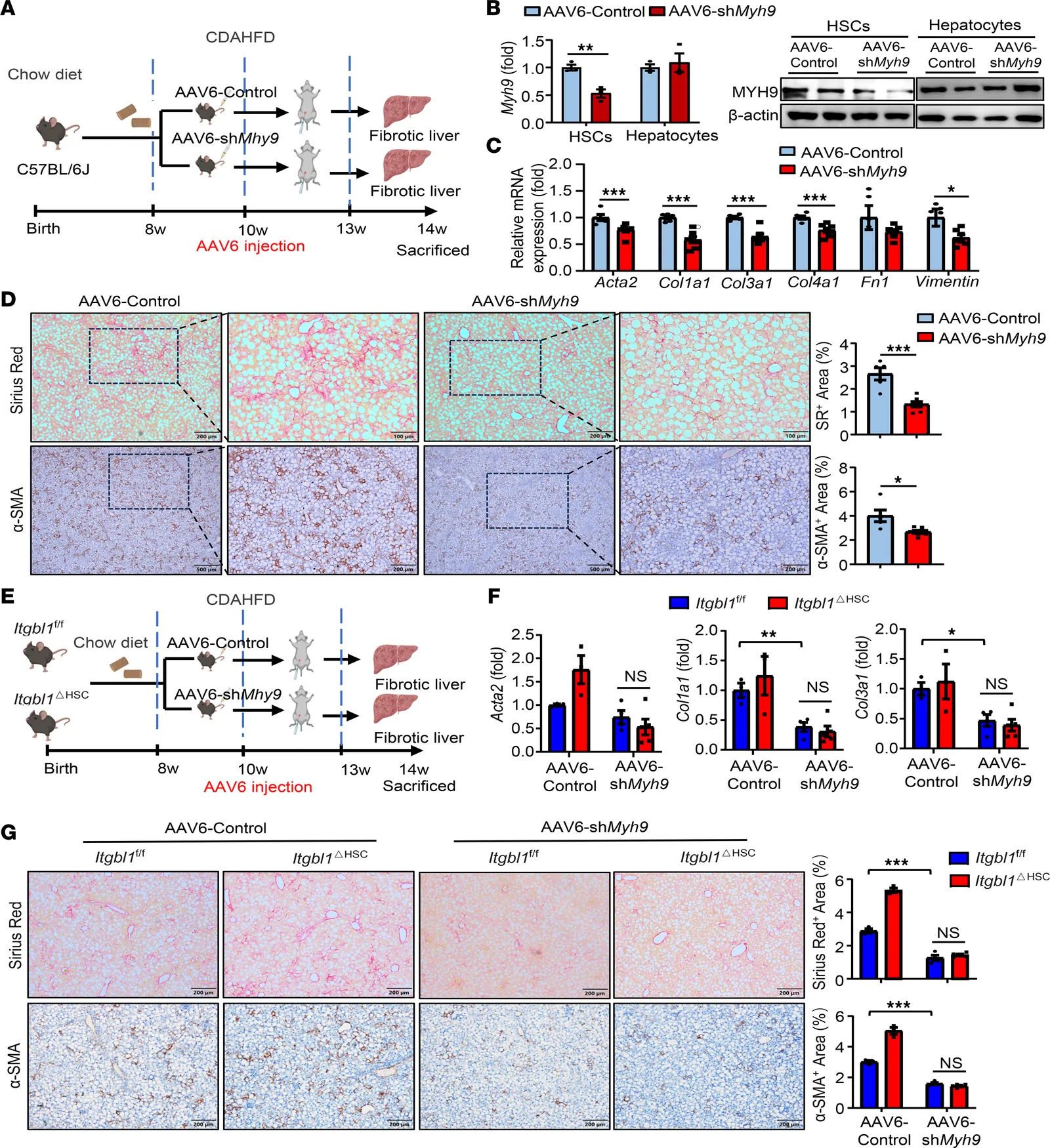
*Itgbl1* deficiency in HSCs worsens liver fibrosis in a MYH9-dependent manner. (**A**) The regimen of targeting HSCs to specifically knock down *Myh9* levels by injection of AAV6. (**B**) Primary HSCs and hepatocytes were isolated after CDAHFD feeding for 4 weeks. The levels of MYH9 mRNA and protein were measured by RT-qPCR analysis and Western blotting. (**C**) RT-qPCR analyses of fibrosis-related genes in AAV6-control– and AAV6-sh*Myh9*–infected mice. (**D**) Representative images of Sirius red staining and α-SMA staining are shown. The degree of liver fibrosis was quantified. (**E**) *Itgbl1*^fl/fl^ or *Itgbl1*^ΔHSC^ mice were first fed a CDAHFD for 2 weeks, followed by tail vein injection of AAV6-control or AAV6-sh*Myh9*, and then maintained on CDAHFD for an additional 3 weeks. (**F**) RT-qPCR analyses of fibrosis-related genes in AAV6-control– and AAV6-sh*Myh9*–infected *Itgbl1*^fl/fl^ or *Itgbl1*^ΔHSC^ mice after CDAHFD feeding. (**G**) Representative images of Sirius red staining and α-SMA staining are shown. The degree of liver fibrosis was quantified. Values represent means ± SEM. Significance was calculated using a 1-way ANOVA for multiple groups (**F** and **G**) and a 2-tailed Student’s *t* test for comparing 2 groups (**B**–**D**). *P < 0.05, **P < 0.01, ***P < 0.001.

The above results indicate that ITGBL1 is elevated in HSCs during liver fibrosis progression and such feedback elevation suppresses liver fibrosis, suggesting that targeting ITGBL1 may not be a therapeutic strategy for treatment of liver fibrosis. Nevertheless, MYH9 is upregulated in liver fibrosis and exacerbates liver fibrosis. Therefore, we tried to silence *Myh9* expression in HSCs in vivo to test the possibility that targeting MYH9 ameliorates liver fibrosis. Accumulating evidence suggests that adeno-associated virus 6 (AAV6) exhibits organ tropism toward activated HSCs in mice (27, 28). To further elucidate the role of MYH9 in HSCs and liver fibrosis, we specifically downregulated *Myh9* in HSCs by injection with AAV6-sh*Myh9* during CDAHFD feeding (Figure 7A). Immunofluorescence staining first confirmed the efficient knockdown of MYH9 in HSCs (Supplemental Figure 12A). In addition, as shown in Figure 7B, both Western blotting and qPCR analyses further confirmed a significant reduction of MYH9 expression in HSCs following AAV6-sh*Myh9* treatment compared with the control group, whereas MYH9 expression remained unchanged in hepatocytes, indicating selective knockdown efficiency in HSCs without detectable off-target effects in hepatocytes. Additionally, *Myh9* knockdown in HSCs did not mitigate liver injury, as serum ALT levels were comparable between the 2 groups (Supplemental Figure 12B), and the expression of fibrosis-related genes and proteins, including *Acta2*, *Col1a1*, *Col3a1*, and *Col4a1*, was significantly decreased after *Myh9* knockdown in HSCs (Figure 7, C and D, and Supplemental Figure 12C). Additionally, *Myh9* knockdown in HSCs did not mitigate liver injury and steatosis, though for several inflammatory and lipogenic genes, including *Il1b*, *Tnfa*, *Srebp1*, and *Acc1*, mRNA levels were reduced in AAV6-sh*Myh9*–injected mice after CDAHFD feeding (Supplemental Figure 12, D and E).

Similarly, we assessed the therapeutic effect of AAV6-sh*Myh9* on CCl_4_-induced liver fibrosis (Supplemental Figure 13, A and B). As expected, AAV6-sh*Myh9* evidently mitigated CCl_4_-induced liver fibrosis as demonstrated by lower levels of Sirius red– and α-SMA–positive areas and fibrosis-related genes in AAV6-sh*Myh9–*treated mice compared with AAV6-control group (Supplemental Figure 13, C–E). Taken together, these findings strongly indicate that targeting inhibition of MYH9 in HSCs effectively alleviates experimental liver fibrosis.

To define whether the exacerbated liver fibrosis in *Itgbl1*^ΔHSC^ mice is dependent on MYH9, *Itgbl1*^fl/fl^ and *Itgbl1*^ΔHSC^ mice were injected with AAV6-sh*Myh9* (Figure 7E). Strikingly, AAV6-sh*Myh9* injection counteracted the exacerbated liver fibrosis in *Itgbl1*^ΔHSC^ mice as evidenced that the expression of several fibrogenic genes, including *Acta2*, *Col1a1*, and *Col3a1*, was comparable between *Itgbl1*^fl/fl^ and *Itgbl1*^ΔHSC^ mice after injection with AAV6-sh*Myh9* in CDAHFD-induced MASH (Figure 7F). Consistently, in AAV6-control–injected mice, *Itgbl1* deficiency in HSCs markedly exacerbated liver fibrosis; however, this exacerbated liver fibrosis was abolished in *Itgbl1*^ΔHSC^ mice after injection with AAV6-sh*Myh9* as indicated by Sirius red and α-SMA staining (Figure 7G). These key findings demonstrate that silencing *Myh9* in HSCs diminishes the aggravation of liver fibrosis caused by *Itgbl1* deficiency, indicating that the profibrotic effect of *Itgbl1* deletion in HSCs is largely dependent on MYH9.

### Hepatic expression of ITGBL1 and MYH9 is elevated and positively correlates with HSC activation and cytoskeleton remodeling in patients with liver fibrosis.

**Figure 8:**
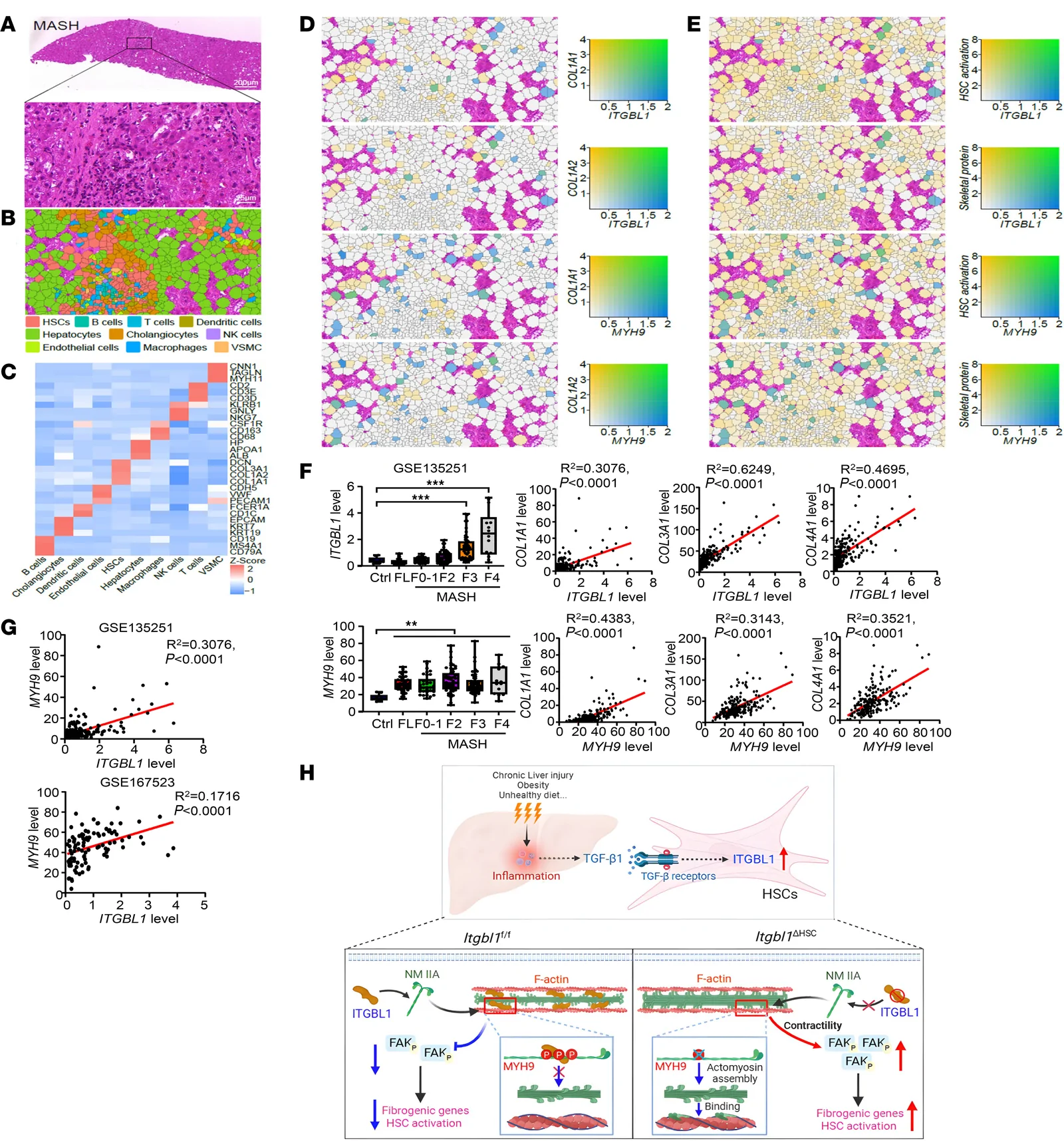
Hepatic *ITGBL1* and *MYH9* levels are highly elevated and positively correlated with hepatic expression of liver fibrogenic genes in patients with MASH. (**A**) Representative H&E-stained section of human MASH liver tissue is shown for spatial transcriptomics analysis. The upper panel displays the full tissue section with a boxed region indicating the area of interest (scale bar: 200 μm). The lower panel shows a magnified view of the boxed region displaying detailed histological features (scale bar: 25 μm). (**B**) Spatial distribution of major cell types in the MASH liver tissue section is displayed following cell segmentation (scale bar: 25 μm). (**C**) Heatmap displays differential gene expression patterns across the cell types. Color intensity indicates *Z*-score, where red indicates upregulation and blue indicates downregulation. (**D**) Spatial expression maps show coexpression patterns of *ITGBL1*, *COL1A1*, *MYH9*, and *COL1A2* across the tissue section. Each spot represents a cell, with color indicating expression level based on the gradient scale shown on the right, where green indicates high coexpression of both genes. Scale bar: 25 μm. (**E**) Spatial expression maps show coexpression patterns of *ITGBL1*, *MYH9* with HSC activation markers and skeletal protein markers. (**F**) Hepatic levels of *ITGBL1*, *MYH9*, and fibrogenic genes in healthy controls and patients with MASH were analyzed in a public dataset (GSE135251). (**G**) Correlation between *ITGBL1* levels and *MYH9* levels was analyzed in 2 public datasets (GSE135251 and GSE167523). (**H**) Scheme depicting a critical role of ITGBL1-MYH9 interaction during liver fibrosis development. During chronic liver injury or obesity, activated inflammatory cell– or HSC-released TGF-β1 elevates ITGBL1 expression in HSCs, which impairs actomyosin assembly by blocking the binding of MYH9 to F-actin, thus suppressing actomyosin contractility-mediated FAK signaling activation. Such ITGBL1-mediated cytoskeleton reorganization suppression limits HSC activation and liver fibrogenesis. Correlations were calculated using Pearson’s *r*. Significance was calculated using a 1-way ANOVA for multiple groups (**F**). **P < 0.01, ***P < 0.001.

To investigate the clinical relevance of our findings, hepatic *ITGBL1* and *MYH9* levels in lean and obese individuals were analyzed from public scRNAseq data from Liver Cell Atlas database. As demonstrated in Supplemental Figure 14A, *ITGBL1* and *MYH9* levels in HSCs were elevated in obese individuals compared with lean individuals. Next, we performed spatial transcriptomics analysis on liver tissue collected from patients with MASH (Figure 8A). Following cell segmentation, we mapped the spatial distribution of major cell types, including HSCs, hepatocytes, B cells, T cell, dendritic cells, cholangiocytes, NK cells, endothelial cells, macrophages, and VSMCs, revealing the cellular architecture of MASH liver tissue (Figure 8B). The expression patterns of canonical lineage markers confirmed successful identification of major cell populations within the dataset. Hepatocytes showed strong expression of *ALB*, *APOA1*, and *HP*, whereas cholangiocytes exhibited high levels of *KRT7*, *KRT19*, and *EPCAM*. HSCs displayed enriched expression of ECM-related genes, including *COL1A1*, *COL1A2*, *COL3A1*, and *DCN*. Immune subsets were clearly separated transcriptionally, with T cells (*CD3D*, *CD3E*, *CD2*), NK cells (*KLRB1*, *NKG7*, *GNLY*), macrophages (*CD68*, *CD163*, *CSF1R*), dendritic cells (*CD1C*, *FCER1A*), and B cells (*CD19*, *CD79A*, *MS4A1*) exhibiting distinct marker profiles. Endothelial cells expressed *PECAM1*, *CDH5*, and *VWF*, while VSMCs showed selective expression of *TAGLN*, *CNN1*, and *MYH11*, collectively demonstrating robust cell type segregation (Figure 8C). Interestingly, *ITGBL1* and *MYH9* levels positively correlated with *COL1A1* and *COL3A1* levels in HSCs of liver tissue from patients with MASH (Figure 8D). Most importantly, a strong correlation between *ITGBL1/MYH9* levels and genes related with HSC activation/cytoskeleton remodeling was observed in HSCs of liver tissue from patients with MASH (Figure 8E).

Next, we further confirmed these findings in 2 distinct public datasets (GSE135251 and GSE167523). In patients with severe MASH-related liver fibrosis, hepatic *ITGBL1* levels were significantly elevated and positively correlated with the expression of several fibrotic marker genes, including *COL1A1*, *COL3A1*, and *COL4A1* (Figure 8F and Supplemental Figure 14B). Meanwhile, hepatic *MYH9* levels were robustly upregulated in patients with fatty liver and MASH and also positively correlated with the expression of *COL1A1*, *COL3A1*, and *COL4A1* (Figure 8F and Supplemental Figure 14C). More importantly, hepatic *ITGBL1* levels positively correlated with *MYH9* levels in MASH (Figure 8G). Taken together, these human data emphasize our experimental observations and underscore the clinical significance of the ITGBL1-MYH9 axis in HSC activation and liver fibrosis progression.

## Discussion

Liver fibrosis is an early stage of cirrhosis that is potentially reversible. Current therapies fail to cure liver fibrosis, emphasizing the need to identify new antifibrotic approaches. Liver fibrosis is characterized by the excessive accumulation of ECM and resultant stiffening of tissue stroma (29, 30). This stiffening appropriates actomyosin-mediated mechanical tension, ultimately affecting HSC fate decision and function (31). Despite great advances in understanding the pathogenesis of liver fibrosis, the regulators controlling actomyosin assembly and contractility in HSCs, thereby influencing liver fibrosis, remain largely unknown. In this study, we demonstrated that ITGBL1 limits HSC activation by preferentially corrupting MYH9-dependent actomyosin assembly, ultimately impeding liver fibrosis progression. Our study highlights the importance of the ITGBL1-MYH9 interaction as a critical brake to limit liver fibrosis, which provides a promising strategy for the treatment of this malady (Figure 8H).

One interesting finding from the current study is that ITGBL1 is a negative regulator of mechanotransduction pathways during HSC activation by impairing actomyosin assembly. ITGBL1 was previously thought to be related to integrin regulation and TGF-β signaling pathway (32); its exact effect on liver fibrosis remains unclear. Our study reveals an unexpected antifibrotic effect of ITGBL1 and challenges the popular concept that it mainly inhibits scar formation (33, 34). Although ITGBL1 expression in HSCs is elevated during liver fibrosis, bulk RNA-seq and spatial transcriptomics analysis strongly suggest that such elevation forms negative feedback to limit HSC activation by exclusively disrupting actomyosin contractility, thus preventing liver fibrosis progression (35). During liver injury, quiescent HSCs transdifferentiate into contractile, ECM-producing myofibroblast-like cells. This process is driven largely by actin cytoskeletal remodeling, which in turn promotes ECM synthesis and secretion through several pathways such as FAK, RhoA/ROCK, and YAP/TAZ (29). Our findings redefine ITGBL1 as an autocrine inhibitor of activated HSCs, which extends the understanding of cytoskeleton dynamic regulation. Specifically, the enhanced cytoskeleton-related gene expression and stress fiber formation were observed in the absence of ITGBL1 in HSCs, together with elevated FAK activity, suggesting that ITGBL1 suppresses dysregulated actin remodeling and integrin-associated signaling. Conversely, its overexpression restrains TGF-β1–driven cytoskeletal reorganization, thereby slowing down the profibrotic signaling transduction. Notably, mechanotransduction has been implicated in regulating intrahepatic hemodynamics and portal hypertension during liver fibrosis (36). However, our data suggest that *Itgbl1* deletion in HSCs did not substantially alter spleen weight despite pronounced effects on cytoskeletal remodeling and fibrosis progression, which may be because spleen weight is a late and indirect surrogate with limited sensitivity, especially in models with moderate portal pressure elevation. This apparent dissociation also supports the notion that portal hypertension is a multifactorial process driven not only by HSC contractility, but also by ECM deposition, vascular remodeling, sinusoidal capillarization, endothelial dysfunction, and global changes in intrahepatic blood flow (37). Most interestingly, spatial transcriptomics analysis reveals that genes related to profibrogenesis and actin filament organization in a specific HSC cluster are much higher in *Itgbl1*-deficient HSCs than in control HSCs, indicating that ITGBL1 preferentially affects cytoskeletal remodeling in HSCs during liver fibrosis progression. Taken together, these insights emphasize that ITGBL1-mediated control of cytoskeletal remodeling — rather than ECM deposition alone — represents a central brake to limit HSC activation and liver fibrogenesis.

Actomyosin contractile force produced by myosin II molecules that bind and pull actin filaments is harnessed for diverse processes, such as cell adhesion, cell migration, and cell division (38). Like muscle myosin II, NM II molecules are composed of 3 pairs of peptides: 2 heavy chains of 230 kDa, two 20 kDa regulatory light chains (RLCs) that regulate NM II activity, and two 17 kDa essential light chains that stabilize the heavy chain structure (8). Three genes in mammalian cells, *MYH9*, *MYH10*, and *MYH14*, encode the NMHC II proteins, which interact through their coiled-coil domains and contain actin-binding and ATPase activities in their head domains (8). At the molecular level, NM II proteins drive actomyosin contraction by sliding actin filaments past one another using energy produced by ATP hydrolysis. Contractile forces allow cells to sense the stiffness of their environment, which affects cell migration, cell shape dynamics, as well as cellular fate decisions (39). Accumulating studies suggest that NM II–mediated cytoskeleton reorganization is involved in fibrotic diseases and cancer (40). Despite an increasingly refined understanding of contractility and detailed knowledge of the molecular composition of the actomyosin contractility, the physical mechanisms controlling actomyosin assembly in HSCs during liver fibrosis development remain insufficiently understood. In this study, we performed multitranscriptomic analyses to demonstrate that ITGBL1 exclusively disrupts actomyosin assembly via blocking actin-myosin binding, thereby impairing actomyosin contractility-driven profibrotic response. To further dissect the underlying mechanism, we further performed proteomics analysis and identified MYH9 — a NMHC — as a direct binding partner of ITGBL1. MYH9 is a well-known regulator of actin filament assembly, cell contractility, and migration (41), and it has been shown to promote fibronectin secretion through integrin-Talin interactions (42). Although MYH9 is implicated in cytoskeletal remodeling in other contexts (43), its role in HSCs and liver fibrosis remains unknown. Our study identifies MYH9 as a crucial regulator to promote HSC activation. Specifically, MYH9 enhances mechanotransduction by promoting stress fiber formation and adhesion dynamics, reinforcing HSC activation and ECM deposition in a feed-forward loop. Most importantly, spatial and single-cell transcriptomics analyses on liver tissues from human MASH samples and mouse models strongly suggest that ITGBL1 positively correlates with MYH9 in a distinct HSC population, which is closely related to actin filament organization. Therefore, the ITGBL1-MYH9 interaction is a central mechanoregulator that controls cytoskeletal equilibrium and mechanical homeostasis in HSCs, thus affecting liver fibrosis progression. In addition, it is important to note that in the healthy liver, although MYH9 is expressed but not activated, the low basal level of ITGBL1 in HSCs is not sufficient to drive cytoskeletal remodeling or HSC activation. Therefore, the effect of ITGBL1 on MYH9-dependent actomyosin dynamics likely depends on a profibrotic microenvironment.

Additionally, our findings should be considered in conjunction with the recently emerged concept that quiescent HSCs actively contribute to liver homeostasis and liver protection, rather than merely serving as passive vitamin A storage cells. Recent studies have demonstrated that HSCs maintain liver integrity by producing key paracrine mediators, including hepatocyte growth factor, bone morphogenetic protein 9/10, and RSPO3 (44–46). In this context, our study adds a new layer to this protective network by identifying ITGBL1 as a differentially regulated factor primarily acting at the intracellular mechanotransduction level. Unlike traditional HSC-derived liver-protective factors that support hepatocyte function and liver homeostasis, ITGBL1 acts as an inducible autocrine brake, limiting the excessive activation of HSCs by inhibiting MYH9-dependent actin-myosin assembly and downstream mechanosensitive signaling. Therefore, ITGBL1 does not replace these established homeostatic regulators, but rather complements them by specifically controlling the cytoskeletal structure and mechanotransduction state of HSCs during the injury response. This makes ITGBL1 a component in the HSC-based protective system, functioning as a “mechanical checkpoint” to prevent the continuous activation of profibrotic signals. Specifically, the experiments on functional loss and gain further elucidated the relationship between ITGBL1 and MYH9. Knockout of *ITGBL1* significantly increased the expression of MYH9 in cultured HSCs, while *MYH9* knockout did not affect ITGBL1 expression. Although these findings indicate a clear one-way association, they do not necessarily indicate a direct regulatory relationship between these 2 molecules. On the contrary, the increase in MYH9 expression after depletion of *ITGBL1* may represent an indirect, context-dependent response of activated HSCs to altered cytoskeleton and fibrosis signaling states. At the subcellular level, immunofluorescence staining revealed distinct localization patterns of ITGBL1 and MYH9 in HSCs. MYH9 was predominantly localized in the cytoplasm and enriched along F-actin–positive filamentous structures, indicating its close association with the actin cytoskeleton. In contrast, ITGBL1 exhibited a more diffuse cytoplasmic and pericellular distribution with less apparent colocalization with F-actin. Functionally, *ITGBL1* knockdown increased the association between MYH9 and F-actin, whereas MYH9 knockdown had minimal effect on the distribution of ITGBL1 relative to actin. These observations suggest that MYH9 is directly engaged in cytoskeletal organization, whereas ITGBL1 likely regulates this process through modulation of MYH9-actin interaction.

While ITGBL1 plays a protective role in liver fibrosis, its functions are highly dependent on tissue microenvironment. In some tissues, ITGBL1 promotes fibroblast activation via TGF-β signaling (32), and it is upregulated in hepatocellular carcinoma (47), where it may support tumor progression. These contrasting roles suggest that ITGBL1 function is influenced by tissue context, expression patterns, and interaction partners. Therefore, although ITGBL1 shows promising potential as a therapeutic target, its application is complicated due to its pleiotropic and tissue-specific effects. A deeper understanding of ITGBL1’s regulation and its downstream effectors will be critical to guide its therapeutic development. In contrast, MYH9 may represent a more available target. As a core regulator of actin contractility, MYH9 expression was visibly elevated in activated HSCs and plays a central role in driving their morphological changes, migration, and fibrotic activity. Targeting MYH9 to inhibit cytoskeletal remodeling may offer a specific and effective strategy to limit HSC activation and slow down fibrosis progression. In support of this concept, AAV6-mediated MYH9 silencing effectively attenuated liver fibrosis in our models. Notably, the therapeutic efficacy of this strategy may depend on the activation status of HSCs within the fibrotic microenvironment. Previous studies have shown that AAV6 preferentially transduces activated HSCs in fibrotic liver, whereas its transduction efficiency in quiescent HSCs of normal liver is substantially lower (28). Consistently, in our study, a high dose of AAV6-sh*Myh9* was administered after fibrosis induction, when HSC activation had already been established, thereby facilitating selective targeting in activated HSCs. Furthermore, qPCR and Western blot analyses confirmed efficient *Myh9* knockdown in HSCs but not hepatocytes. Notably, although AAV6-sh*Myh9* reduced *Myh9* expression by only approximately 50% due to its high basal levels, it still has a strong antifibrotic effect, suggesting that complete suppression of MYH9 is not required to disrupt profibrotic mechanotransduction. Instead, partial inhibition appears sufficient to impair actomyosin organization and attenuate FAK-dependent signaling, thereby limiting fibrogenesis. Collectively, these findings suggest that the therapeutic efficacy of AAV6-based approaches is closely linked to the activation state of HSCs and further underscore MYH9 as a promising mechanotransduction-related therapeutic target in liver fibrosis.

Although our findings underscore the key antifibrotic role of ITGBL1-MYH9 interaction in liver fibrosis progression, our study has some limitations. First, we provide several lines of evidence to support the conclusion that ITGBL1 in HSCs limits liver fibrosis by disrupting MYH9-dependent actomyosin assembly via promoting MYH9 phosphorylation; however, the in-depth mechanism by which ITGBL1 promotes MYH9 phosphorylation remains unknown. In addition to NM II RLC phosphorylation, NM II filament assembly is also controlled by phosphorylation of the NMHC II coiled-coil and tail domains, playing an important role in regulating actomyosin contractility-driven mechanotransduction (48). MYH9 phosphorylation in different sites affects actomyosin assembly. There are several phosphorylation sites near the C-termini of NMHCs, in both the coiled-coil domain and the nonhelical tail, including sites that are phosphorylated by protein kinase C, casein kinase II, and transient receptor potential melastatin 7 (7). Of note, it was reported that MYH9 phosphorylation on Ser1943 limits actomyosin assembly (24). Therefore, additional studies will be required to unravel how ITGBL1 promotes MYH9 phosphorylation. Second, whether ITGBL1 in fibroblasts also influences other organ fibrosis remains an open and compelling question and warrants further investigation. To answer this interesting question, we tried to generate fibroblast-specific *Itgbl1*-deficient mice by crossing *Igtbl1*^fl/fl^ mice with Col2a1-Cre mice. Unfortunately, the homozygote conditional knockout mice are not viable and fertile due to an unknown reason. It is plausible to predict that *Itgbl1* deletion in fibroblasts may worsen other organ fibrosis, since activated HSCs are well known to transform into myofibroblast-like cells during liver fibrosis (4).

In conclusion, our study identifies ITGBL1 as an autocrine brake to limit HSC activation and uncovers a previously unrecognized ITGBL1/MYH9/FAK signaling axis that orchestrates cytoskeletal remodeling during liver fibrosis. While ITGBL1 shows therapeutic potential, its complex roles across diseases and tissues present challenges for clinical application. Nevertheless, MYH9 may represent a promising and more specific antifibrotic target. Taken together, our findings lay a conceptual foundation for the development of targeted therapies aimed at cytoskeletal remodeling in liver fibrosis, offering new avenues for molecular medicine in fibrotic disease.

## Methods

### Sex as a biological variable.

Our study exclusively examined male mice because female mice are resistant to the development of key features of human MASH-related liver fibrosis following MASH diet feeding. It is unknown whether the findings are relevant for female mice. Primary HSCs were isolated from both male and female mice in this study.

### Mice and experimental mouse models.

Male 8- to 10-week C57BL/6J mice were purchased from The Jackson Laboratory. *Itgbl1*-floxed mice (*Itgbl1*^fl/fl^, strain no. T015994) and Lrat-P2A-iCre mice (strain no. T006205) were purchased from GemPharmatech (Nanjing, China). *Myh9*-floxed mice (*Myh9*^fl/fl^, strain no. S-CKO-03859) were purchased from Cyagen Biosciences (Suzhou, China). HSC-specific *Itgbl1-* or *Myh9*-knockout mice (*Itgbl1*^ΔHSC^ or *Myh9*^ΔHSC^) were generated by crossing Lrat Cre with *Itgbl1*^fl/fl^ or *Myh9*^fl/fl^ mice for several generations. For the CCl_4_-induced liver fibrosis, mice were injected with 2 mL/kg of 10% CCl_4_ (catalog O815211, MACKLIN, Shanghai, China) in olive oil (catalog C805325, MACKLIN, Shanghai, China) twice per week. The mice were sacrificed 48 hours after the last injection of CCl_4_. For the CDAHFD-induced MASH-related liver fibrosis, mice were fed with CDAHFD (catalog A06071302, Research Diets, Inc.) for 4 weeks. For the AAV6-sh*Myh9*–treated experiments, a recombinant AAV6 containing mouse *Myh9* shRNA was purchased from Hanheng Biotechnology Co., Ltd. (Shanghai, China). Mice were pretreated with CCl_4_ or fed CDAHFD for 2 weeks before administering AAV6-sh*Myh9* (3 × 10^11^ viral genomes) or AAV6-negative control (nontargeting control) (3.5 × 10^11^ viral genomes) via tail vein injection. The animals were housed in temperature-controlled, specific pathogen–free conditions with a 12-hour light/dark cycle and had unrestricted access to food and water.

### Visium HD spatial transcriptomics.

The FFPE liver samples from *Itgbl1*^fl/fl^ and *Itgbl1*^ΔHSC^ mice were subjected to Visium spatial transcriptomics analysis by Majorbio Bio-pharm Technology Co., Ltd. (Shanghai, China). Briefly, after dissection, target liver regions were fixed in 10% neutral-buffered formalin for 24 hours, embedded in paraffin, and sectioned at 5 μm. Sections were mounted onto glass slides (Sigma-Aldrich, P0425) and processed following the 10x Genomics standard protocol (CG000684), including deparaffinization, H&E staining, and antigen retrieval. Spatial transcriptomic profiling was performed according to the manufacturer’s Visium HD Gene Expression workflow (CG000685) using the Visium HD Gene Expression Slide (10x Genomics, PN-1000185) and the Visium HD Gene Expression Reagent Kit, Mouse (10x Genomics, CG0006855). Probe hybridization, extension/ligation, and library construction were performed as described in the Visium HD FFPE protocol. Libraries were quality-checked on a Tapestation (Agilent 5300) and quantified by qPCR before sequencing on a NovaSeq X Plus platform (150 bp paired-end reads) with a target sequencing depth of approximately 150 Gb per capture area. Quality control, gene quantification, and data analysis of spatial transcriptomics data are described in Supplemental Methods. The mouse spatial transcriptomics can be accessed under the Genome Sequence Archive (accession number: PRJCA065569).

Liver tissue from a patient with MASH obtained from Peking University First Hospital was subjected to spatial transcriptomics analysis. The human spatial transcriptomics can be accessed under the Genome Sequence Archive (accession number: PRJCA043049). Spatial coexpression analysis was performed using Loupe Browser to assess the relationship between *ITGBL1* and fibrosis-related genes. Gene pairs were selected based on biological relevance, including collagen genes and cytoskeletal markers. For each comparison, spatial coexpression matrices and maps were generated, and expression values were visualized using the default continuous scale provided by the software.

### Statistics.

Data are expressed as the means ± SEM for each group and were analyzed using GraphPad Prism software. A 2-tailed unpaired Student’s *t* test was performed to compare values obtained from 2 groups; data from multiple groups were compared with 1-way ANOVA with Tukey’s post hoc comparison test. A *P* value less than 0.05 was considered significant.

### Study approval.

All animal experiments were approved by the Institutional Animal Care and Use Committee of the SIMM, Chinese Academy of Sciences, and conducted in accordance with institutional animal care ethical guidelines. The human spatial transcriptomics study was approved by Peking University First Hospital (2023-yan-520).

Additional methods are included in the Supplemental Methods.

### Date availability.

All raw spatial transcriptomic sequencing data have been deposited in the Genome Sequence Archive (GSA) at the China National Center for Bioinformation (CNCB) under accession numbers PRJCA065569 (mouse hepatic spatial transcriptome dataset) and PRJCA043049 (human MASH liver spatial transcriptome dataset). Bulk RNA-seq data generated from mouse fibrotic liver tissues have been deposited in the NCBI Sequence Read Archive (SRA) database under BioProject accession PRJNA1472899. Values for all data points found in graphs can be found in the Supporting Data Values file.

## Author contributions

YL designed and conducted the experiments and wrote the paper. YW, CT, and XF collected human liver samples; performed and analyzed spatial transcriptomics, scRNA-seq data, and bulk RNA-seq data; and edited the manuscript. NM, Y Hao, ZF, SL, ZY, HL, SG, SY, PX, and SD conducted some experiments and edited the manuscript. AG edited the manuscript. YD and Y He obtained funding, supervised the whole project, and wrote the paper. All authors approved the final manuscript.

During the preparation of this manuscript, ChatGPT 3.5 was used solely for language editing and proofreading. The prompts were used to revise the manuscript with standard academic English expressions, correct grammatical mistakes, and improve readability, and keep all experimental data, research results, professional terms and core conclusions unchanged.

## Conflict of interest

The authors have declared that no conflict of interest exists.

## Funding support

- The National Key Research and Development Program of China (2023YFA1800804 to YH).
- The Strategic Priority Research Program of the Chinese Academy of Sciences (XDB0830402 to YH).
- The Noncommunicable Chronic Diseases-National Science and Technology Major Project (2024ZD0530604 to YH).
- The National Natural Science Foundation of China (U22A20272 to YD, 82270601 to YH, 82300516 to ZY).

## Supplementary Material

Supplemental data

Unedited blot and gel images

Supporting data values

## Version 1 07/16/2026

In-Press Preview

## Version 2 09/15/2026

Electronic publication

## References

[1] Hammerich L, Tacke F (2023). Hepatic inflammatory responses in liver fibrosis. Nat Rev Gastroenterol Hepatol.

[2] Kisseleva T, Brenner D (2020). Molecular and cellular mechanisms of liver fibrosis and its regression. Nat Rev Gastroenterol Hepatol.

[3] Yang Z (2025). Steatotic liver disease and cancer: from pathogenesis to therapeutic targets. eGastroenterology.

[4] Schwabe RF, Brenner DA (2025). Hepatic stellate cells: balancing homeostasis, hepatoprotection and fibrogenesis in health and disease. Nat Rev Gastroenterol Hepatol.

[5] Romet-Lemonne G (2025). Mechanics of single cytoskeletal filaments. Annu Rev Biophys.

[6] Niu C (2024). The role of the cytoskeleton in fibrotic diseases. Front Cell Dev Biol.

[7] Vicente-Manzanares M (2009). Non-muscle myosin II takes centre stage in cell adhesion and migration. Nat Rev Mol Cell Biol.

[8] Garrido-Casado M (2021). Nonmuscle myosin II regulation directs its multiple roles in cell migration and division. Annu Rev Cell Dev Biol.

[9] Even-Ram S (2007). Myosin IIA regulates cell motility and actomyosin-microtubule crosstalk. Nat Cell Biol.

[10] Newell-Litwa KA (2015). Non-muscle myosin II in disease: mechanisms and therapeutic opportunities. Dis Model Mech.

[11] Murrell M (2015). Forcing cells into shape: the mechanics of actomyosin contractility. Nat Rev Mol Cell Biol.

[12] Mitten EK, Baffy G (2022). Mechanotransduction in the pathogenesis of non-alcoholic fatty liver disease. J Hepatol.

[13] Kai F (2025). Tension-induced organelle stress: an emerging target in fibrosis. Trends Pharmacol Sci.

[14] Tsuchida T, Friedman SL (2017). Mechanisms of hepatic stellate cell activation. Nat Rev Gastroenterol Hepatol.

[15] Long Y (2021). Mechanical communication in fibrosis progression. Trends Cell Biol.

[16] Takagi J (2001). Definition of EGF-like, closely interacting modules that bear activation epitopes in integrin beta subunits. Proc Natl Acad Sci U S A.

[17] Song EK (2018). ITGBL1 modulates integrin activity to promote cartilage formation and protect against arthritis. Sci Transl Med.

[18] Huang W (2020). ITGBL1 promotes cell migration and invasion through stimulating the TGF-β signalling pathway in hepatocellular carcinoma. Cell Prolif.

[19] Gallage S (2022). A researcher’s guide to preclinical mouse NASH models. Nat Metab.

[20] Saraswathibhatla A (2023). Cell-extracellular matrix mechanotransduction in 3D. Nat Rev Mol Cell Biol.

[21] Tan X (2023). Focal adhesion kinase: from biological functions to therapeutic strategies. Exp Hematol Oncol.

[22] Dawson JC (2021). Targeting FAK in anticancer combination therapies. Nat Rev Cancer.

[23] Wang S (2023). An autocrine signaling circuit in hepatic stellate cells underlies advanced fibrosis in nonalcoholic steatohepatitis. Sci Transl Med.

[24] Feroz W (2024). Non-muscle myosin II A: friend or foe in cancer?. Int J Mol Sci.

[26] Zhu H (2022). Integrin subunit β-like 1 mediates angiotensin II-induced myocardial fibrosis by regulating the forkhead box Q1/Snail axis. Arch Biochem Biophys.

[27] Chen H (2020). Targeting Nestin+ hepatic stellate cells ameliorates liver fibrosis by facilitating TβRI degradation. J Hepatol.

[28] Rezvani M (2016). In vivo hepatic reprogramming of myofibroblasts with AAV vectors as a therapeutic strategy for liver fibrosis. Cell Stem Cell.

[29] Mascharak S (2024). Modelling and targeting mechanical forces in organ fibrosis. Nat Rev Bioeng.

[30] Wade H (2024). Mechanistic role of long non-coding RNAs in the pathogenesis of metabolic dysfunction-associated steatotic liver disease and fibrosis. eGastroenterology.

[32] Li XQ (2015). ITGBL1 Is a Runx2 transcriptional target and promotes breast cancer bone metastasis by activating the TGFβ signaling pathway. Cancer Res.

[33] Kim S-E (2024). Novel integrated multiomics analysis reveals a key role for integrin beta-like 1 in wound scarring. EMBO Rep.

[34] Zhao J (2023). Mechanical pressure-induced dedifferentiation of myofibroblasts inhibits scarring via SMYD3/ITGBL1 signaling. Dev Cell.

[35] Tomasek JJ (2002). Myofibroblasts and mechano-regulation of connective tissue remodelling. Nat Rev Mol Cell Biol.

[36] Hilscher MB (2019). Mechanical stretch increases expression of CXCL1 in liver sinusoidal endothelial cells to recruit neutrophils, generate sinusoidal microthombi, and promote portal hypertension. Gastroenterology.

[37] Felli E (2023). Mechanobiology of portal hypertension. JHEP Rep.

[38] Thiyagarajan S (2022). Myosin turnover controls actomyosin contractile instability. Proc Natl Acad Sci U S A.

[39] Koenderink GH, Paluch EK (2018). Architecture shapes contractility in actomyosin networks. Curr Opin Cell Biol.

[40] Ciszewski WM (2021). Cytoskeleton reorganization in endMT-the role in cancer and fibrotic diseases. Int J Mol Sci.

[41] Lin X (2020). Silencing MYH9 blocks HBx-induced GSK3β ubiquitination and degradation to inhibit tumor stemness in hepatocellular carcinoma. Signal Transduct Target Ther.

[42] Wang X (2024). Talin2 binds to non-muscle myosin IIa and regulates cell attachment and fibronectin secretion. Sci Rep.

[43] Lee S (2023). Identification of MYH9 as a key regulator for synoviocyte migration and invasion through secretome profiling. Ann Rheum Dis.

[44] Filliol A (2022). Opposing roles of hepatic stellate cell subpopulations in hepatocarcinogenesis. Nature.

[45] Zhao D (2024). GDF2 and BMP10 coordinate liver cellular crosstalk to maintain liver health. Elife.

[46] Sugimoto A (2025). Hepatic stellate cells control liver zonation, size and functions via R-spondin 3. Nature.

[47] Ye F (2021). Serum levels of ITGBL1 as an early diagnostic biomarker for hepatocellular carcinoma with hepatitis B virus infection. J Hepatocell Carcinoma.

[48] Chinthalapudi K, Heissler SM (2024). Structure, regulation, and mechanisms of nonmuscle myosin-2. Cell Mol Life Sci.

