# Supplementary material for: ITGBL1-MYH9 interaction in hepatic stellate cells acts as a mechanoregulator controlling liver fibrosis in mice
Source: J Clin Invest. 2026 Jul 16;136(18):e203412. doi: 10.1172/JCI203412 (PMC13574165; doi:10.1172/JCI203412)
Supplement: Supplementary file 1 [file jci-136-203412-s055.pdf]

Supporting Materials

ITGBL1-MYH9 interaction in hepatic stellate cells acts as a mechano-regulator controlling liver fibrosis in mice

Short title: ITGBL1-MYH9 interaction in HSCs mitigates liver fibrosis

Yixin Li[1, 2#], Yan Wang[3#], Chenhao Tong[4#], Xinghuan Fu[3#], Ningning Ma[2], Yawen Hao[2, 5], Zian Feng[1], Shijia Ling[6], Zequn Yin[1], Haodong Li[7], Shujun Ge[6], Siting Yang[6], Peng Xiao[8], Siyue Dong[6], Adrien Guillot[9], Yajun Duan[1*], Yong He[2, 5, 6*]

[1]The First Affiliated Hospital of USTC, Division of Life Sciences and Medicine, University of Science and Technology of China (USTC), Hefei, Anhui, China; [2]State Key Laboratory of Drug Research, Shanghai Institute of Materia Medica (SIMM), Chinese Academy of Sciences, Shanghai, China; [3]Department of Infectious Diseases, Peking University First Hospital, Beijing, China; [4]Hepatopancreatobiliary Surgery Department, the First Affiliated Hospital of Ningbo University, Ningbo, Zhejiang, China; [5]University of Chinese Academy of Sciences, Beijing, China; [6]School of Chinese Materia Medica, Nanjing University of Chinese Medicine, Nanjing, China; [7]Tianjing University of Traditional Chinese Medicine, Tianjin, China; [8]Department of Hepatology, the First Hospital of Jilin University, Changchun, China; [9]Department of Hepatology & Gastroenterology, Charité-Universitätsmedizin Berlin, Campus Virchow-Klinikum and Campus Charité Mitte, Berlin, Germany.

[#]Yixin Li, Yan Wang, Chenhao Tong and Xinghuan Fu contributed equally to this work.

Methods and Materials

Spatial transcriptomics

Quality control and gene quantification of spatial transcriptomic data

Raw sequencing data were processed using Space Ranger (v4.0.3) with the Visium Mouse Transcriptome probe set v2.0 and the GRCm39 reference genome. The pipeline performed demultiplexing, image alignment, barcode processing, and probe-to-gene assignment to generate feature–barcode count matrices. Using spatial barcode information, sequencing reads were assigned to individual 2 × 2 μm squares, which represent

the smallest analytical unit of the Visium HD platform. To balance spatial resolution and data density, these primary grids were aggregated into larger bins of 8 × 8 μm and 16 × 16 μm for downstream analyses. At each resolution (2 μm, 8 μm, and 16 μm), key quality control metrics were computed to evaluate overall sample performance, including the total number of bins, paired reads per bin, number of detected genes, and unique molecular identifiers (UMIs) per bin.

Data analysis for spatial transcriptomic data

The filtered data from both *Itgbl1*$^{f/f}$ and *Itgbl1*$^{\Delta HSC}$ samples were integrated using the Seurat package (version 5.0) in R (version 4.3.1). Data normalization was performed using the NormalizeData function with default parameters. Highly variable features (n=3000) were identified using the FindVariableFeatures function, followed by data scaling using the ScaleData function. Principal component analysis (PCA) was performed using RunPCA with 30 principal components, and dimensionality reduction was conducted using Uniform Manifold Approximation and Projection (UMAP) with the first 15 principal components. Unsupervised clustering was performed using the FindNeighbors and FindClusters functions with a resolution of 0.5.

Cell-type annotation was performed based on the expression of canonical marker genes. Hepatocytes were identified by high expression of hepatocyte markers (*Alb*, *Apoa1*, *Apob*, *Ttr*, *Hp*, *Hnf4a*); HSCs and fibroblasts by expression of *Col1a1*, *Col3a1*, *Lrat* and *Dcn*" macrophages/Kupffer cells by *Cd68*, *Clec4f*, and *C1qa*; endothelial cells by *Ptprb*, *Egfl 7*, *Cdh5*; cholangiocytes by *Krt19*, *Krt7*, *Ptprb*; B cells by *Igkc*, *Igha*, *Cd79a* and *Ms4a1*; and neutrophils by *S100a8*, *S100a9*, *Csf3r*, and *Cxcr2*. Cell-type composition was quantified and compared between *Itgbl1*$^{f/f}$ and *Itgbl1*$^{\Delta HSC}$ samples.

For HSC-specific analysis, HSC populations were extracted from the integrated dataset and re-clustered using the same workflow described above. Differential gene expression analysis between *Itgbl1*$^{f/f}$ and *Itgbl1*$^{\Delta HSC}$ HSCs was performed using the FindAllMarkers function with parameters: only.pos = TRUE, min.pct = 0.20, and logfc.threshold = 0.25. Violin plots were generated to visualize the expression of HSC marker genes (*Vim*, *Col1a1*, *Col3a1*, *Acta2*, *Fn1*) across samples using the VlnPlot function. Statistical comparisons were performed using the Wilcoxon rank-sum test.

HSC subclustering analysis identified four distinct HSC subpopulations (Clusters 0-3). Cluster-specific marker genes were identified using FindAllMarkers, and the top 30 marker genes per cluster were selected for heatmap visualization using the ComplexHeatmap package. Gene Set Variation Analysis (GSVA) was performed to assess pathway enrichment in HSC subpopulations. Gene sets related to cytoskeleton

organization were obtained from the Molecular Signatures Database (MSigDB, version 7.5) using the msigdbr package, including Gene Ontology Biological Process (GO: BP), KEGG, and Reactome pathways. The following cytoskeleton-related gene sets were analyzed: actin filament-based movement, actin filament organization, lamellipodium assembly, Rho GTPase cycle, regulation of actin filament-based processes, stress fiber assembly, focal adhesion assembly, and Rho GTPases activate formins. GSVA scores were calculated using the GSVA package (version 1.48.0) with method = "gsva", kcdf = "Gaussian", min.sz = 5, and max.sz = 500. Differential GSVA scores between *Itgbl1*$^{f/f}$ and *Itgbl1*$^{\Delta HSC}$ samples were compared using Student's t-test, and P values were adjusted for multiple testing using the Benjamini-Hochberg method.

Spatial co-expression analysis was performed using Loupe Browser to assess the relationship between *ITGBL1* and fibrosis-related genes. Gene pairs were selected based on biological relevance, including collagen genes and cytoskeletal markers. For each comparison, spatial co-expression matrices and maps were generated, and expression values were visualized using the default continuous scale provided by the software.

Liver tissue from patient with MASH obtained from Peking University First Hospital was subjected to spatial transcriptomics analysis. The human spatial transcriptomics can be accessed under the Genome Sequence Archive (accession number: PRJCA043049). The study was approved by Peking University First Hospital (2023-yan-520). The human FFPE tissue section was subjected to H&E staining to visualize tissue morphology and pathological features characteristic of MASH. Cell segmentation was performed using Loupe Browser (10x Genomics) to identify and delineate individual cells within the tissue section based on their spatial coordinates and morphological characteristics.

Cell-type annotation was performed based on the expression patterns of canonical marker genes for major hepatic cell populations. Hepatocytes were defined by high expression of *ALB*, *APOA1*, and *HP*, while cholangiocytes were identified by *KRT7*, *KRT19*, and *EPCAM*. Hepatic stellate cells were annotated based on extracellular matrix–associated genes including *COL1A1*, *COL1A2*, *COL3A1* and *DCN*. Immune cell subsets were distinguished using lineage-restricted markers, including *CD3D*, *CD3E*, and *CD2* for T cells, *KLRB1*, *NKG7* and *GNLY* for NK cells, *CD68*, *CD163*, and *CSF1R* for macrophages, *CD1C* and *FCER1A* for dendritic cells, and *CD19*, *CD79A* and *MS4A1* for B cells. Endothelial cells were defined by expression of *PECAM1*, *CDH5*, and *VWF*, and vascular smooth muscle cells (VSMCs) were identified by *TAGLN*, *CNN1*, and *MYH11*.

Single-cell RNA sequencing (scRNA-seq)

scRNA-seq data were obtained from the GEO database under accession number GSE212837. The processed h5ad file was downloaded and converted to Seurat v5 object format using the zellkonverter package. Hepatic stellate cells (HSCs) were extracted from the complete dataset based on the predicted cell type annotation and subjected to standard preprocessing workflow including LogNormalize normalization, identification of 2,000 highly variable features using variance stabilizing transformation (VST), and scaling of all genes. Principal component analysis (PCA) was performed and the top 30 principal components were selected based on elbow plot analysis.

To account for donor-to-donor variability, batch effect correction was performed using the Harmony algorithm (v0.1.1) with donor identity as the grouping variable for 20 iterations. Following Harmony correction, unsupervised clustering was performed using FindNeighbors and FindClusters functions with a resolution of 0.08. UMAP dimensionality reduction was computed based on the Harmony-corrected embeddings. Differential gene expression analysis was performed using the Wilcoxon rank-sum test, requiring genes to be expressed in at least 10% of cells (min.pct = 0.1) with a minimum log2 fold change of 0.25. Cluster-specific marker genes were identified using FindAllMarkers, retaining only upregulated genes (only. pos = TRUE).

Gene ontology (GO) and pathway enrichment analyses were conducted using cluster Profiler (v4.10.1). Gene symbols were converted to Entrez IDs using org.Hs.eg.db (v3.18.0). Significantly upregulated genes (adjusted p-value < 0.05 and log2 fold change > 0.25) were subjected to GO enrichment analysis for biological processes (BP) and KEGG Pathways related to cytoskeleton organization and HSC activation were identified by filtering results using keywords including "actin," "cytoskeleton," "myosin," "contractile," "focal adhesion," "collagen," "extracellular matrix," "fibroblast," and "wound healing." The Benjamini-Hochberg method was used for multiple testing correction with adjusted p-value < 0.05 as significance threshold. Expression patterns were visualized using feature plots, violin plots, dot plots, and heatmaps. All analyses were performed in R (version 4.3.1) with ggplot2 (v3.5.2) for visualization.

Biochemical analysis of serum samples

The ALT and AST levels were measured by the corresponding kits (catalog no. C009-3-1 or C010-3-1 Nanjing Jiancheng Bioengineering Institute, Nanjing, China) following the manufacturer’s instructions and detected by a Multiskan FC microplate reader (Thermo Fisher Scientific, Waltham, MA, USA).

Total RNA isolation and reverse transcription quantitative PCR (RT-qPCR)

Total RNA was extracted from liver tissues or cell lysates using Total RNA Extraction Reagent (catalog no. R401-01-AA, Vazyme, Nanjing, China). Reverse transcription of RNA into single-stranded cDNA was performed using the High-Capacity cDNA Reverse Transcription Kit (catalog no. 2816898, Thermo Fisher Scientific, Waltham, MA, USA). RT-qPCR was conducted using ChamQ Universal SYBR qPCR Master Mix (catalog no. 7E751K3, Vazyme, Nanjing, China) on a QuantStudio 5 Instrument (Thermo Fisher Scientific, Waltham, MA, USA). The mRNA levels of 18s rRNA served as an internal control for normalization. Relative mRNA expression was calculated using the $2^{-\Delta\Delta Ct}$ method. The primers used for RT-qPCR are listed in Supplementary Table S1.

Bulk RNA sequencing

RNA high-throughput sequencing was undertaken by Shanghai Majorbio Bio-pharm Biotechnology Co., Ltd. (Shanghai, China). Briefly, total RNA was extracted from the tissue using TRIzol® Reagent and transcripted into double-stranded cDNA using a SuperScript double-stranded cDNA synthesis kit (Invitrogen, CA, USA) according to the manufacturer's instructions. RNA libraries were constructed by Illumina® Stranded mRNA Prep, Ligation from Illumina (San Diego, CA, USA) using 1 μg of total RNA. Libraries were size-selected for cDNA target fragments of 300 bp using Phusion DNA polymerase. After quantification, the paired-end RNA-seq library was sequenced using the NovaSeq Xplus sequencer. The initial paired-end reads were shaped and quality controlled and resulting clean reads were aligned to the reference genome by HISAT2 software. Bulk RNA-seq data were analyzed in R (v4.3.1). Lowly expressed genes (defined as $\geqslant$10 raw counts in $\geqslant$3 samples) were filtered prior to analysis. Differential expression analysis was performed using DESeq2 (v1.38.3), and genes with adjusted $P < 0.05$ (Benjamini–Hochberg) and $|\log_2$ fold change$| > 0.5$ were considered significant. Results were visualized using volcano plots and heatmaps based on $\log_2$(TPM+1)–scaled expression of selected cytoskeleton- and matrix-related genes. Gene Set Enrichment Analysis (GSEA) was performed using the pre-ranked $\log_2$ fold-change list with Hallmark mouse-ortholog gene sets from MSigDB in the GSEA software (v4.1.0), and enrichment significance was defined as adjusted $P < 0.05$. Over-representation analysis (ORA) of significantly up-regulated genes was conducted using clusterProfiler (v4.10.1), with GO (BP, MF, CC), KEGG, and Reactome pathway databases queried; gene IDs were mapped using org.Mm.eg.db (v3.18.0). Enrichment results were visualized using dot plots, GSEA running enrichment plots, and category heatmaps to highlight pathways associated with actin filament organization, actin binding,

cytoskeleton and extracellular matrix. Raw data have been uploaded to NCBI Sequence Read Archive (SRA) database with the accession number: PRJNA1472899.

Western blot

Liver tissues and cell samples were lysed with RIPA buffer (catalog no. YH374135, Thermo Fisher Scientific, Waltham, MA, USA) supplemented with Halt Protease and Phosphatase Inhibitors (catalog no. 78447, Thermo Fisher Scientific, Waltham, MA, USA). Protein samples were loaded on the polyacrylamide gels (catalog no. 220A019, Absin, Shanghai, China) and transferred onto nitrocellulose membranes (catalog no. 0000208128, Merck, Darmstadt, Germany). The membranes were blocked with 1% bovine serum albumin (BSA) and incubated with primary antibodies at 4°C overnight. The following antibodies were used: ITGBL1 (catalog no. NBQ1-82473, Novus Biologicals, Centennial, CO, USA), Desmin (catalog no. GB12075-100, Servicebio, Wuhan, China), MYH9 (catalog no. PA5-29673, Thermo Fisher Scientific, Waltham, MA, USA), Phospho-MYH9 (catalog no. AF4425, Affinity Biosciences, Cincinnati, OH, USA), Col1α1 (catalog no. ab260043, Abcam, Cambridge, UK), Col3α1 (catalog no. A0817, ABclonal, Wuhan, China), FAK (catalog no. 3285, Cell Signaling Technology, Danvers, MA, USA), α-SMA (catalog no. 19245S,Cell Signaling Technology, Danvers, MA, USA), Phospho-FAK (Tyr397) (catalog no. 3283, Cell Signaling Technology, Danvers, MA, USA), DYKDDDDK Tag (catalog no. 14793, Cell Signaling Technology, Danvers, MA, USA), DYKDDDDK Tag (catalog no. 8146S, Cell Signaling Technology, Danvers, MA, USA), DYKDDDDK Tag (catalog no. AE005, ABclonal, Wuhan, China), β-actin (catalog no. AC026, ABclonal, Wuhan, China).

After washing with Tris-buffered saline containing 0.1% Tween 20 (TBST), the membranes were incubated with HRP-conjugated anti-rabbit or anti-mouse IgG secondary antibodies (catalog no. 7074S and 7076S, Cell Signaling Technology, Danvers, MA, USA). Protein bands were visualized using the SuperSignal Maximum Sensitivity Substrate (catalog no. WG328673, Thermo Fisher Scientific, Waltham, MA, USA)

Tissue processing, histological analysis and immunohistochemistry staining

Liver tissues were fixed with 10% formaldehyde, embedded in paraffin, sectioned into 5-μm-thick slices, dehydrated with ethanol and xylol and washed briefly. Hematoxylin and eosin (H&E) staining for histological analysis was performed by using H&E stain solution (catalog no. G1004, Servicebio, Wuhan, China) according to the manufacturer’s instructions. For Sirius red staining, the paraffin-embedded liver sections were dewaxed in xylene and then stained with PICRO-RED STAINING SOLUTION (catalog no. 20230310,

Phygene, Fuzhou, China) for 1 hour according to the manufacturer's instructions. For immunohistochemistry staining, liver sections were subjected to antigen retrieval with citrate buffer (catalog no. 005000, Invitrogen, CA, USA). After incubating with 3% hydrogen peroxide (H_2O_2), the sections were blocked with 3% normal goat serum (NGS) buffer and then incubated with primary antibodies overnight at 4°C. The following antibodies were used: ITGBL1 (catalog no. NBQ1-82473, Novus Biologicals, Centennial, CO, USA), MYH9 (catalog no. PA5-29673, Thermo Fisher Scientific, Waltham, MA, USA), Col1α1 (catalog no. ab260043, Abcam, Cambridge, UK), α-SMA (catalog no. 19245S, Cell Signaling Technology, Danvers, MA, USA), F4/80 (catalog no. 70076, Cell Signaling Technology, Danvers, MA, USA). Finally, the sections were incubated with secondary antibodies (catalog no. 8814S or 8125S, Cell Signaling Technology, Danvers, MA, USA) at room temperature for 1 hour. ImmPACT DAB Substrate Kit (catalog no. ZK1018, Vector 2 Laboratories, CA, USA) was used to visualize the staining.

Immunofluorescence staining

Liver sections were subjected to antigen retrieval with citrate buffer (catalog no. 005000, Invitrogen, CA, USA) and cell samples were fixed with 4% paraformaldehyde (PFA). After incubating at 3% NGS, sections were incubated with primary antibodies overnight at 4°C and incubated with secondary antibodies (catalog no. 4412S or 4413S, Alexa Fluor 488 or 555 goat anti-rabbit IgG [H+L], Cell Signaling Technology; catalog no. 4408S or 4409S, Alexa Fluor 488 or 555 goat anti-mouse IgG [H+L], Cell Signaling Technology; catalog no. 4417S Alexa Fluor 555 goat anti-Rat IgG [H+L], Cell Signaling Technology, Danvers, MA, USA; catalog no. A10525, Goat anti-Rat IgG (H+L) Cross-Adsorbed Secondary Antibody, Cyanine5, Thermo Fisher Scientific, Waltham, MA, USA) for 1 hour at room temperature. The TrueView Autofluorescence Quenching Kit (catalog no. ZK0818, Vector Laboratories, CA, USA) was used to remove non-specific fluorescence. Nuclear staining was conducted with 4', 6'-diamino-2-phenylindole (DAPI) (catalog no. P0131, Beyotime, Shanghai, China). The following antibodies were used: ITGBL1 (catalog no. NBQ1-82473, Novus Biologicals, Centennial, CO, USA), Desmin (catalog no. GB12075-100, Servicebio, Wuhan, China), MYH9 (catalog no. PA5-29673, Thermo Fisher Scientific, Waltham, MA, USA), ,Phospho-FAK (Tyr397) (catalog no. 3283, Cell Signaling Technology, Danvers, MA, USA), Alexa Fluor 555 Phalloidin (catalog no. 8953, Cell Signaling Technology, , Danvers, MA, USA).

Multiple immunofluorescence staining

Multiplex immunofluorescence staining based on tyramide signal amplification (TSA) was performed using a

commercial TSA Fluorescence Triple Staining Plus Kit (ABclonal, catalog RK50026P), according to the manufacturer's instructions. Briefly, paraffin-embedded liver sections (3–5 μm) were baked at 65 °C for 1 h, followed by deparaffinization in xylene (2 × 15 min) and rehydration through graded ethanol solutions. Antigen retrieval was performed in citrate buffer (pH 6.0) using a water bath for 10–20 min. After cooling to room temperature, endogenous peroxidase activity was quenched with 3% H_2O_2 for 10 min. Sections were washed in PBS and incubated with blocking buffer for 30 min to reduce non-specific binding. For sequential TSA staining, target antigens were detected sequentially. For each staining round, sections were incubated with the primary antibody at the recommended IHC dilution, followed by HRP-conjugated secondary antibody (50 min at room temperature). After washing, tyramide working solution (1:200) was applied for 10 min to catalyze fluorophore deposition. Slides were rinsed thoroughly to remove unbound substrates. Between cycles, HRP signals and antibody complexes were removed by heat-induced epitope retrieval (same buffer and conditions as above) to allow the next round of staining. The above steps were repeated for each target using spectrally separated fluorophores (520 nm, 570 nm, 620 nm channels), proceeding from low-abundance to high-abundance targets to avoid signal masking. After completion of all staining cycles, nuclei were counterstained with DAPI for 5 min. Slides were mounted using an anti-fade mounting medium and stored at 4 °C in darkness. The sections were imaged using an Olympus BX43 microscope (Olympus, Tokyo, Japan). The following antibodies were used: ITGBL1 (catalog no. NBQ1-82473, Novus Biologicals, Centennial, CO, USA), MYH9 (catalog no. PA5-29673, Thermo Fisher Scientific, Waltham, MA, USA), Desmin (catalog no. ab15200, Abcam, Cambridge, UK).

Isolation of primary mouse HSCs

Primary mouse HSCs were isolated by collagenase digestion followed by centrifugation in an OptiPrep gradient, as previously described (1). Briefly, after the mice were anesthetized with pentobarbital, the livers were sequentially perfused with Ethylene glycol tetraacetic acid (EGTA), pronase solutions (catalog no. P5147, Merck, Darmstadt, Germany), and collagenase solutions (catalog no. 41J21479, Worthington, CO, USA). After in vitro digestion for 30 minutes, hepatocytes were separated by centrifugation at 400 rpm for 5 minutes, and the supernatant was centrifuged at 1600 rpm for 10 minutes to collect the precipitate. After centrifugation, the cell pellet was suspended in 20% OptiPrep (catalog no. D1556, Merck, Darmstadt, Germany) and carefully added 11.5% OptiPrep and Hank's Balanced Salt Solution (HBSS) respectively on it. After centrifugation at 3000 rpm for 17 minutes at 4°C, the cell fraction between 11.5% OptiPrep and HBSS solution was collected gently and washed with HBSS solution. Finally, the isolated HSCs were cultured in

RPMI 1640 containing 10% fetal bovine serum (FBS) overnight, and the medium was changed every day.

Cell culture and transfection of lentivirus

Human HSC cell line LX-2 was kindly provided by Prof. Jing Chen, Shanghai Institute of Materia Medica (SIMM), Chinese Academy of Sciences. Primary HSCs and LX2 cells were cultured in RPMI 1640 medium (catalog no. 2786824, Gibco, NY, USA) containing 10% FBS (catalog no. 2500251P, Gibco, NY, USA), and penicillin-streptomycin (catalog no. 15140-122, Gibco, NY, USA). For lentivirus-ITGBL1 (Lv-ITGBL1) (MOI=3, Hanbio, Shanghai, China) infection, when the primary HSCs or LX2 cells were at 60% confluence, the culture medium was removed before infection. The cells were washed gently with PBS and treated with the virus-containing medium (MOI of approximately 3) combined with Polybrene (5 μg/ml). After 24 hours, culture medium was removed and fresh medium was added. The empty lentivector Lv-EGFP (Lv-control) was used as negative control (Hanbio, Shanghai, China). 48 hours after viral infection, primary HSCs or LX2 cells were harvested or treated with TGF-β1 (5 ng/ml) and examined for the related gene expression by western blot and RT-qPCR.

Co-Immunoprecipitation (Co-IP) and Proteomics

For denaturing immunoprecipitation, the lysates were prepared by using Pierce™ Classic Magnetic IP/Co-IP Kit (catalog no. 88804, Thermo Fisher Scientific, Waltham, MA, USA) according to the manufacturer's protocol and immunoprecipitated with anti-Flag (catalog no. 8146, Cell Signaling Technology, Danvers, MA, USA) antibody. Proteomic analysis was performed by PTM-Bio (Hangzhou, China).

For in-gel tryptic digestion, excised gel pieces were destained in 50 mM NH_4HCO_3 containing 50% acetonitrile (v/v) until fully clear. The gel pieces were dehydrated with 100% acetonitrile for 5 min, the solvent was removed, and the pieces were rehydrated in 10 mM dithiothreitol and incubated at 56 °C for 60 min. After a second dehydration step with 100% acetonitrile, the gel pieces were rehydrated in 55 mM iodoacetamide and incubated at room temperature in the dark for 45 mins. The pieces were then washed with 50 mM NH_4HCO_3 and dehydrated again with 100% acetonitrile. For digestion, gel pieces were rehydrated on ice with trypsin (10 ng/μL in 50 mM NH_4HCO_3) for 1 h, excess solution was removed, and samples were incubated at 37 °C overnight. Peptides were sequentially extracted using 50% acetonitrile/5% formic acid followed by 100% acetonitrile, dried completely, and reconstituted in 2% acetonitrile/0.1% formic acid.

Tryptic peptides were dissolved in 0.1% formic acid (solvent A) and loaded onto a homemade reversed-phase analytical column (15 cm length, 75 μm inner diameter). Peptides were separated on an EASY-nLC 1000 UPLC system using a linear gradient from 6% to 23% solvent B (0.1% formic acid in 98% acetonitrile) over 16 min, followed by an increase to 35% over 8 min and then to 80% over 3 min, where it was held for an additional 3 min. The flow rate was maintained at 400 nL/min throughout the run.

Eluted peptides were ionized using a nanospray ionization (NSI) source and analyzed on a Q Exactive™ Plus mass spectrometer (Thermo Fisher Scientific) operating in data-dependent acquisition mode. The spray voltage was set to 2.0 kV. Full MS scans were acquired in the Orbitrap at a resolution of 70,000 over an m/z range of 350–1800. The top 20 most intense precursor ions were selected for fragmentation using higher-energy collisional dissociation (HCD) with a normalized collision energy (NCE) of 28, and MS/MS spectra were acquired at a resolution of 17,500. Dynamic exclusion was set to 15 s to prevent repeated sequencing of the same precursor ion. The automatic gain control (AGC) target value was set to 5×10^4.

Raw MS/MS data were analyzed using Proteome Discoverer (version 2.4). Spectra were searched against the appropriate UniProt Homo sapiens reference database using the following parameters: Trypsin/P specificity with up to two missed cleavages; precursor ion mass tolerance of 10 ppm; fragment ion tolerance of 0.02 Da. Carbamidomethylation of cysteine was set as a fixed modification, whereas methionine oxidation was included as a variable modification. Peptide identifications were filtered using a high-confidence threshold, with peptide ion scores >20 retained for downstream analysis.0.

Cell Contraction Assay

LX-2 cells were transduced with Lv-*ITGBL1* or *MYH9* siRNA for 48h before the contraction assay. Cells were subsequently harvested by trypsinization and resuspended in complete medium at a density of 1×10^5 cells/ml. Collagen gels were prepared according to the manufacturer’s instructions for the collagen gel contraction assay kit (catalog no. CBA-201, Cell Biolabs, San Diego, CA, USA). Briefly, the cell suspension was mixed with the Collagen Gel Working Solution at a 1:4 ratio, and 500 μL of the mixture was added to each well of a 24-well plate. The plate was incubated at 37°C for 1 h to allow complete polymerization. After gelation, collagen gels were gently released from the well edges using a sterile spatula, and 1 mL of complete medium was added to each well. Gels were imaged at the indicated time points. Gel area was quantified using ImageJ, and contraction was analyzed based on the reduction of gel area over time.

References:

1. Mederacke I, Dapito DH, Affo S, Uchinami H, and Schwabe RF. High-yield and high-purity isolation of hepatic stellate cells from normal and fibrotic mouse livers. *Nat Protoc.* 2015;10(2):305-15.

Supplementary Table 1: The sequence of used mRNA primers

Genes (mouse)	Forward primer (5'→3')	Reverse primer (5'→3')
18s	AACTTTCGATGGTAGTCGCCGT	TCCTTGGATGTGGTAGCCGTTT
Acta2	TCCTGACGCTGAAGTATCCGATA	GGTGCCAGATCTTTTCCATGTC
Col1a1	TAGGCCATTGTGTATGCAGC	ACATGTTCAGCTTTGTGGACC
Col1a2	GGTGAGCCTGGTCAAACGG	ACTGTGTCCTTTCACGCCTTT
Col3a1	TAGGACTGACCAAGGTGGCT	GGAACCTGGTTTCTTCTCACC
Col4a1	CACATTTTCCACAGCCAGAG	GTCTGGCTTCTGCTGCTCTT
Fn1	TTCAAGTGTGATCCCCATGAAG	CAGGTCTACGGCAGTTGTCA
Vimentin	TCCACACGCACCTACAGTCT	CCGAGGACCGGGTCACATA
Itgbl1	GTGAGTGCCATGAGTGGATATG	CTTGCCGCAGTCACAATTACC
F4/80	GGAAAGCACCATGTTAGCTGC	CCTCTGGCTGCCAAGTTAATG
Il1b	TCGCTCAGGGTCACAAGAAA	CATCAGAGGCAAGGAGGAAAAC
Tnfa	AGGCTGCCCCGACTACGT	GACTTTCTCCTGGTATGAGATAGCAAA
Srebp1	GCAGCCACCATCTAGCCTG	CAGCAGTGAGTCTGCCTTGAT
Acc1	ATGGGCGGAATGGTCTCTTTC	TGGGGACCTTGTCTTCATCAT
Myh9	TGTAAGGTGACTGTAGGAAAGAGC	ATCTTTTACCATCTGCCTCCAGTT
**Genes (Human)**	**Forward primer (5'→3')**	**Reverse primer (5'→3')**
18s	GGCCCTGTAATTGGAATGAGTC	CCAAGATCCAACTACGAGCTT
ITGBL1	AGACCTACGACGGGAGCAC	ACCTGCATTAGAGCAGATGATGT
ACTA2	GTGACGAAGCACAGAGCAAA	CTTTTCCATGTCGTCCCAGT
COL1A1	CAGATCACGTCATCGCACAA	TGTGAGGCCACGCATGAG
COL3A1	AGGACTGACCAAGATGGGAA	AGGGGAGCTGGCTACTTCTC
MYH9	CAGCAAGCTGCCGATAAGTAT	CTTGTCGGAAGGCACCCAT

Supporting Figure S1

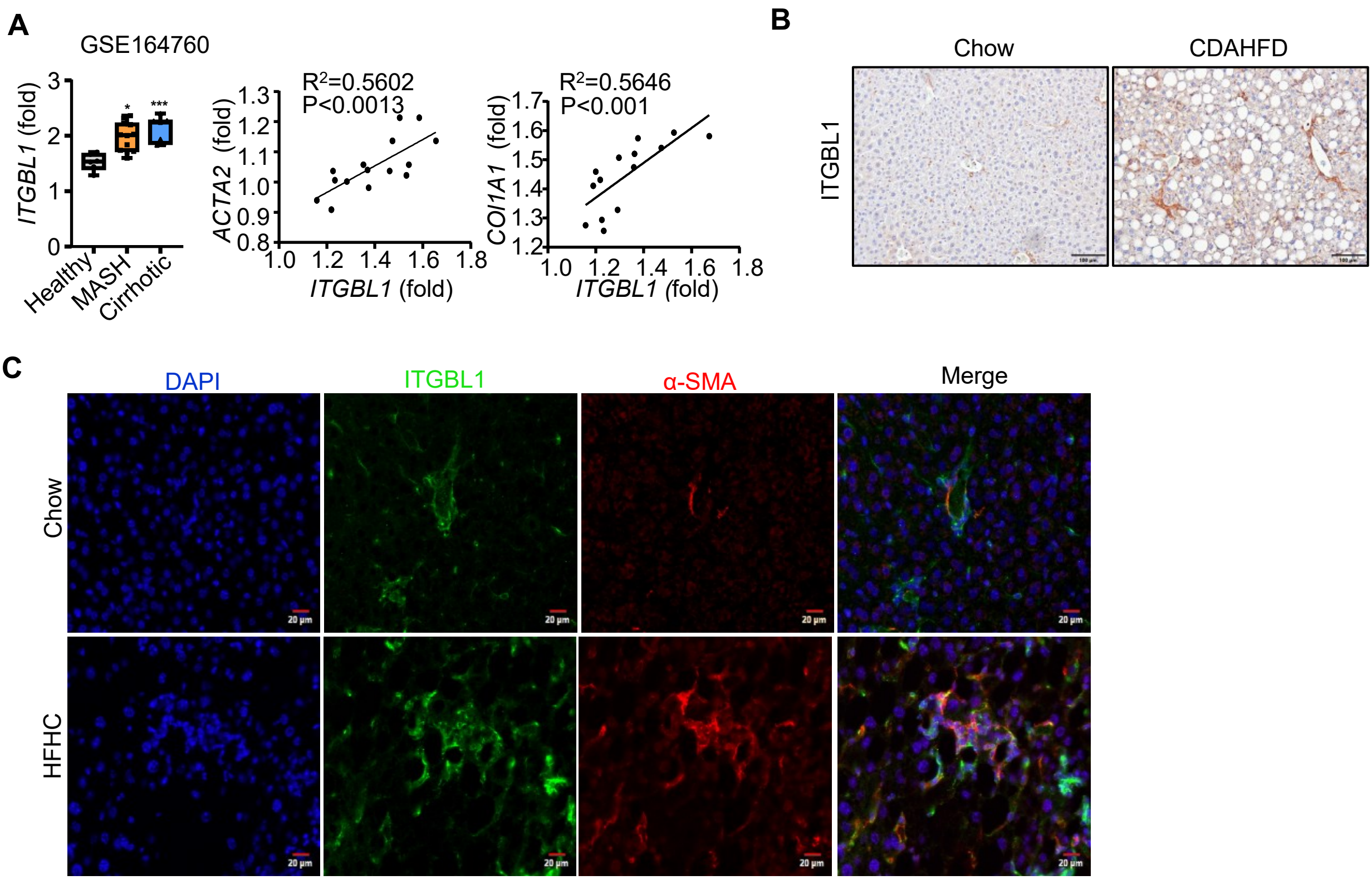

Supporting Figure S1. Hepatic ITGBL1 expression is significantly increased in MASH-related liver fibrosis. (A) The mRNA levels of *ITGBL1* in the livers of patients with MASH and cirrhosis were analyzed in a public database (GSE164760). The correlation between *ITGBL1* and *COL1A1/COL1A2 was* analyzed. **(B)** WT mice were fed a chow or CDAHFD diet for 4 weeks. Liver tissues were collected and subjected to immunohistochemistry staining. Representative images of immunohistochemistry staining of ITGBL1 are shown. **(C)** WT mice were fed a chow or HFHC diet for 16 weeks. Liver tissues were collected and subjected to immunofluorescence staining. Representative images of immunofluorescence double staining of ITGBL1 (Green) and α-SMA (Red) in HFHC-fed mice are shown. Values represent mean ± SEM. Significance was calculated using a 2-tailed Student's *t* test for comparing 2 groups (**A**). *P<0.05, ***P<0.001.

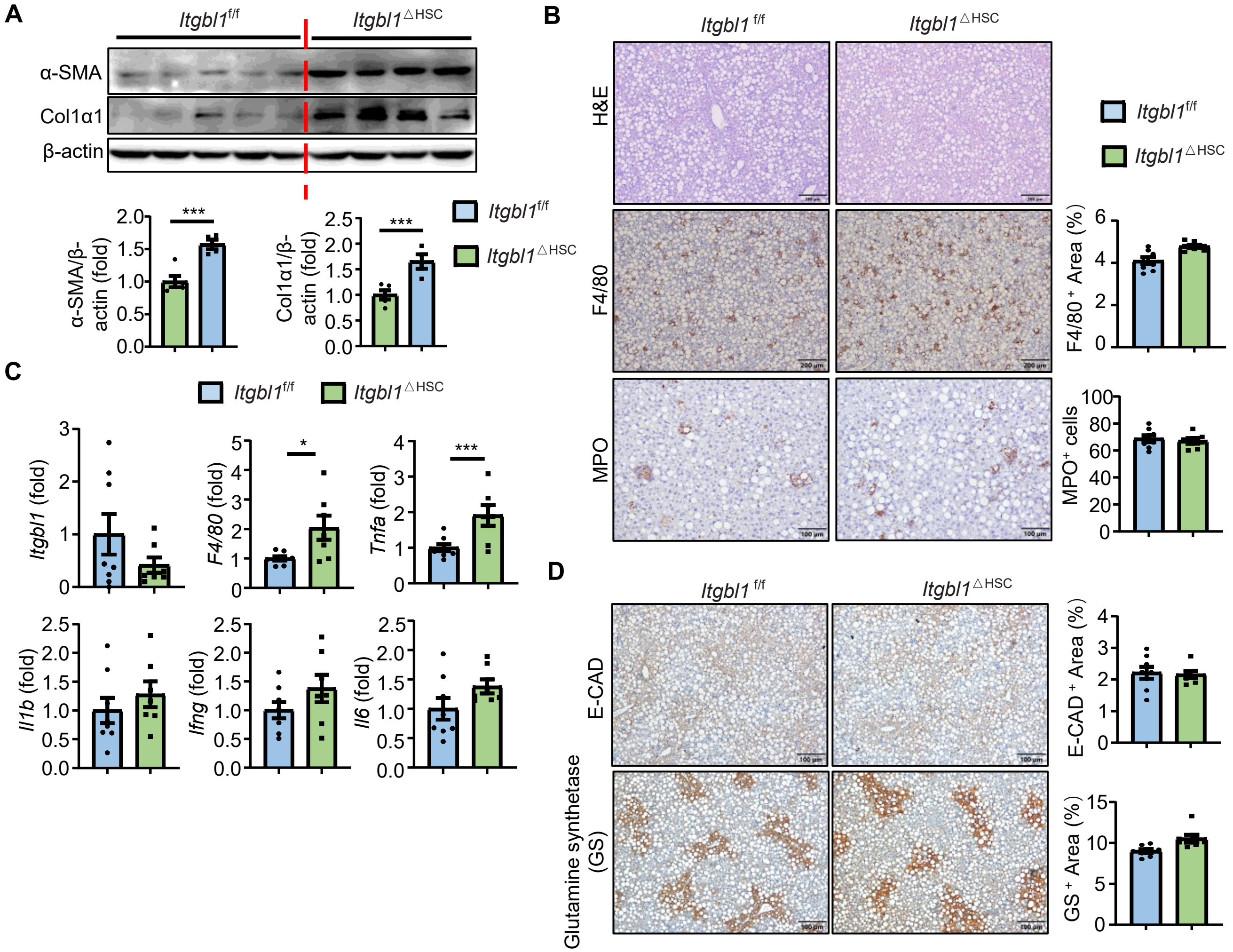

Supporting Figure S2. *Itgbl1* deletion in HSCs causes greater macrophage infiltration but does not affect hepatic steatosis and liver zonation in CDAHFD-induced MASH model. (A) *Itgbl1* $^{f/f}$ and *Itgbl1* $^{\triangle HSC}$ mice were fed a CDAHFD for 4 weeks. Liver tissues were collected and subjected to further analysis. Western blot analyses of hepatic α-SMA and Col1α1 protein expression. Relative protein expression was quantified. **(B)** Representative images of H&E staining and immunohistochemistry staining of F4/80 and MPO are shown. Positive area or cells per field were quantified. **(C)** RT-qPCR analyses of several hepatic gene levels in CDAHFD-fed *Itgbl1* $^{f/f}$ and *Itgbl1* $^{\triangle HSC}$ mice. **(D)** Representative images of immunohistochemistry staining of E-cadherin and GS are shown. Positive area per field were quantified. Values represent mean ± SEM. Significance was calculated using a 2-tailed Student's *t* test for comparing 2 groups (**A-D**). *P<0.05, ***P<0.001.

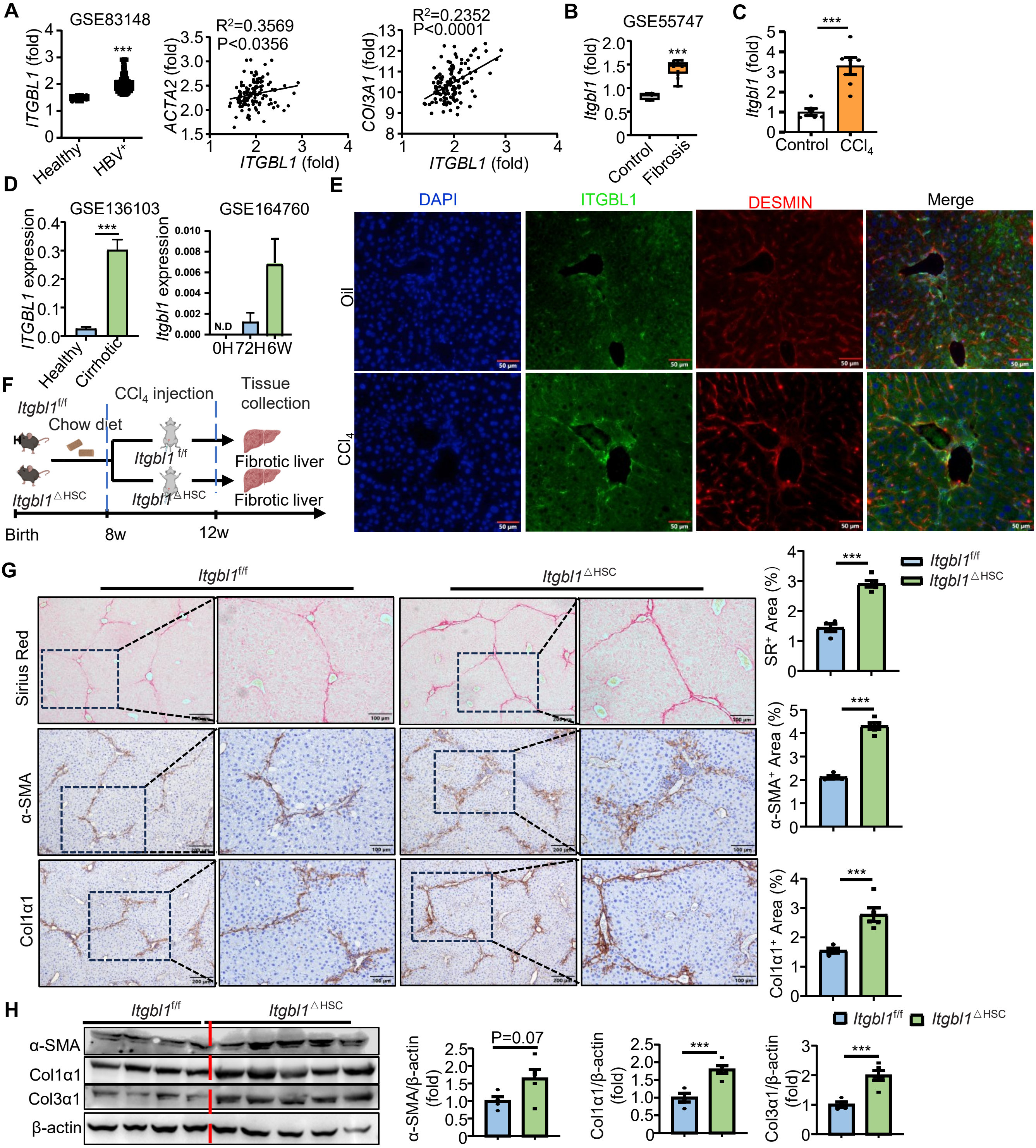

Supporting Figure S3. HSC-specific ablation of *Itgbl1* exacerbates CCl_4-induced liver fibrosis. (A) The hepatic expression of *ITGBL1* in the livers of patients with HBV. The correlation between *ITGBL1* and *ACTA2*/*COL3A1* was analyzed. **(B)** Hepatic *Itgbl1* expression in CCl_4-induced liver fibrosis was analyzed by public database. **(C)** RT-qPCR analysis of hepatic *Itgbl1* mRNA levels in CCl_4-induced liver fibrosis. **(D)** The expression of ITGBL1 in HSCs from human cirrhotic and CCl_4-treated mice was elevated by analyzing public database. **(E)** Representative images of immunofluorescence double staining of ITGBL1 (green) and Desmin (red) in CCl_4-induced liver fibrosis are shown. **(F)** Regimen of *Itgbl1*$^{f/f}$ and *Itgbl1*$^{\Delta HSC}$ mice receiving CCl_4 (10% CCl_4, 2 ml/kg) for 4 weeks. **(G)** Representative images of Sirius red staining and IHC staining of Col1α1 and α-SMA are shown. The degree of liver fibrosis was quantified. (**H**) Western blot analyses of protein levels of α-SMA, Col1α1 and Col3α1. Relative protein levels were quantified. Values represent mean ± SEM. Significance was calculated using a 2-tailed Student's *t* test for comparing 2 groups (**A-D**, **G**, and **H**). *P<0.05, ***P<0.001.

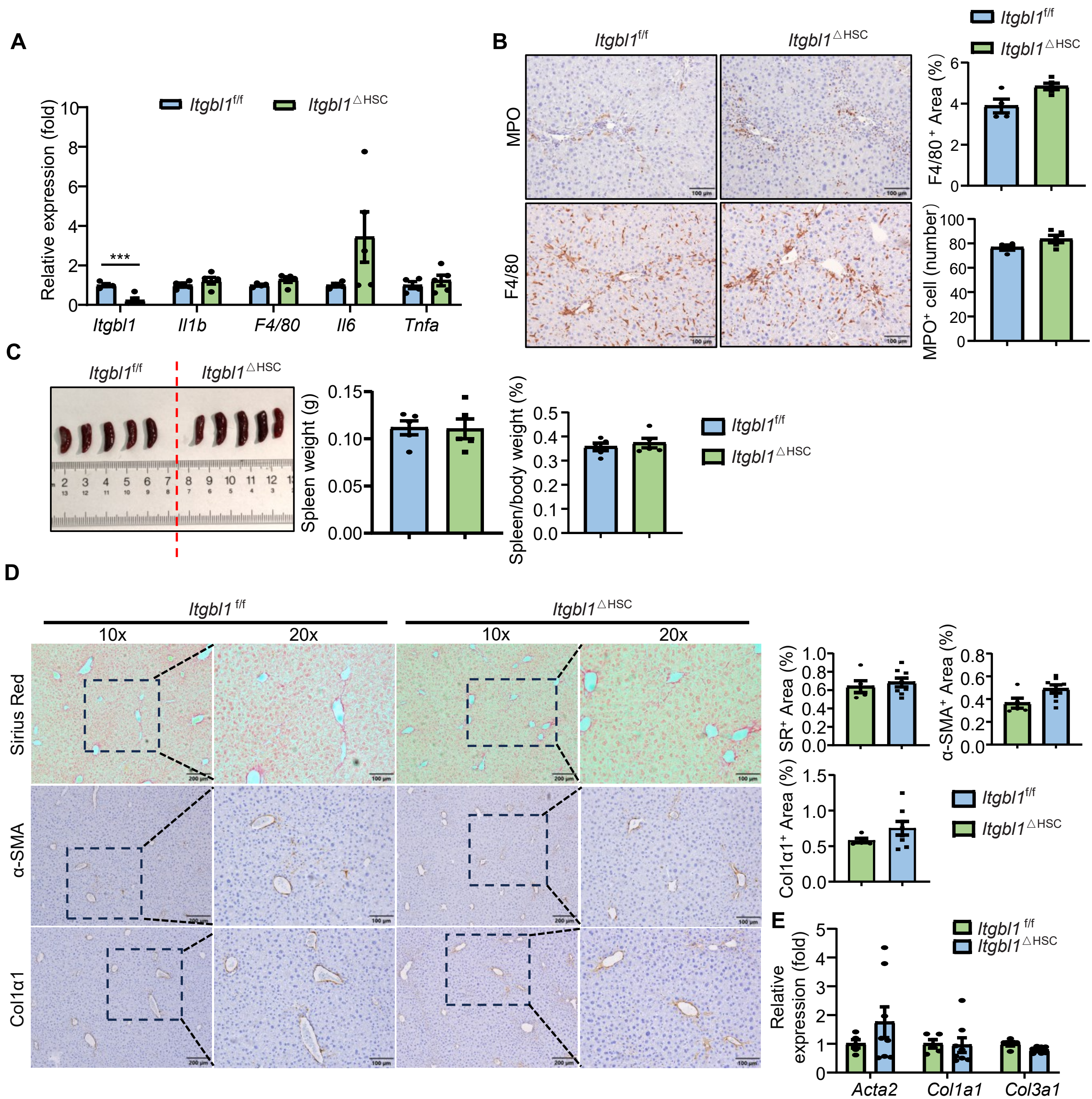

Supporting Figure S4. Loss of *Itgbl1* in HSCs does not cause spontaneous liver fibrosis in mice. (A) RT-qPCR analyses of several hepatic gene levels in CCl_4-treated *Itgbl1*$^{f/f}$ and *Itgbl1*$^{\triangle HSC}$ mice. **(B)** Representative images of immunohistochemistry staining of MPO and F4/80 are shown. **(C)** Images of spleens from CCl_4-treated *Itgbl1*$^{f/f}$ and *Itgbl1*$^{\triangle HSC}$ mice. Spleen weight and spleen-to-body weight ratio were measured. **(D)** *Itgbl1*$^{f/f}$ and *Itgbl1*$^{\triangle HSC}$ mice were fed a chow diet for one year. Representative images of Sirius Red, α-SMA and Col1α1 staining are shown. Positive area per field were quantified. **(E)** RT-qPCR analysis of hepatic fibrosis-related genes in *Itgbl1*$^{f/f}$ and *Itgbl1*$^{\triangle HSC}$ mice. Values represent mean ± SEM. Significance was calculated using a 2-tailed Student's *t* test for comparing 2 groups (**A** , **C** , and **D**). ****P*< 0.001.

Supporting Figure S5

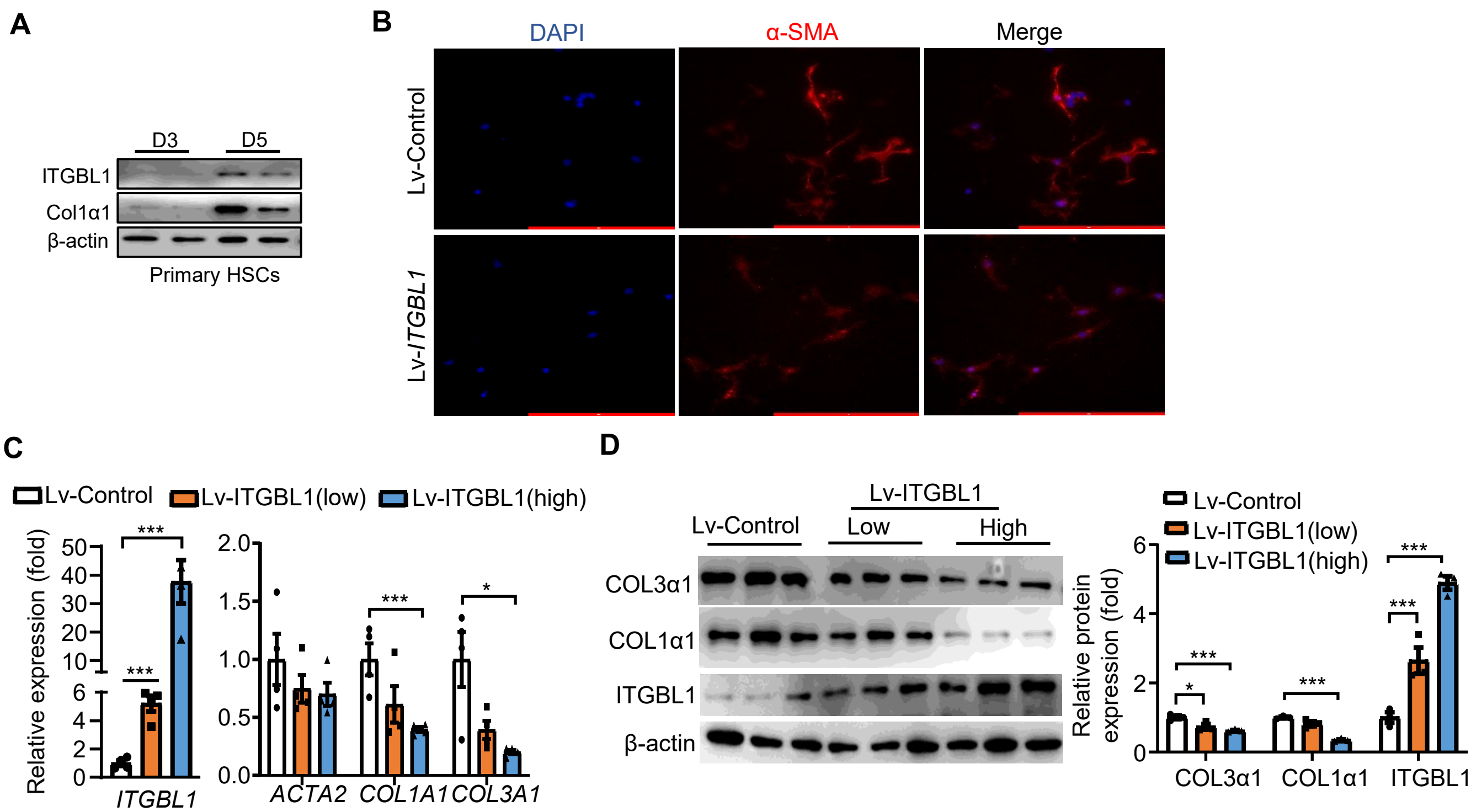

Supporting Figure S5. ITGBL1 overexpression inhibits the formation of F-actin stress fibers, thus limiting HSC activation. (A) Primary mouse HSCs were isolated and subjected to Western blot. **(B)** Primary HSCs were isolated and infected with lentivirus-*Itgbl1* (Lv-*Itgbl1*) for 48 hours. Cells were then collected. Representative images of immunofluorescence staining of α-SMA (red) are shown. Scale bar, 50 μm. **(C)** RT-qPCR analyses of several gene levels in Control, low-dose ITGBL1 overexpression, and high-dose ITGBL1 overexpression in LX2 cells. **(D)** Western blot analyses of protein levels of ITGBL1, Col3α1, Col1α1 and β-actin. Relative protein expression was quantified. Values represent mean ± SEM. Significance was calculated using a 1-way ANOVA for multiple groups (**C** and **D**). ***P<0.001.

Supporting Figure S6

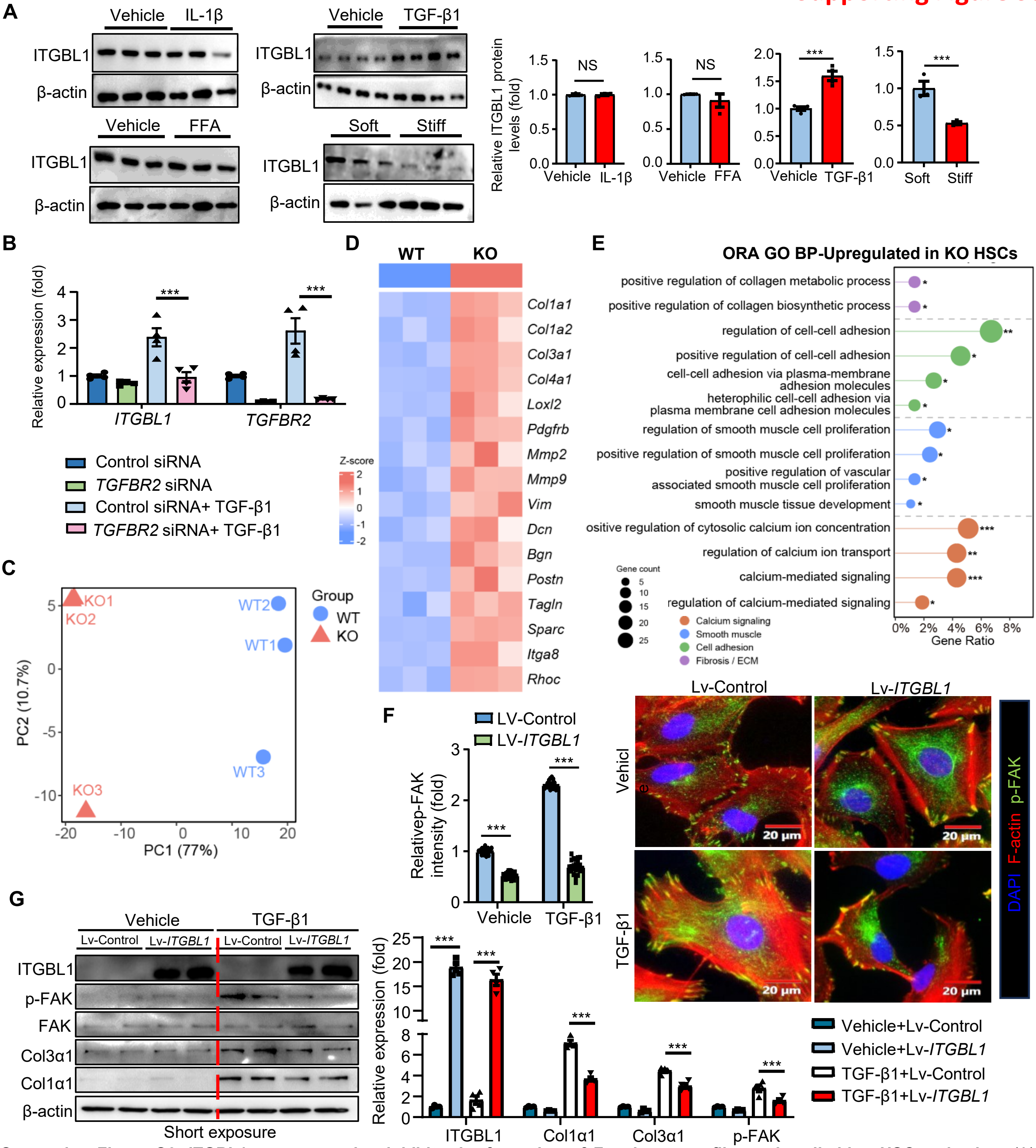

Supporting Figure S6. ITGBL1 overexpression inhibits the formation of F-actin stress fibers, thus limiting HSC activation. (A) Protein expression of ITGBL1 in HSCs under IL-1β, TGF-β1, FFA, soft matrix, and stiff matrix conditions was determined by Western blotting. Relative protein expression was quantified. **(B)** RT–qPCR analyses of *ITGBL1* and *TGFBR2* expression in control siRNA and TGFBR2 siRNA-transfected LX-2 cells with or without TGF-β1 stimulation. **(C-E)** Primary HSCs were isolated from CDAHFD-fed *Itgbl1*$^{f/f}$ (WT) and *Itgbl1*$^{\Delta HSC}$ (KO) mice and subjected to RNA-seq analysis. Principal component analysis (PCA) was performed (C). Heatmap showed the expression of multiple fibrosis- and cytoskeleton-associated genes (D). Pathway enrichment analysis was performed (E). **(F)** LX-2 cells were infected with Lv-*ITGBL1* for 48 hours and stimulated with TGF-β1 (5 ng/ml) for 48 hours. Representative images of double immunofluorescence staining of p-FAK (green) and F-actin (red) are shown. Relative p-FAK intensity was quantified. **(G)** LX-2 cells were infected with Lv-*ITGBL1* for 48 hours and stimulated with TGF-β1 (5 ng/ml) for 24 hours. Protein levels of ITGBL1, p-FAK, FAK, Col3α1, Col1α1 and β-actin were measured by western blotting. Relative protein expression was quantified. Values represent mean ± SEM. Significance was calculated using a 1-way ANOVA for multiple groups (**B**, **F** and **G**) and a 2-tailed Student's *t* test for comparing 2 groups (**A**). ***P<0.001.

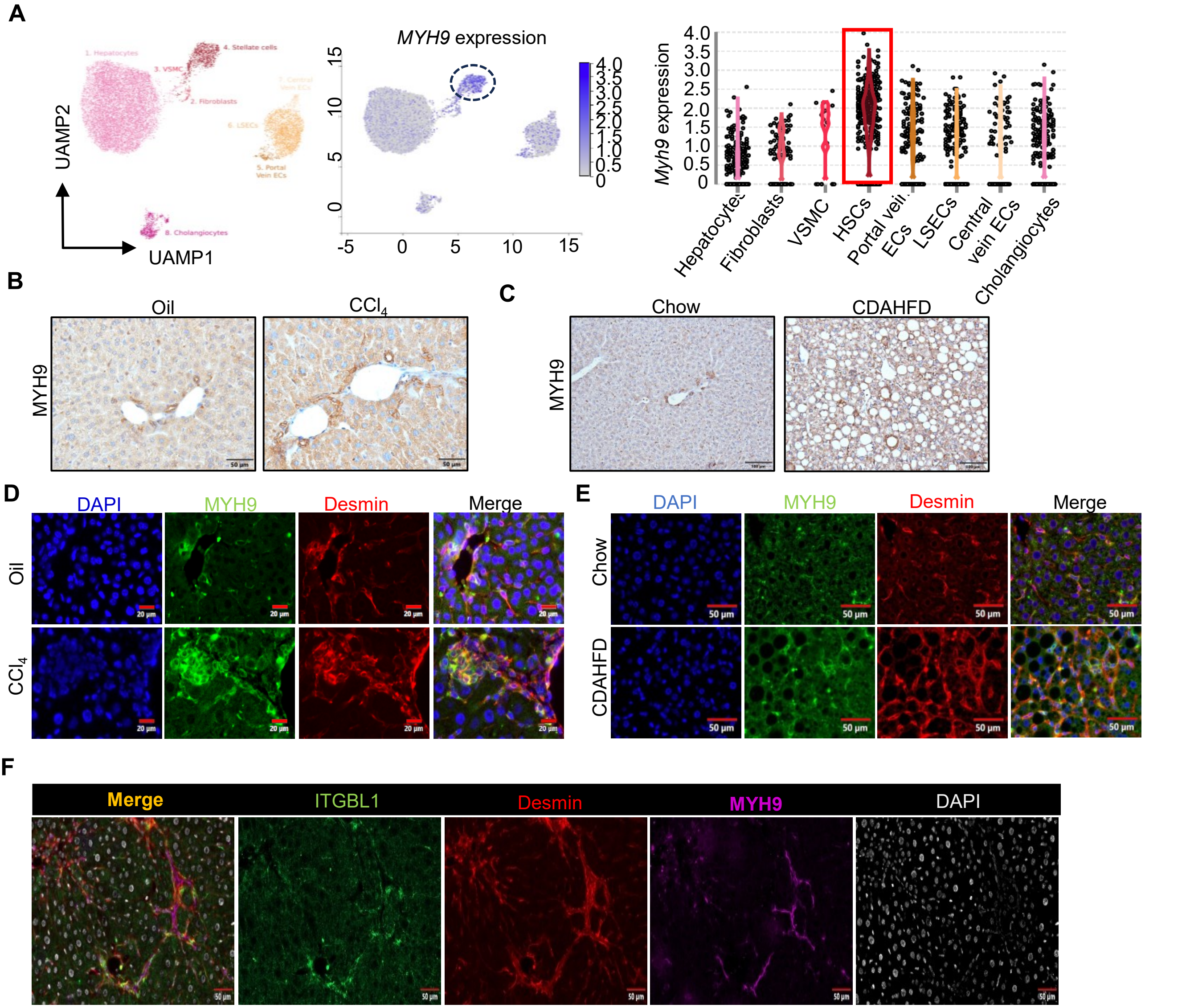

Supporting Figure S7. MYH9 is mainly expressed in HSCs and elevated in liver fibrosis. (A) Hepatic *MYH9* expression was analyzed by scRNA-seq from Liver Cell Atlas database (https://www.livercellatlas.org/index.php). **(B)** WT mice were injected with CCl_4 for 6 weeks. Liver samples were collected. Representative images of immunohistochemical staining of MYH9 are shown. **(C)** Representative images of immunohistochemical staining of MYH9 in CDAHFD-fed WT mice are shown. **(D)** Representative images of double immunofluorescence staining of MYH9 (green) and Desmin (Red) in CCl_4-induced fibrosis mice are shown. Scale bar, 100 μm. **(E)** Representative images of double immunofluorescence staining of MYH9 (green) and Desmin (Red) in CDAHFD-induced fibrosis are shown. Scale bar, 50 μm. **(F)** Representative images of immunofluorescence staining of ITGBL1 (green), MYH9 (pink) and Desmin (red) in CCl_4-treated mice are shown.

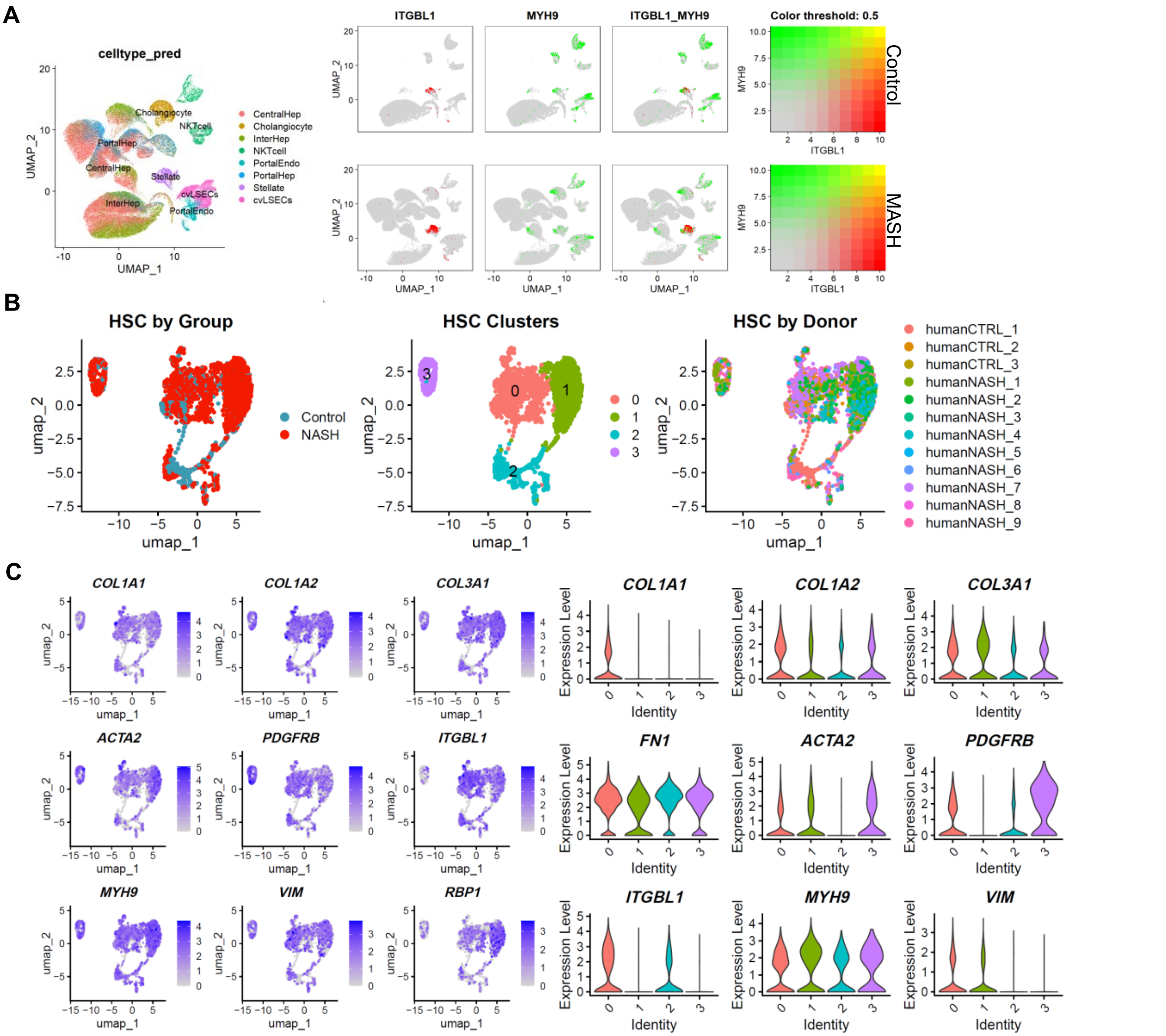

Supporting Figure S8. ITGBL1 positively correlates with MYH9 in a distinct subset of HSCs in patients with MASH. (A) UMAP visualization displays cell-type annotation of the scRNA-seq dataset (GSE212837) from human liver samples. The left panel shows the distribution of major cell populations including central hepatocyte (CentralHep), cholangiocyte, intermediate hepatocyte (InterHep), natural killer T cells (NKTcell), portal endothelial cell (PortalEndo), portal hepatocyte (PortalHep), hepatic stellate cell (Stellate), central vein liver sinusoidal endothelial cells (cvLSECs). The right panels demonstrate the co-expression analysis of *ITGBL1* and *MYH9* across different cell types in control (upper panels) and MASH (lower panels) samples. Individual *ITGBL1* expression is shown in the first column, *MYH9* expression in the second column, and co-expression in the third column, where yellow indicates high co-expression of both genes. The color gradient scales indicate normalized expression levels. (B) UMAP plots show HSC populations extracted from the integrated dataset. The left panel displays HSCs grouped by disease condition (Control in blue and MASH in red). The middle panel shows four distinct HSC subclusters (Clusters 0-3) identified through unsupervised clustering analysis. The right panel illustrates HSC distribution colored by individual donors, including 3 control and 9 MASH samples. (C) Feature and violin plots show the expression level and distribution of HSC marker genes and fibrosis-related genes across the four HSC subclusters (Clusters 0-3). Each violin plot represents the expression density distribution within the respective cluster, with wider sections indicating higher cell density at that expression level.

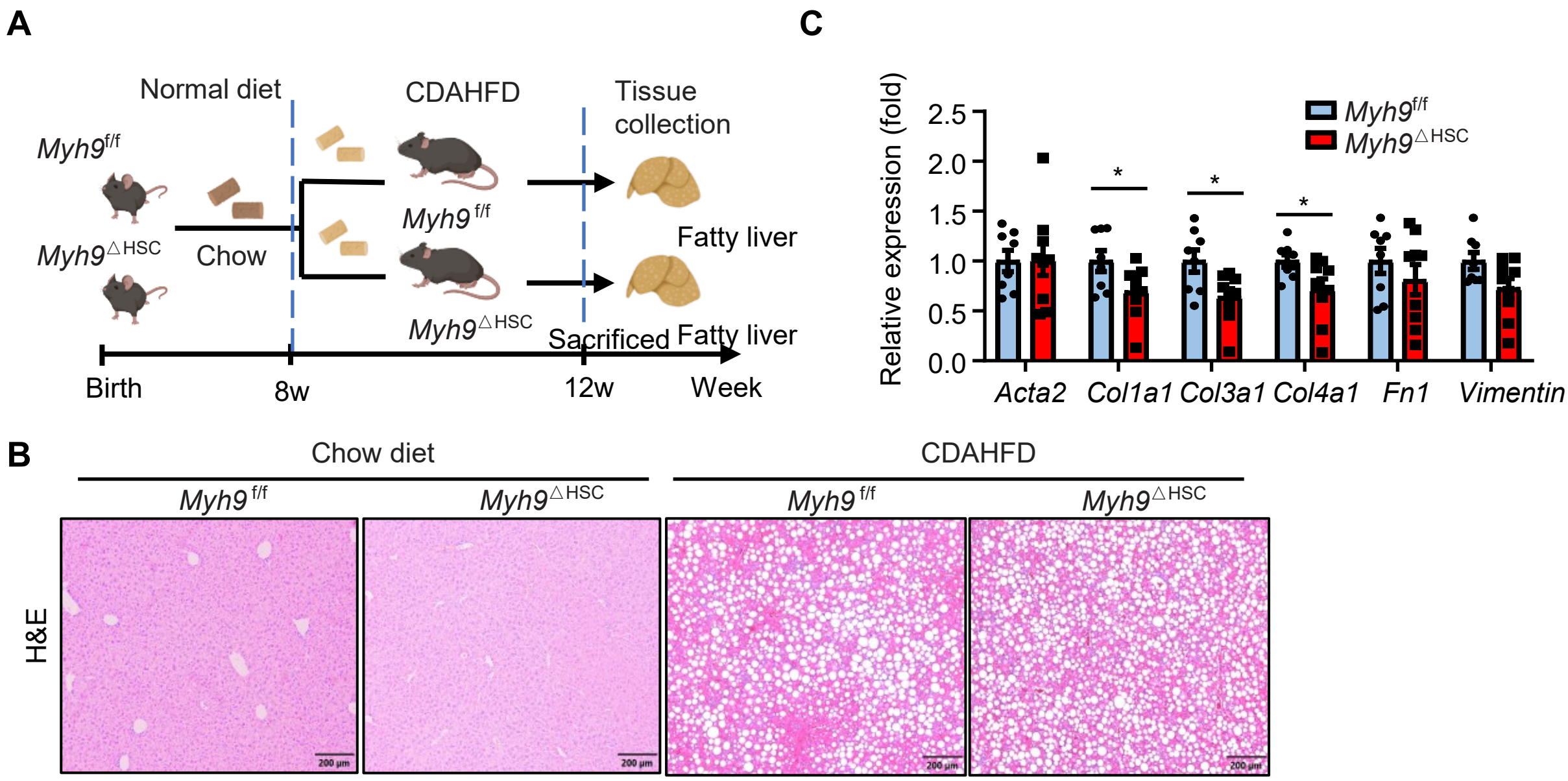

Supporting Figure S9. HSC-specific *Myh9* deficient mice are not resistant to CDAHFD-induced liver steatosis. (**A**) *Myh9*$^{f/f}$ and *Myh9*$^{\triangle HSC}$ mice were fed a CDAHFD for 4 weeks. Liver tissues were collected. **(B)** Representative images of H&E staining are shown. **(C)** RT-qPCR analyses of several hepatic fibrosis-related genes in CDA-HFD treated *Myh9*$^{f/f}$ and *Myh9*$^{\triangle HSC}$ mice. Values represent mean ± SEM. Significance was calculated using a 2-tailed Student's *t* test for comparing 2 groups (**C**). *P<0.05.

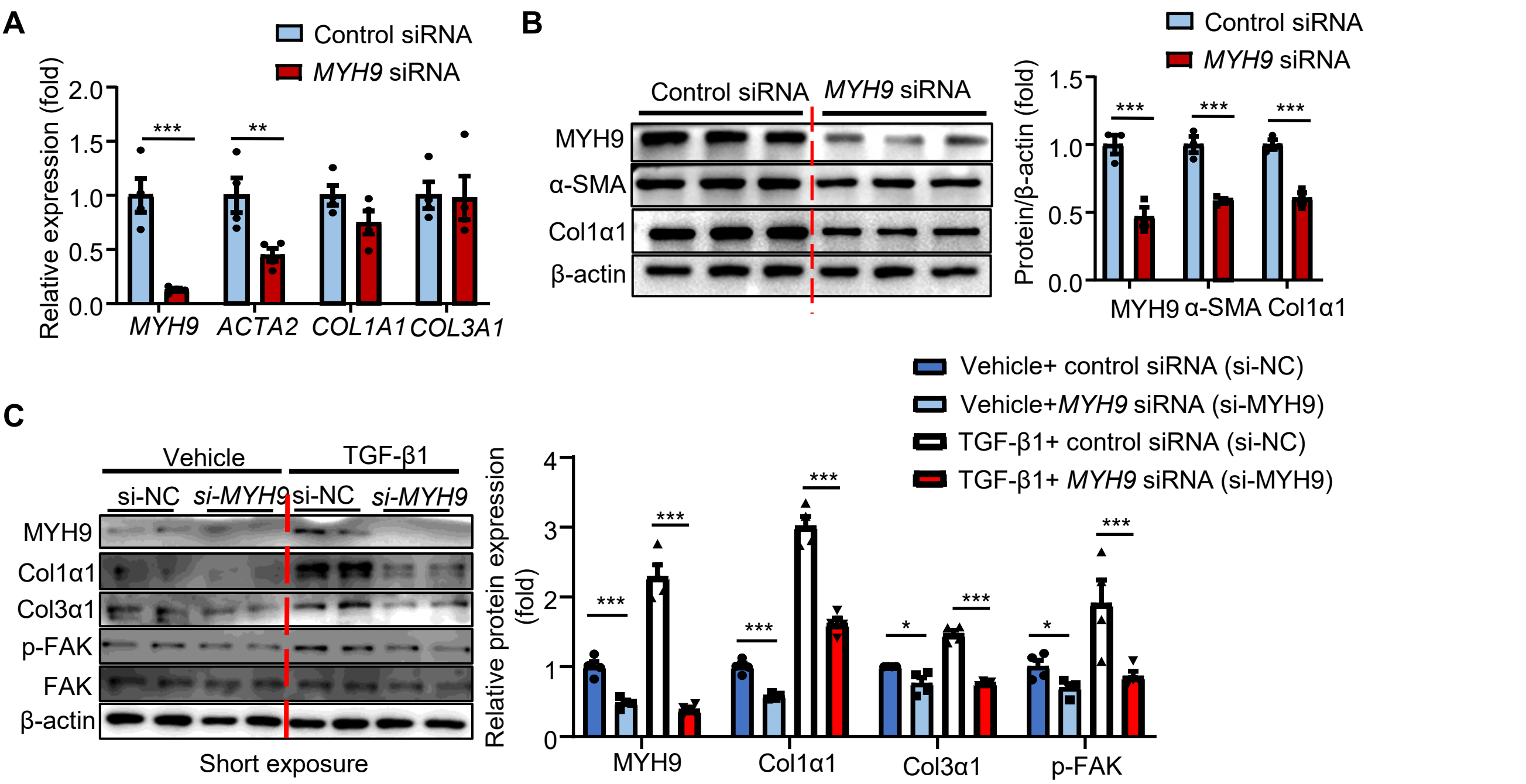

Supporting Figure S10. *Myh9* knockdown in HSCs limits its activation. (A) LX-2 cells were transfected with *MYH9* siRNA to silence MYH9 expression. RT-qPCR analyses of fibrosis-related genes. **(B)** Western blot analysis of MYH9 and fibrosis-related proteins. Relative protein levels were quantified. **(C)** Western blot analyses of MYH9, Col1α1, Col3α1, p-FAK, and FAK protein levels in LX-2 cells transfected with control siRNA or *MYH9* siRNA after TGF-β1 or vehicle treatment. Relative protein expression was quantified. Values represent mean ± SEM. Significance was calculated using a 1-way ANOVA for multiple groups (**C**) and a 2-tailed Student's *t* test for comparing 2 groups (**A** and **B**). **P<0.01, ***P<0.001.

Supporting Figure S11

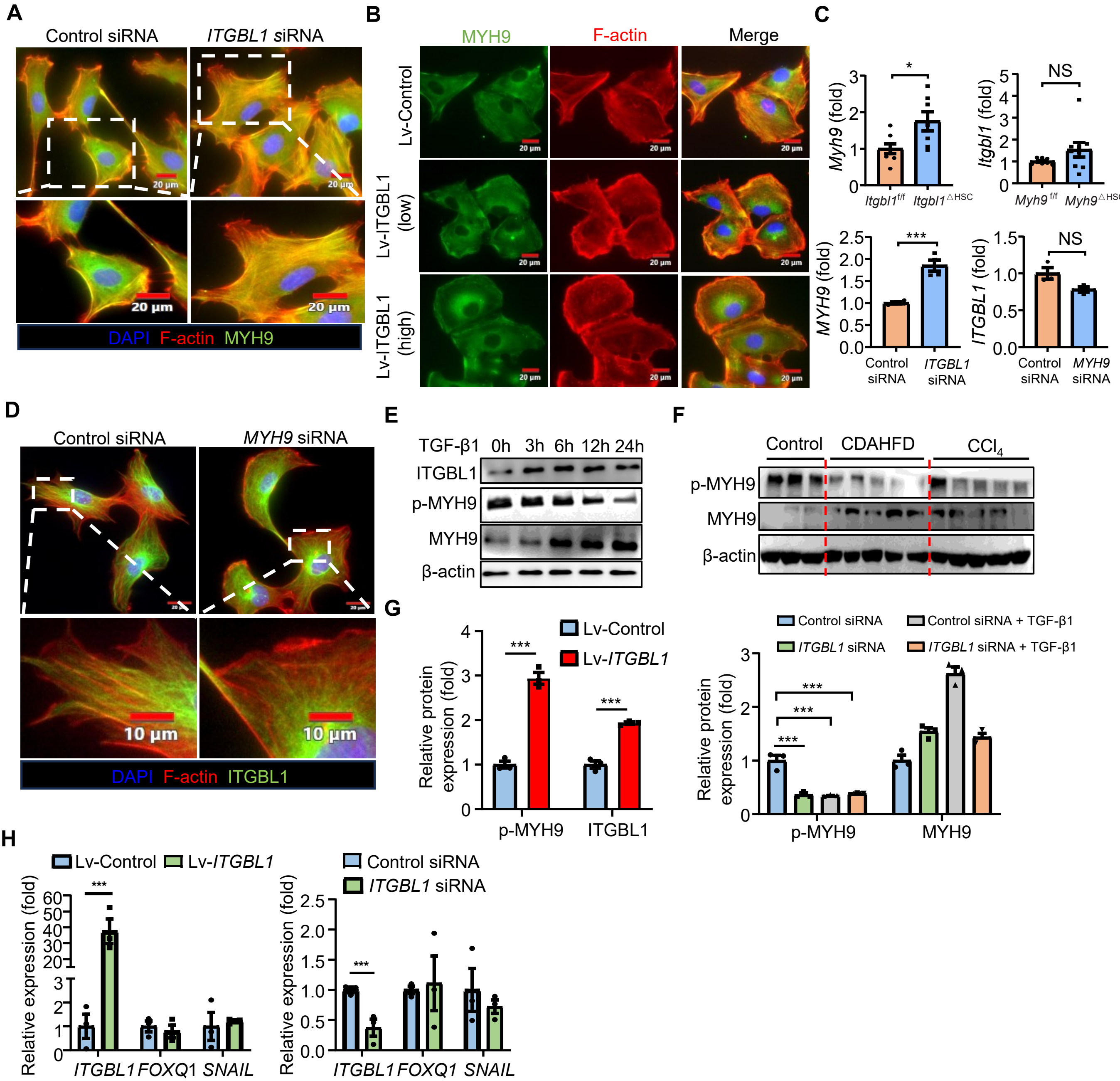

Supporting Figure S11. Reciprocal regulation between ITGBL1 and MYH9 coordinately controls actomyosin assembly. **(A)** LX-2 cells were infected with control siRNA and *ITGBL1* siRNA for 48 hours. Representative images of immunofluorescence of MYH9 (green) and F-actin (red) are shown. **(B)** LX-2 cells were transfected with control, low-dose of Lv-ITGBL1 and high-dose of Lv-ITGBL1. Representative images of immunofluorescence of MYH9 (green) and F-actin (red) are shown. **(C)** RT-qPCR analyses of hepatic *Myh9* or *Itgbl1* expression in CDAHFD-fed *Itgbl1*$^{f/f}$ and *Itgbl1*$^{\triangle HSC}$ or *Myh9*$^{f/f}$ and *Myh9*$^{\triangle HSC}$ liver tissues (upper panel). RT-qPCR analyses of *MYH9* or *ITGBL1* expression in *ITGBL1* or *MYH9* siRNA-infected LX-2 cells (lower panel). **(D)** LX-2 cells were infected with control siRNA and *MYH9* siRNA for 48 hours. Representative images of immunofluorescence of ITGBL (green) and F-actin (red) are shown. **(E)** LX-2 cells were treated with TGF-β1 (5 ng/ml) for different times. The expression of different proteins was measured by western blotting. **(F)** Western blot analyses of protein levels of p-MYH9, MYH9, and β-actin in liver tissues. Relative protein levels were quantified. **(G)** LX-2 cells were transfected with Lv-ITGBL1 or *ITGBL1* siRNA for 48h. Relative protein levels were quantified. **(H)** RT-qPCR analyses several genes in Lv-ITGBL1 or ITGBL1 siRNA-infected LX-2 cells. Values represent mean ± SEM. Significance was calculated 2-tailed Student's *t* test for comparing 2 groups (**B**, **C** and **F**). *P<0.05, ***P<0.001.

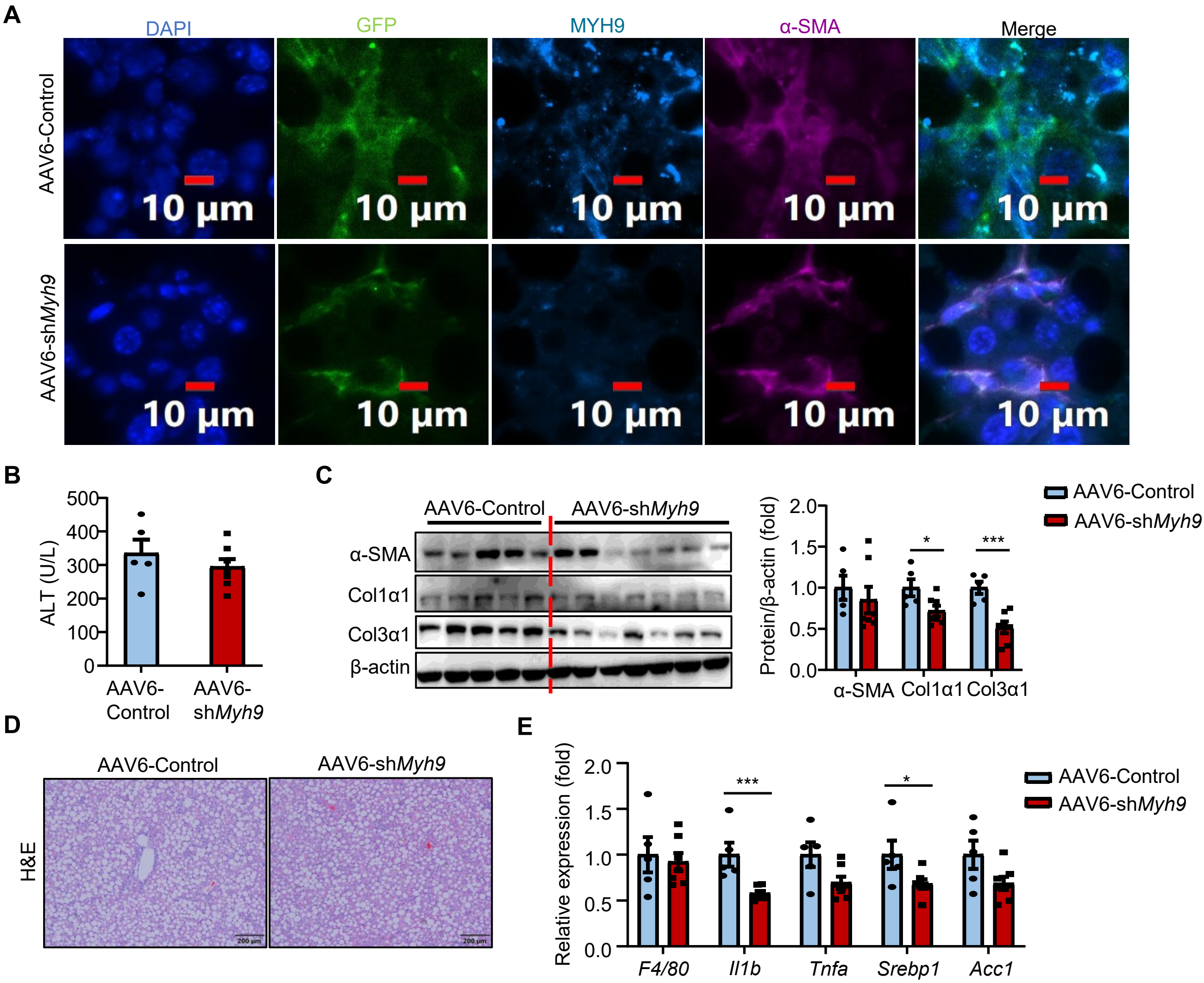

Supporting Figure S12. HSC-specific *Myh9* knockdown mitigates CDAHFD-induced liver fibrosis. (A) CDAHFD-fed WT mice were injected with AAV6-control and AAV6-sh*Myh9*. Liver tissues were collected. *Myh9* knockdown in HSCs was verified by performing immunofluorescence staining of MYH9 in the liver. Representative images of immunofluorescence staining of GFP (green), MYH9 (blue) and α-SMA (purple) in CDAHFD-fed CCl_4-induced liver fibrosis mice are shown. Scale bar, 10 μm. **(B)** Western blot analyses of fibrosis proteins in CDAHFD-fed AAV6-shMyh9 and AAV6-control mice. Relative protein expression was quantified. **(C)** Serum ALT levels were measured. **(D)** Representative images of H&E staining are shown. **(E)** RT-qPCR analyses of several fibrosis genes in CDAHFD-fed AAV6-shMyh9 and AAV6-control mice. Values represent mean ± SEM. Significance was calculated using a 1-way ANOVA for multiple groups (**C** and **E**). *P<0.05, ***P<0.001.

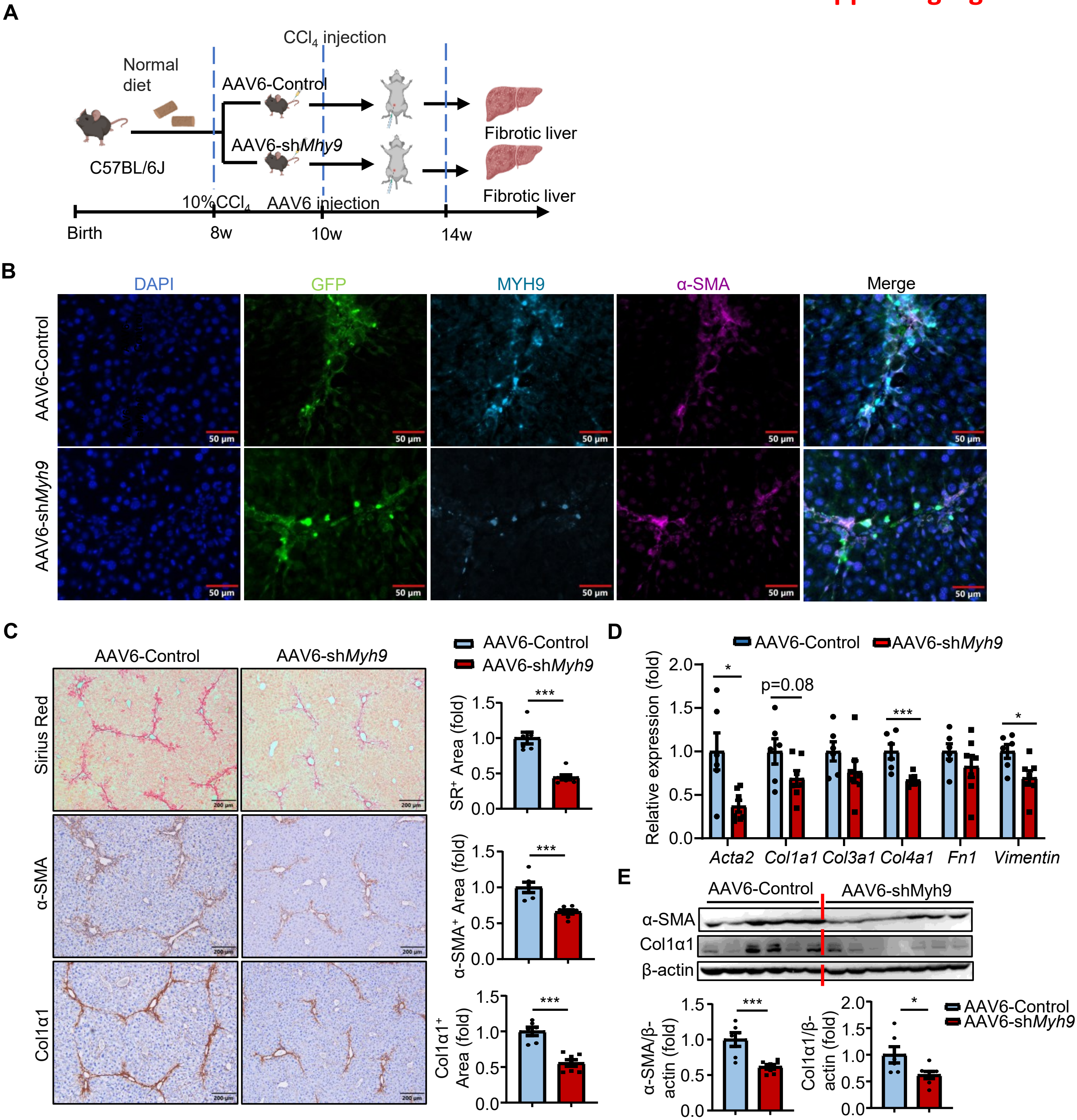

Supporting Figure S13. HSC-specific *Myh9* knockdown ameliorates CCl_4-induced liver fibrosis. (A) The regimen of targeting HSCs to specifically knock down MYH9 by injection of AAV6. **(B)** *Myh9* knockdown in HSCs was verified by performing immunofluorescence staining of MYH9 in the liver. Representative images of immunofluorescence staining of GFP (green), MYH9 (blue) and α-SMA (purple) in CCl_4-induced liver fibrosis mice are shown. Scale bar, 50 μm. **(C)** Representative images of Sirius red, α-SMA and Col1α1 staining are shown. The degree of fibrosis was quantified. **(D)** RT-qPCR analyses of several fibrosis genes in CCl_4-treated AAV6-shMyh9 and AAV6-control mice. **(E)** Western blot analyses of fibrosis proteins in CCl_4-treated AAV6-shMyh9 and AAV6-control mice. Relative protein expression was quantified. Values represent mean ± SEM. Significance was calculated using a 1-way ANOVA for multiple groups (**C** and **E**). *P<0.05, ***P<0.001.

Supporting Figure S14

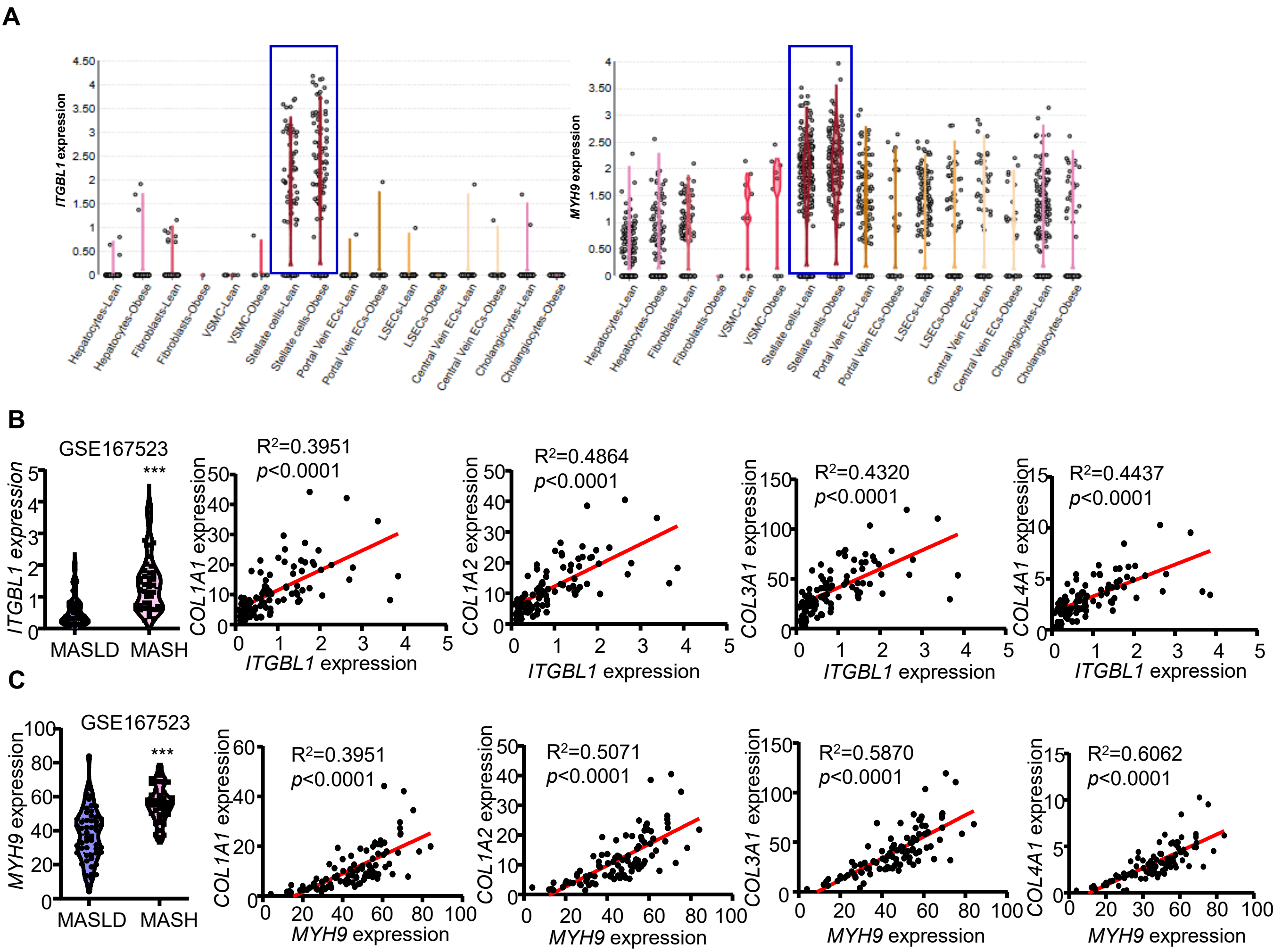

Supporting Figure S14. Hepatic *ITGBL1* and *MYH9* levels positively correlate with fibrogenic genes in patients with MASH. (A) Hepatic *ITGBL1* and *MYH9* expressions in lean and obese individuals were analyzed by scRNA-seq from Liver Cell Atlas database (https://www.livercellatlas.org/index.php). **(B)** Hepatic *ITGBL1* levels and fibrogenic genes was analyzed in patients with MASLD in a public database (GSE167523). Correlation between *ITGBL1* and fibrogenic genes was analyzed. (**C**) Hepatic *MYH9* levels and fibrogenic genes was analyzed in patients with MASLD in a public database (GSE167523). Correlation between *MYH9* and fibrogenic genes was analyzed. Significance was calculated using a 1-way ANOVA for multiple groups (**B** and **C**)
