# Supplementary material for: ITGBL1-MYH9 interaction in hepatic stellate cells acts as a mechanoregulator controlling liver fibrosis in mice
Source: J Clin Invest. 2026 Jul 16;136(18):e203412. doi: 10.1172/JCI203412 (PMC13574165; doi:10.1172/JCI203412)

Uncropped Images of the original western blots

Figure 4C

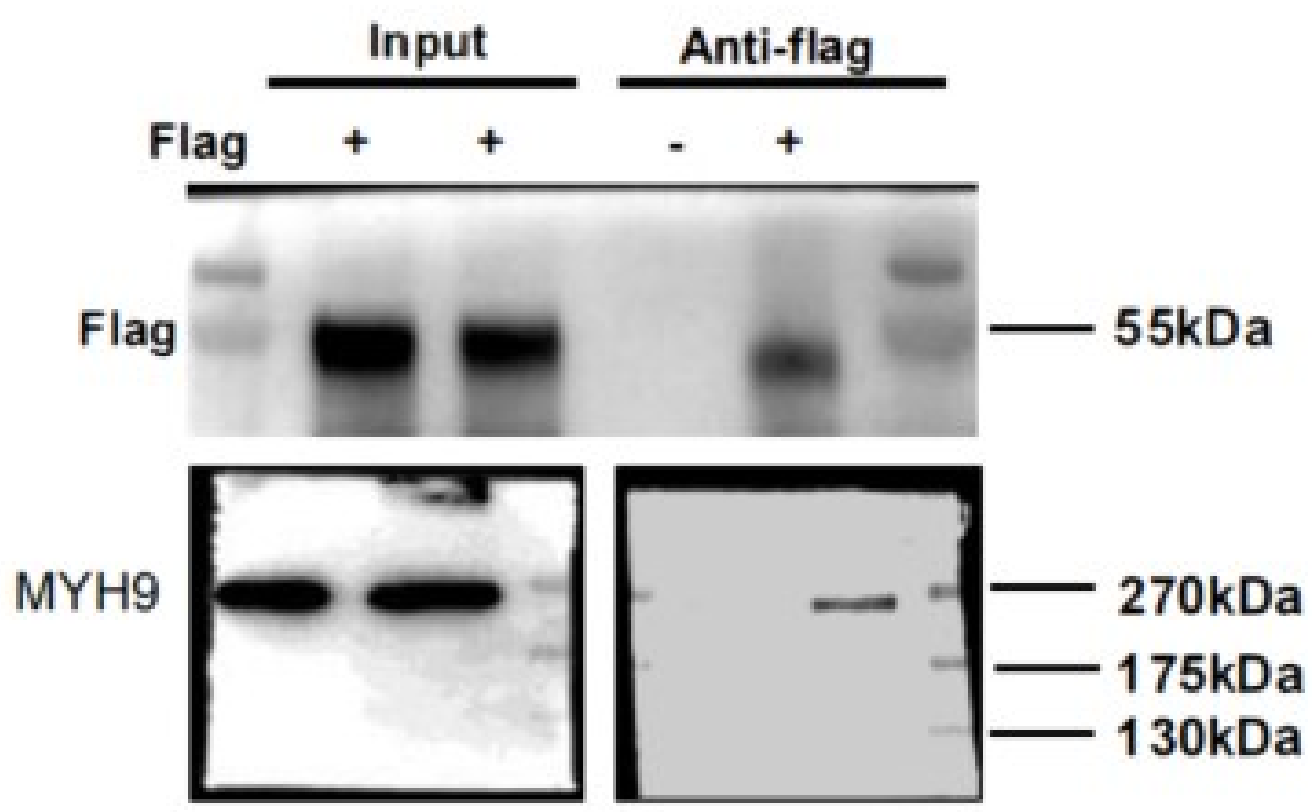

Figure 5E

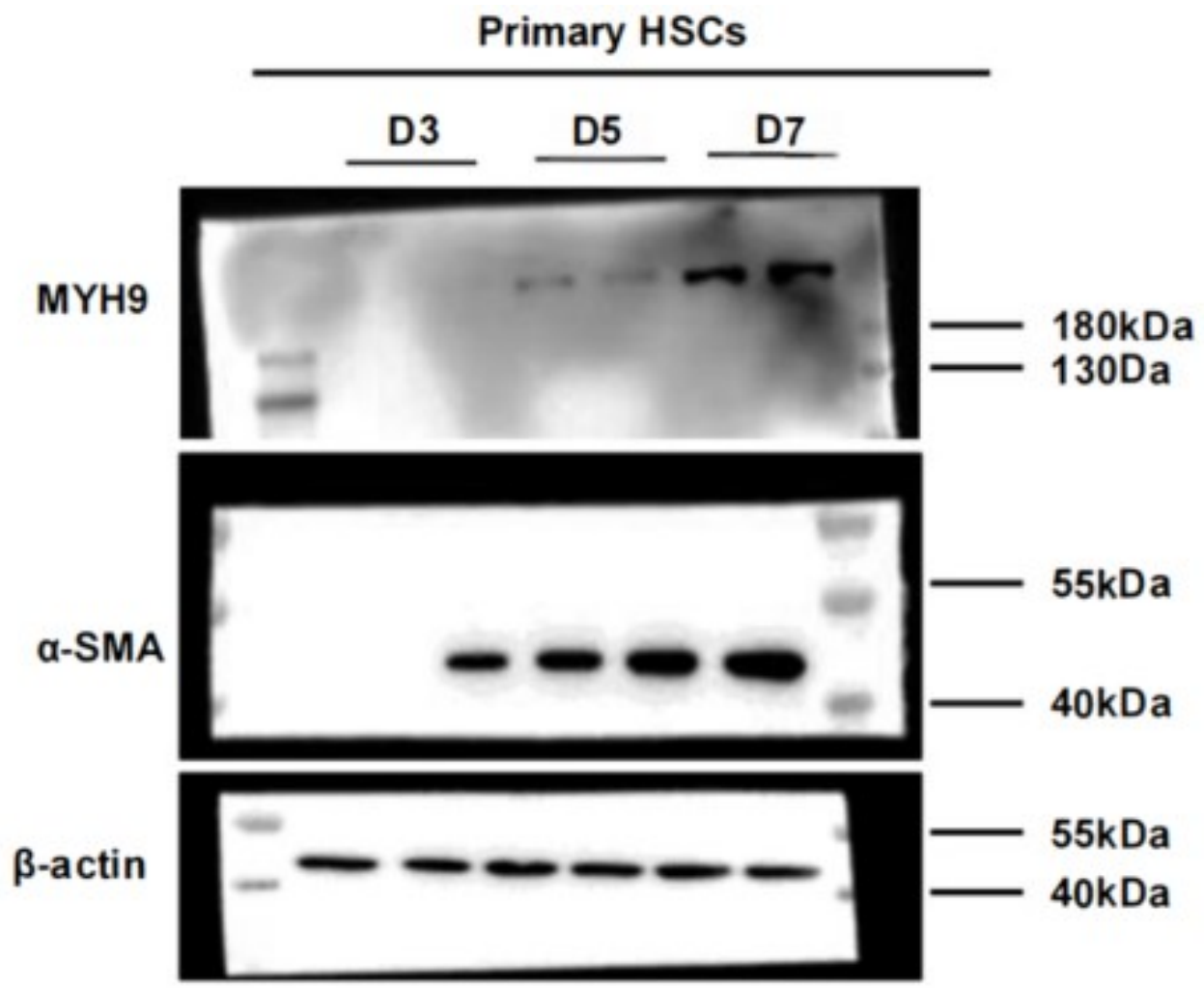

Figure 5F

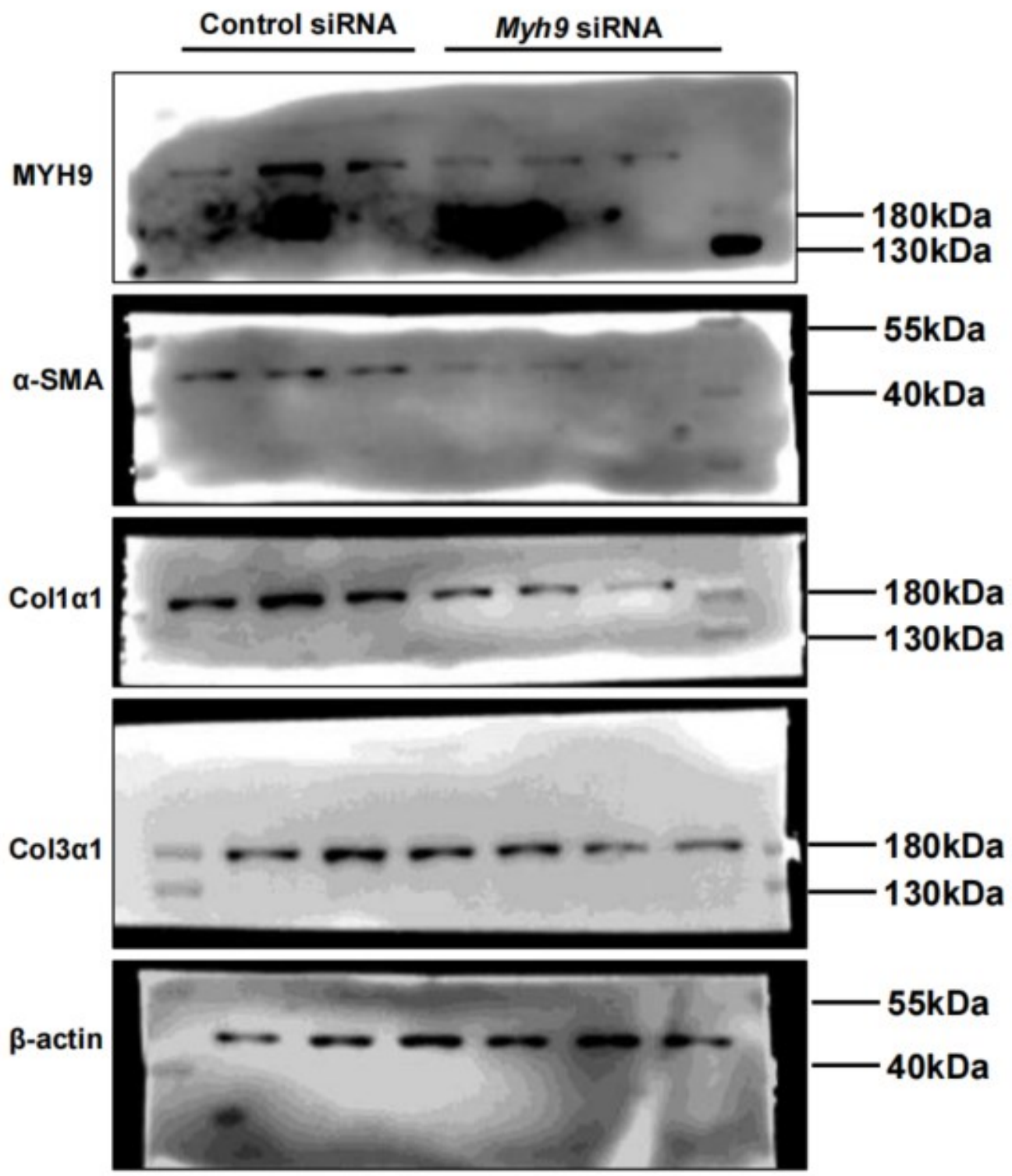

Figure 6B

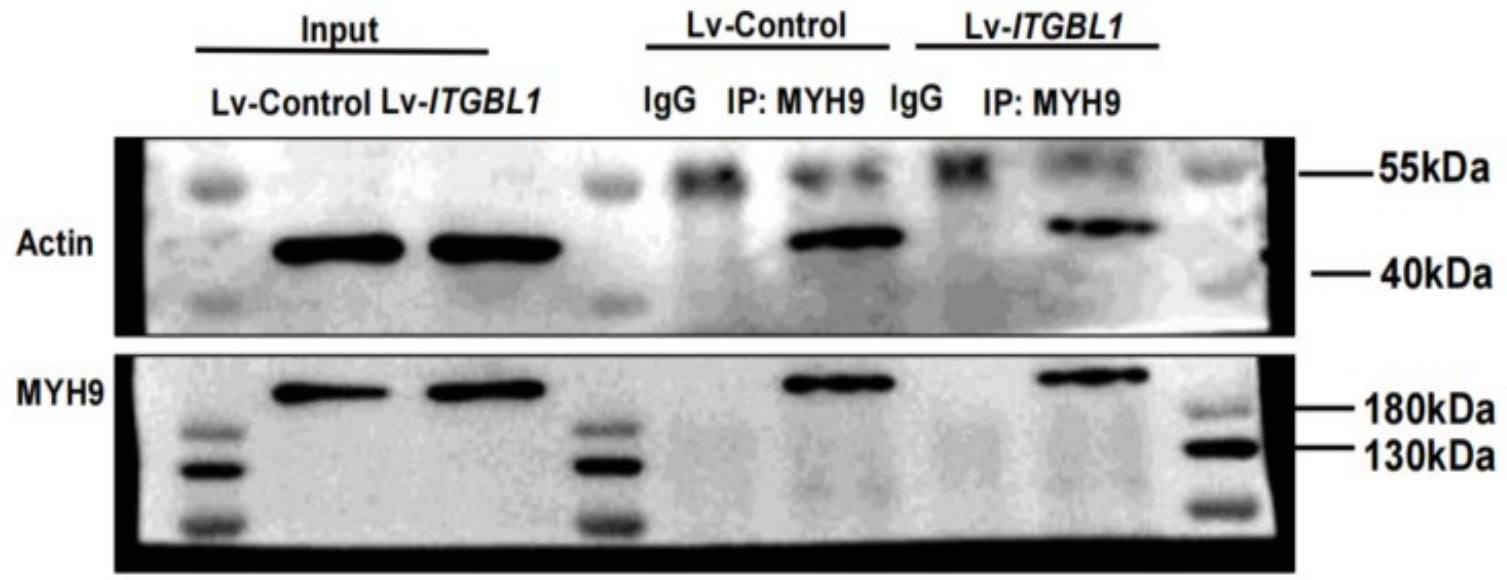

Figure 6C

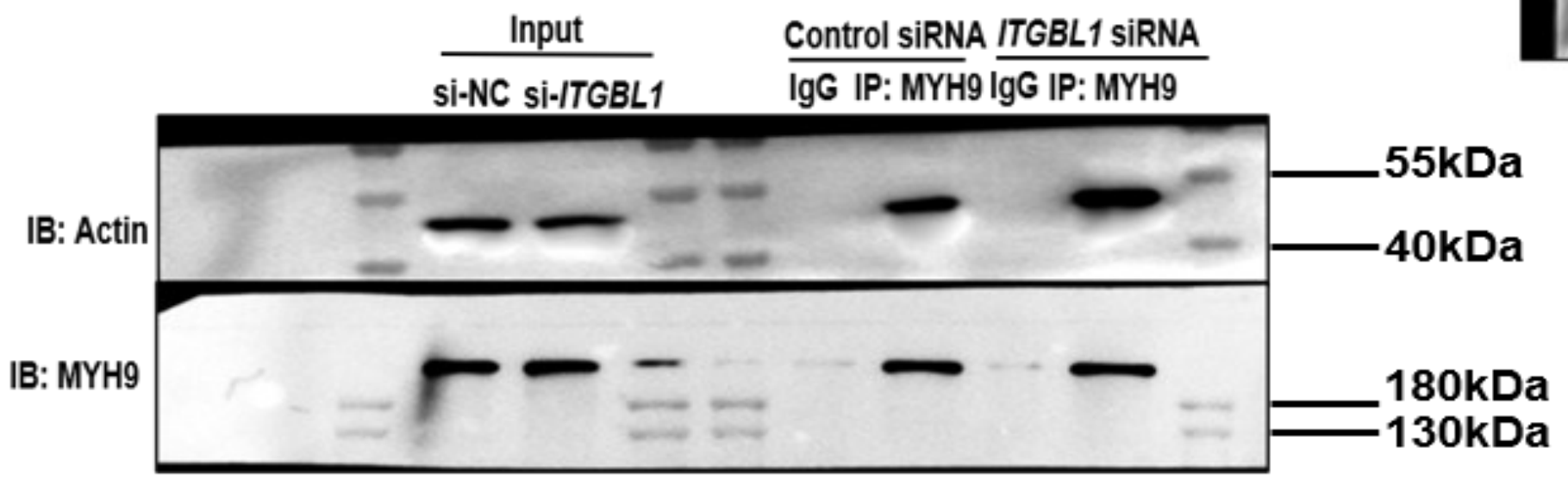

Uncropped Images of the original western blots

Figure 6D

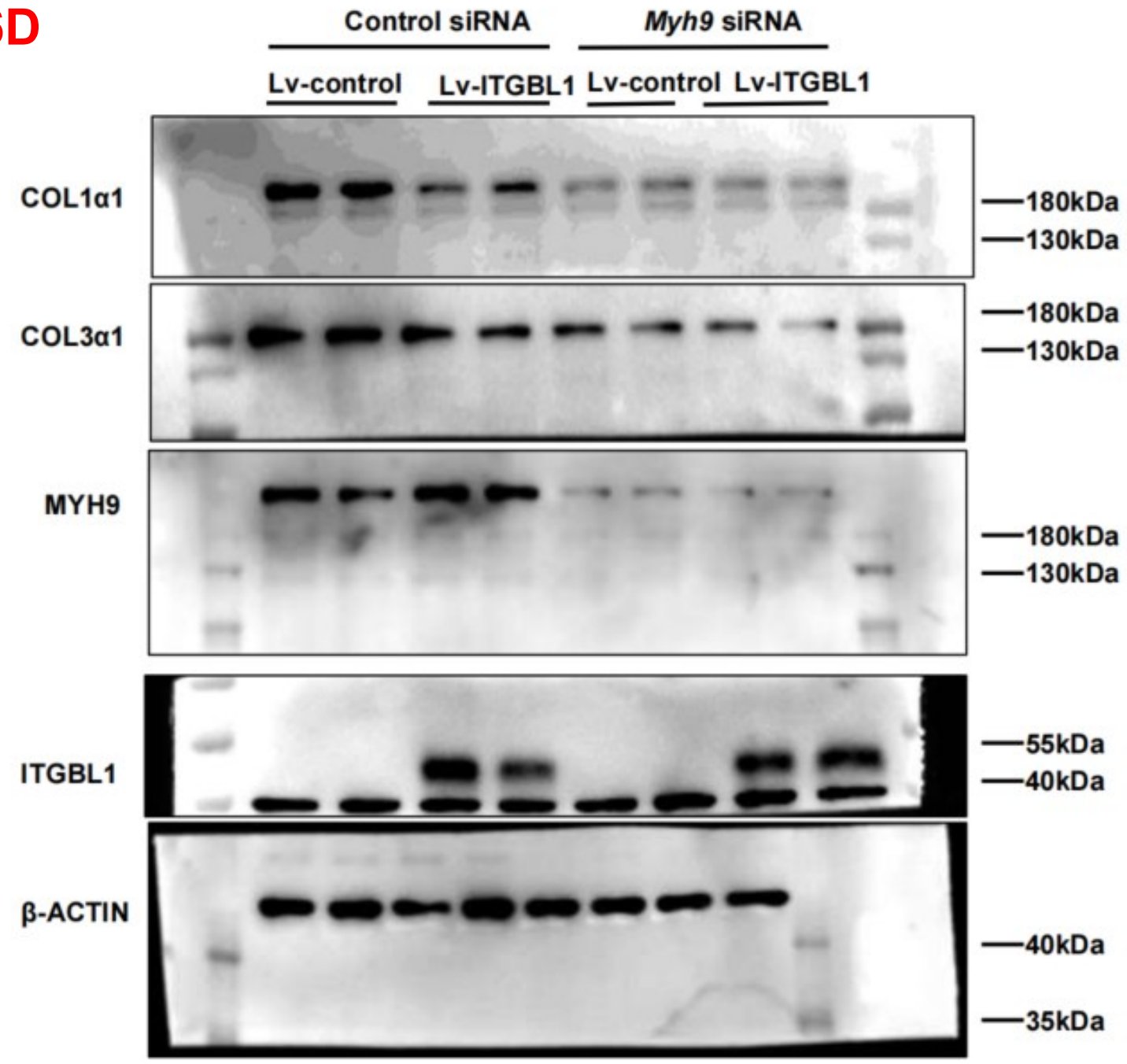

Figure 6G

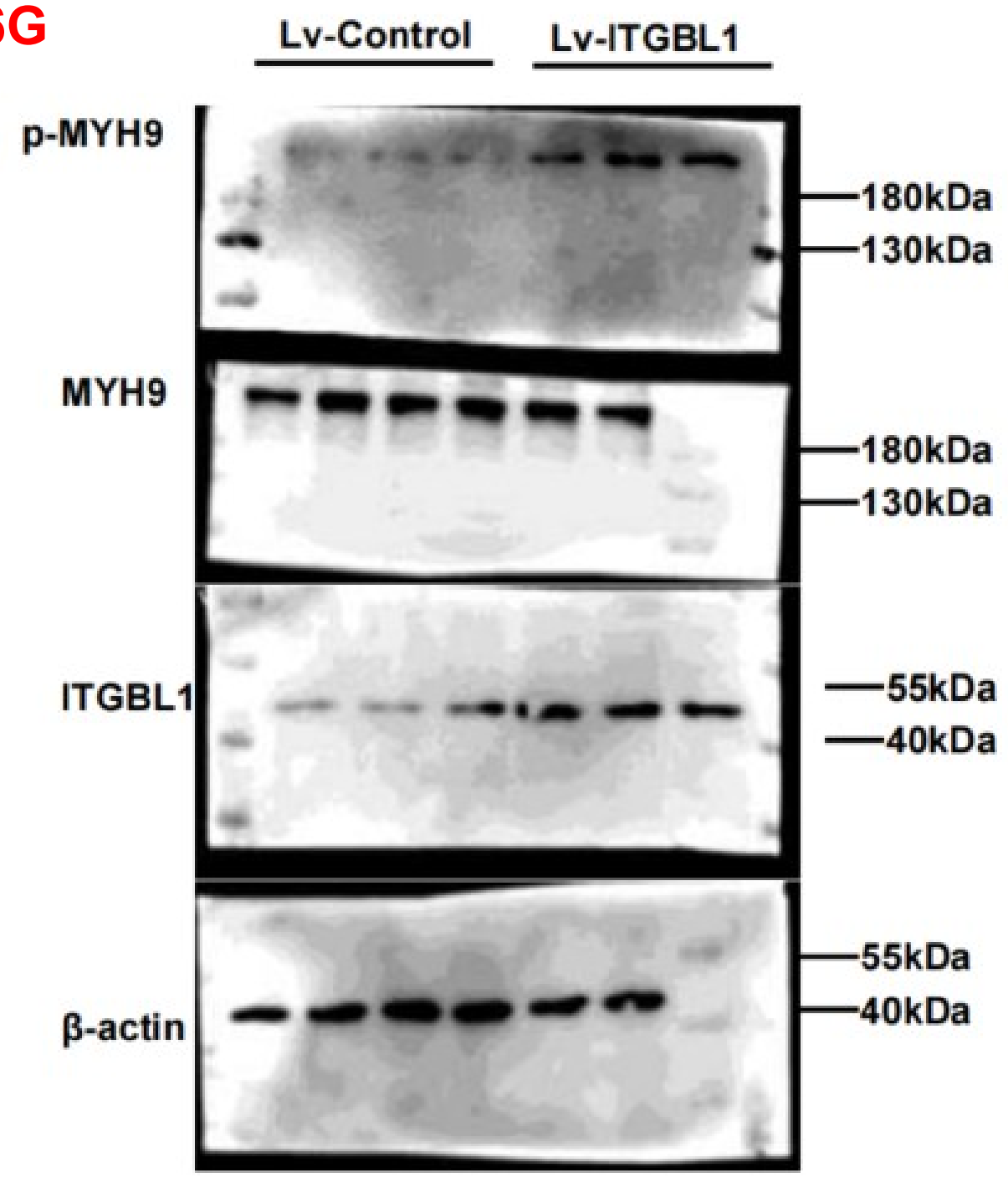

Figure 6H

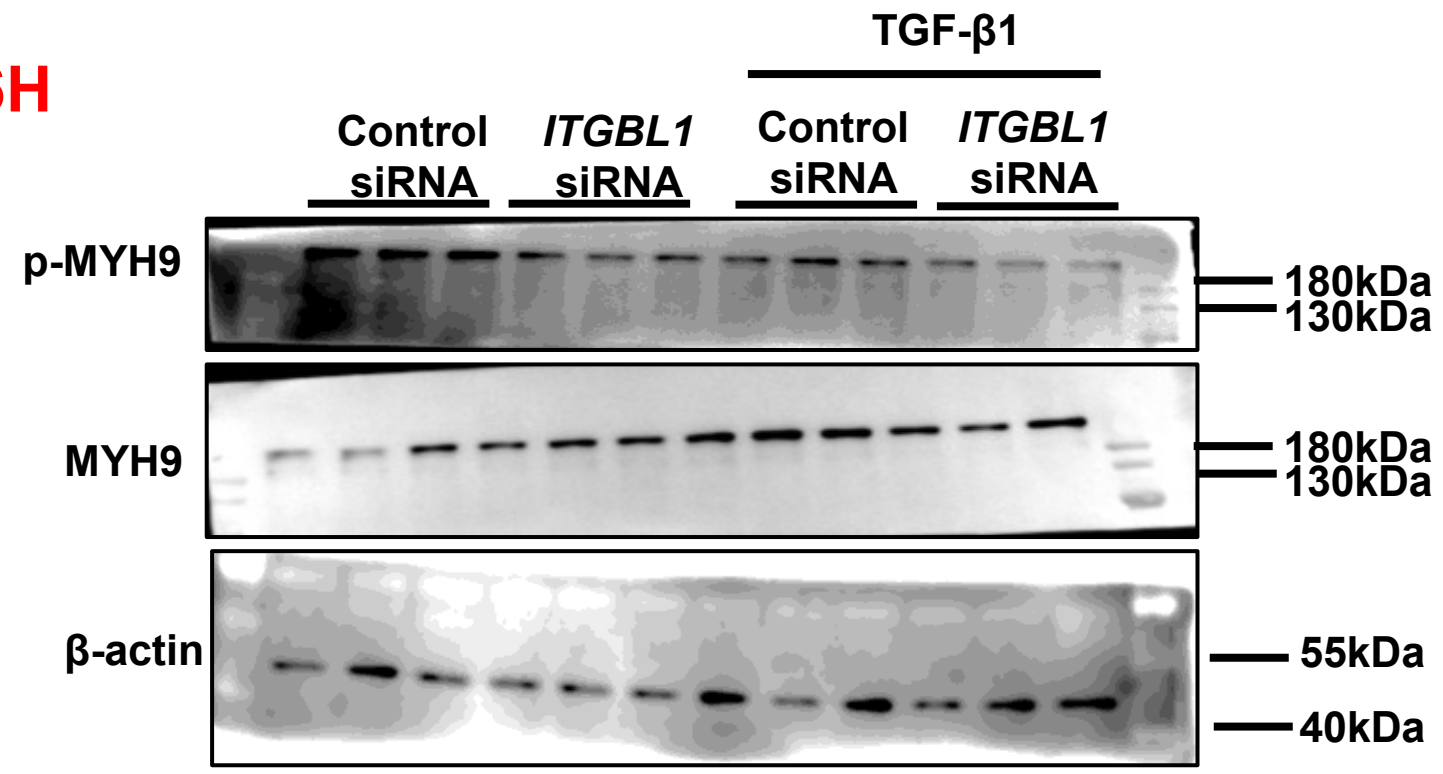

Figure 7B

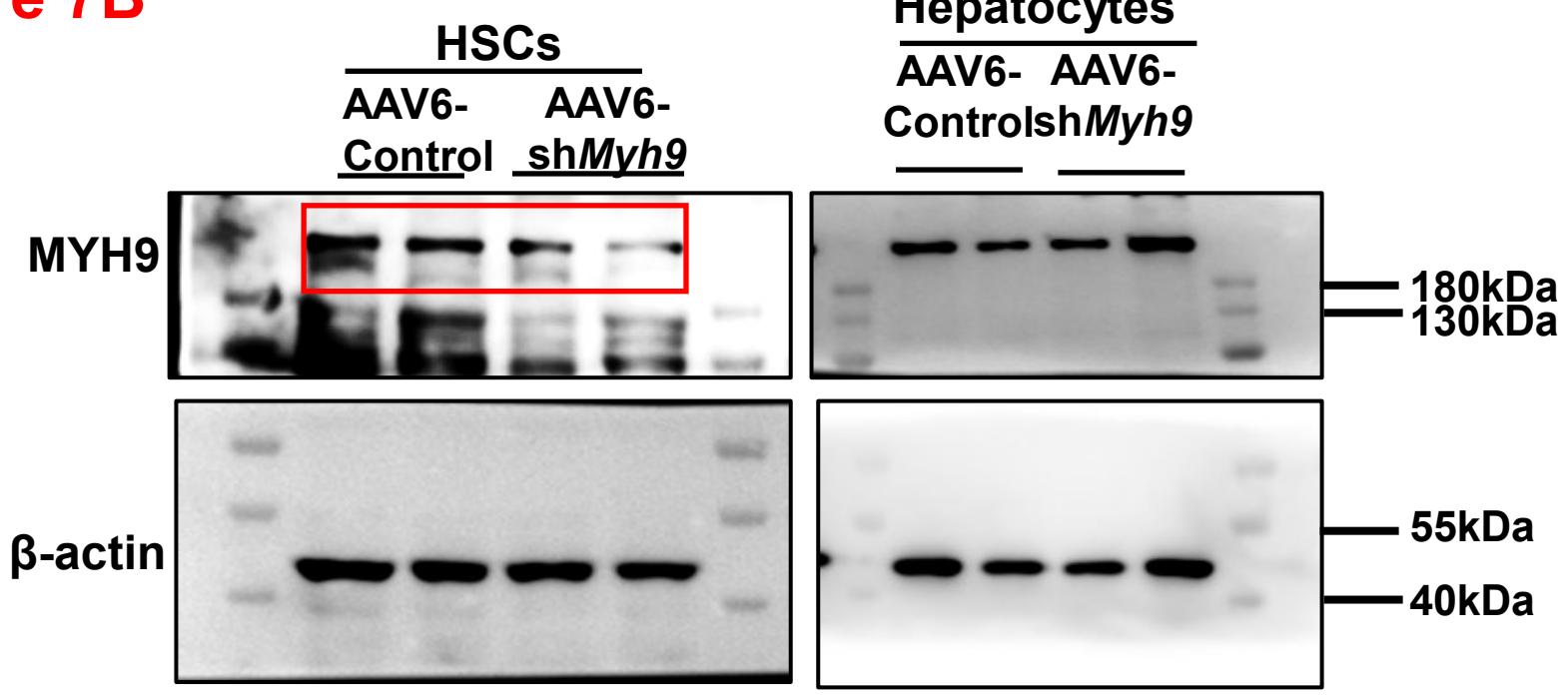

Figure S2A

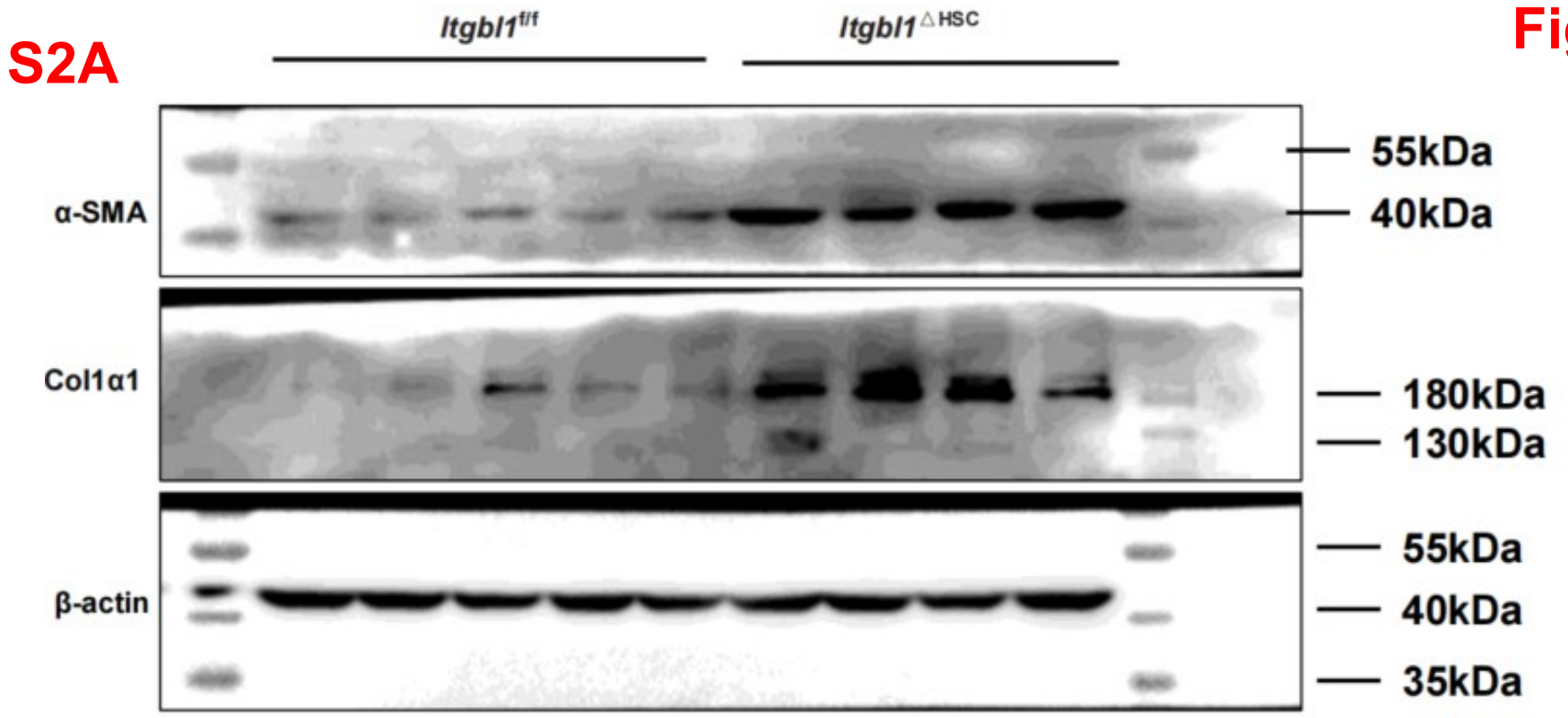

Figure S3H

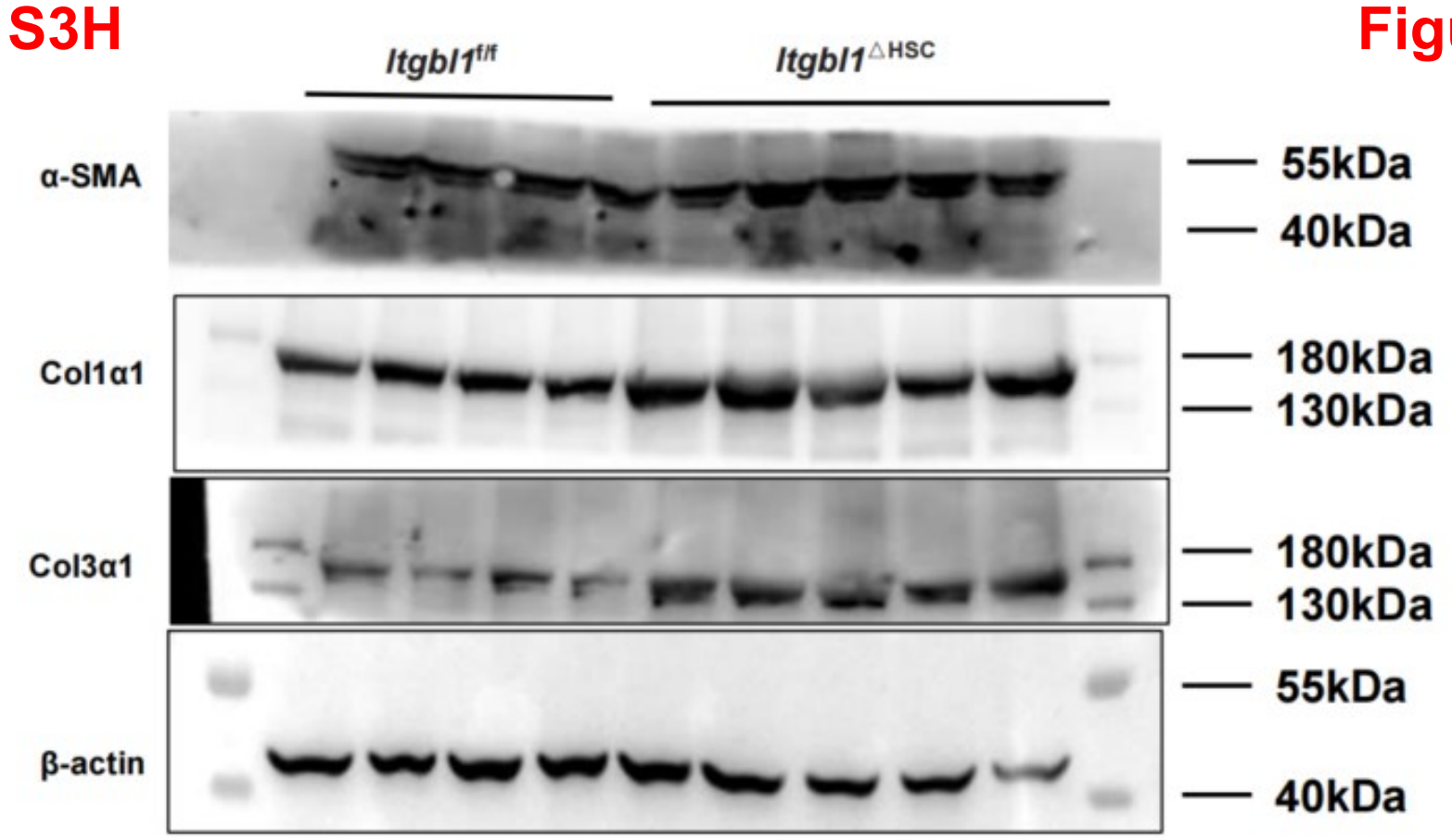

Figure S5A

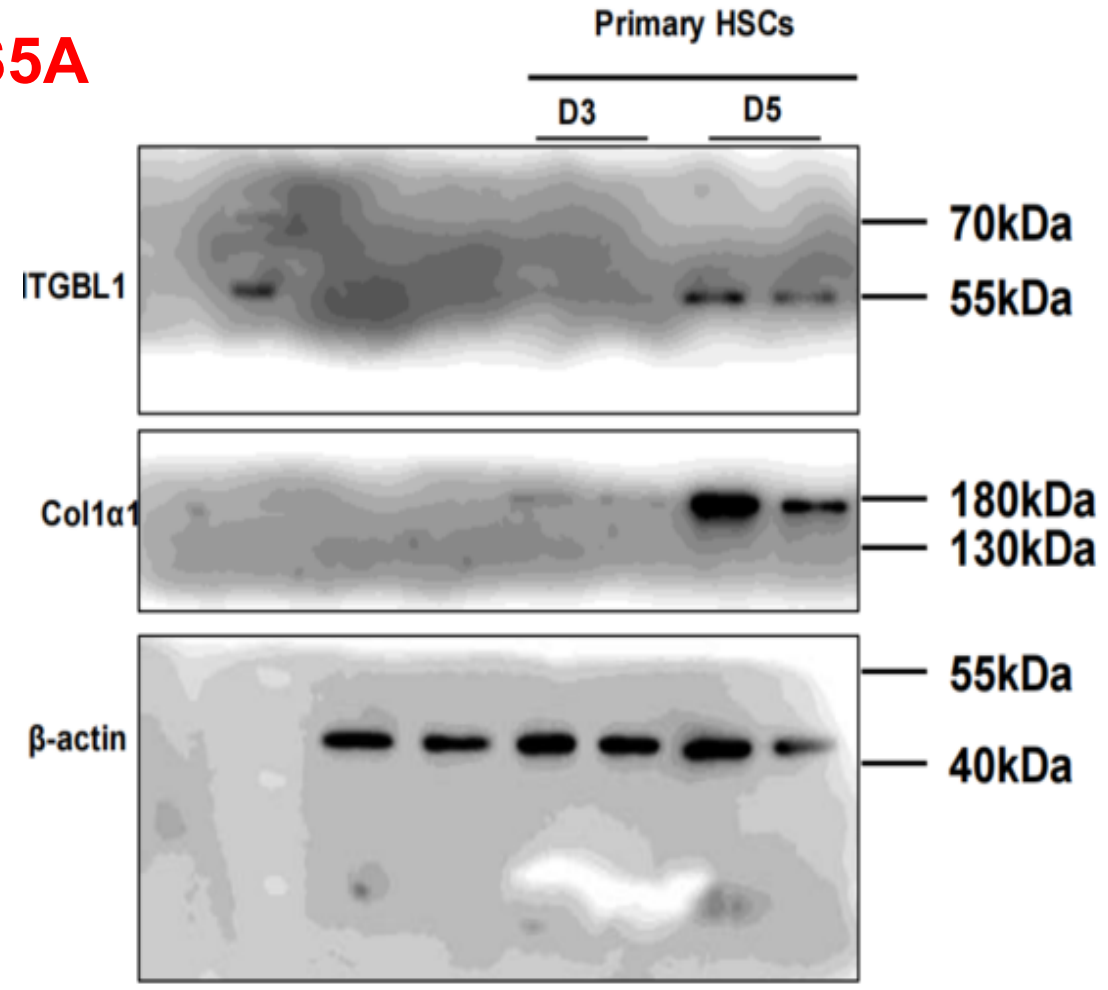

Uncropped Images of the original western blots

Figure S5D

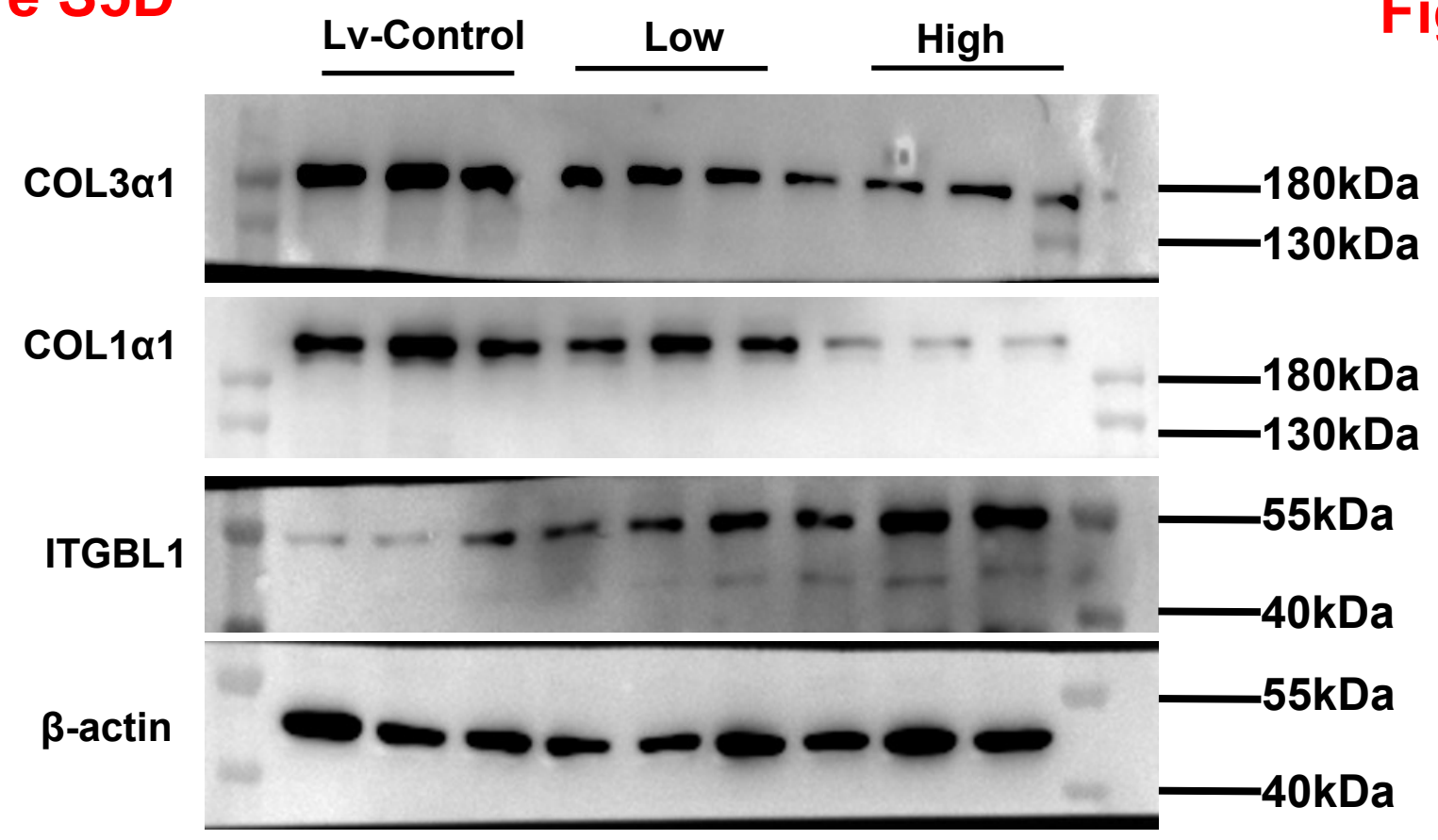

Figure S6A

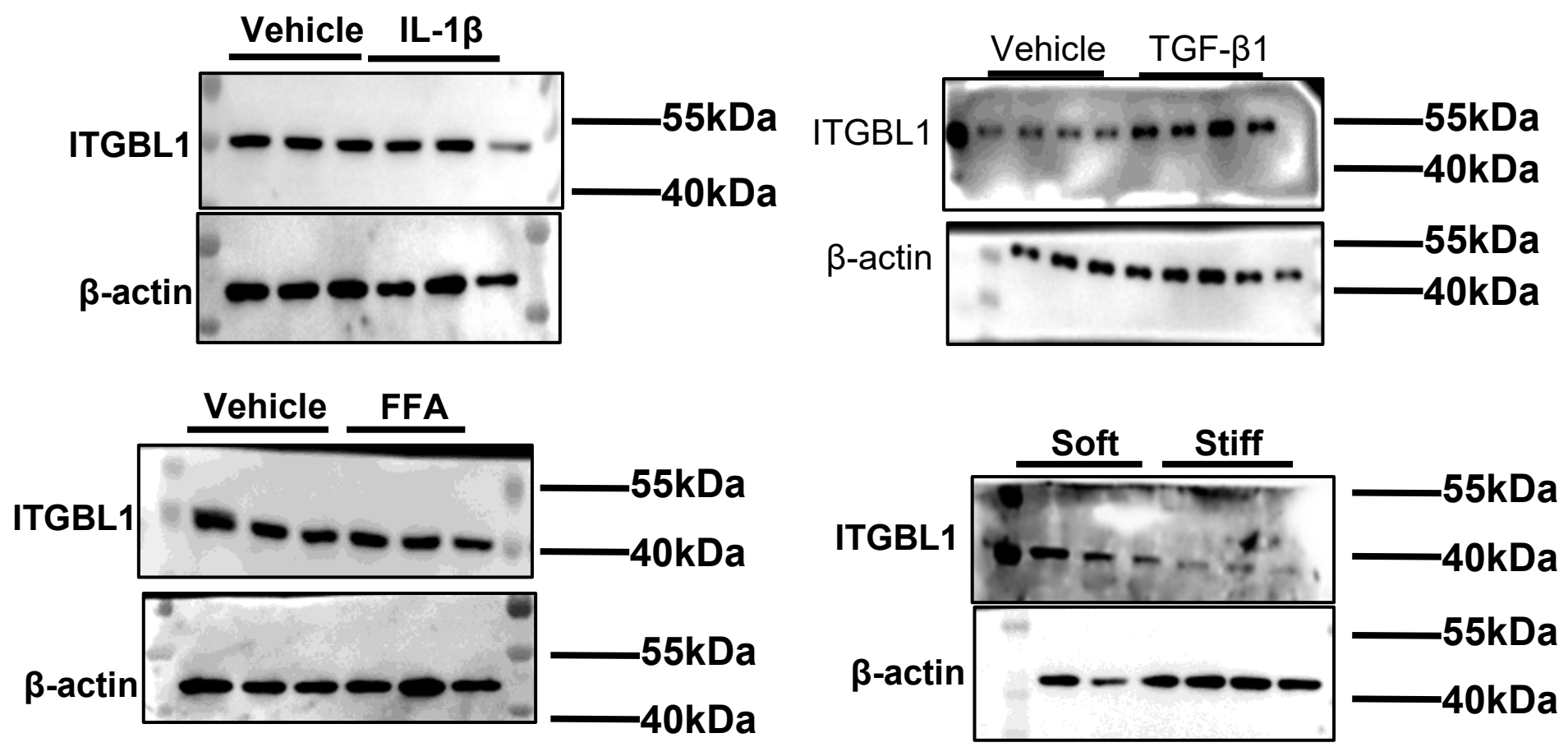

Figure S6G

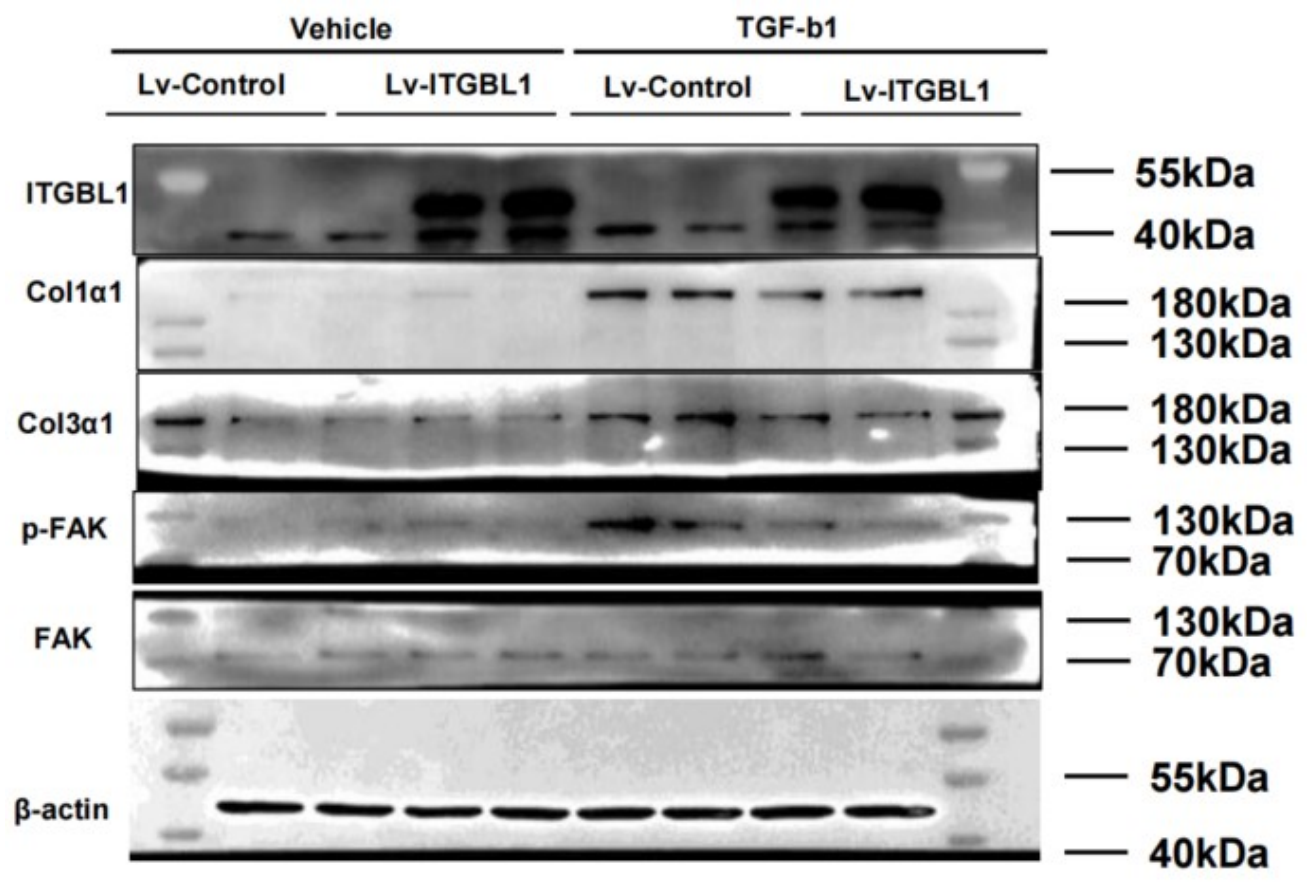

Figure S10B

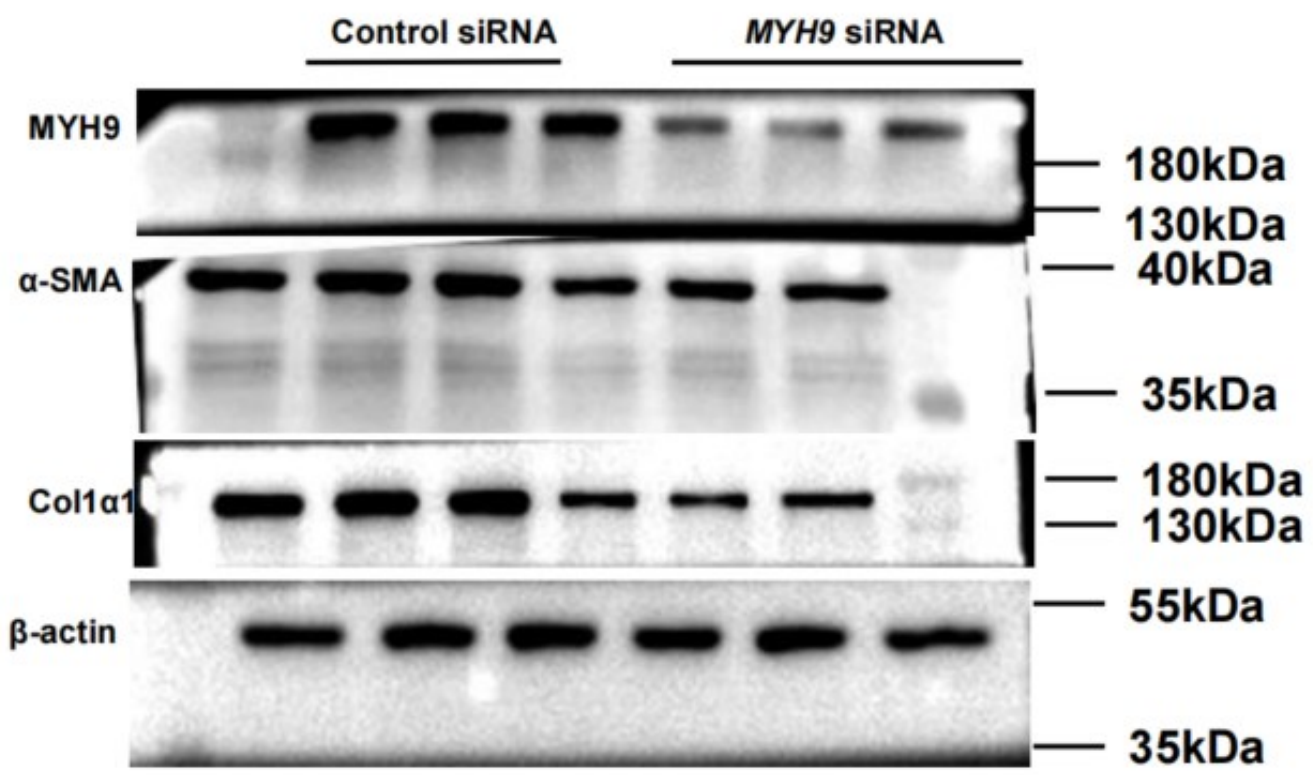

Figure S10C

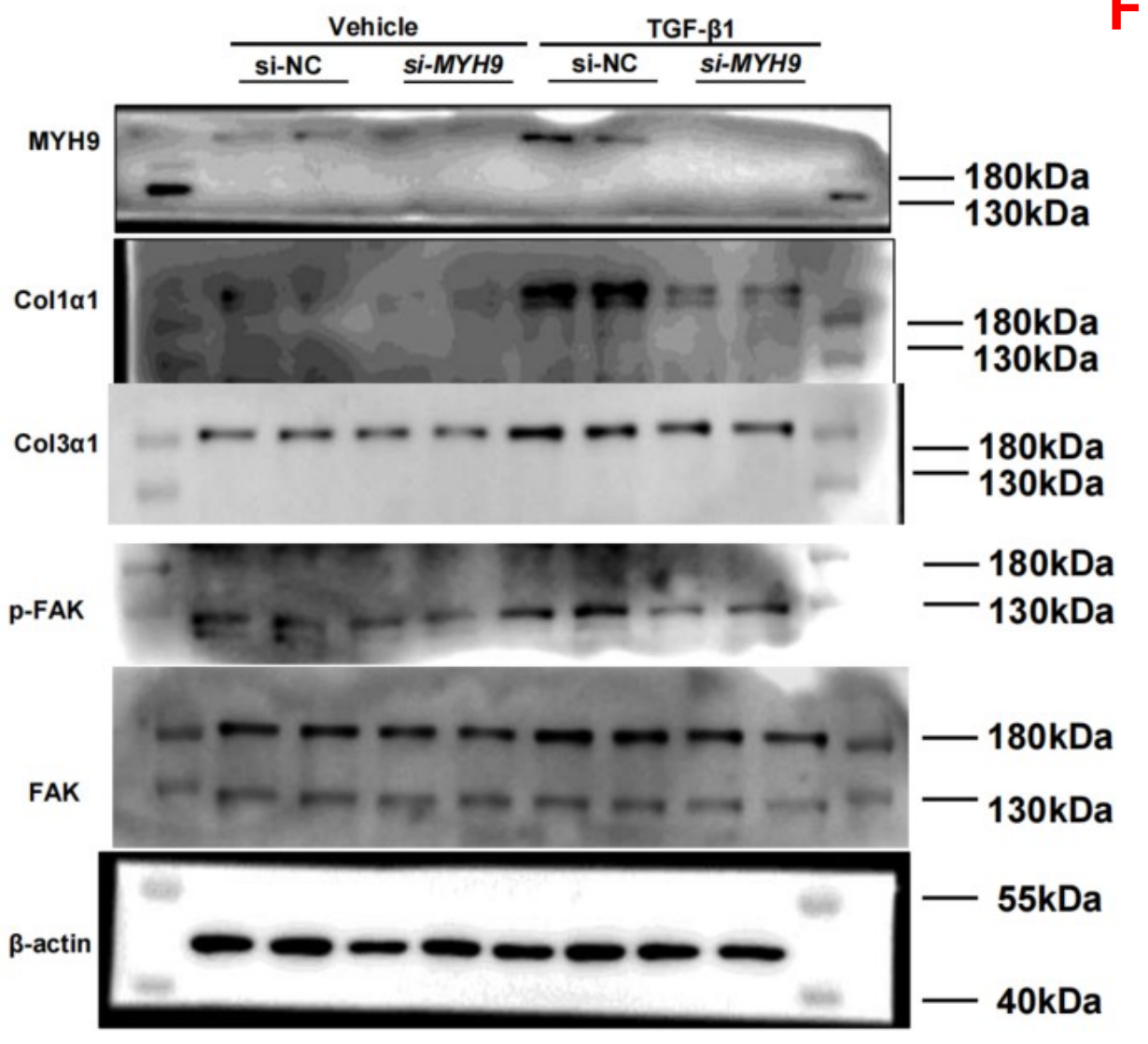

Short exposure

Figure S11E

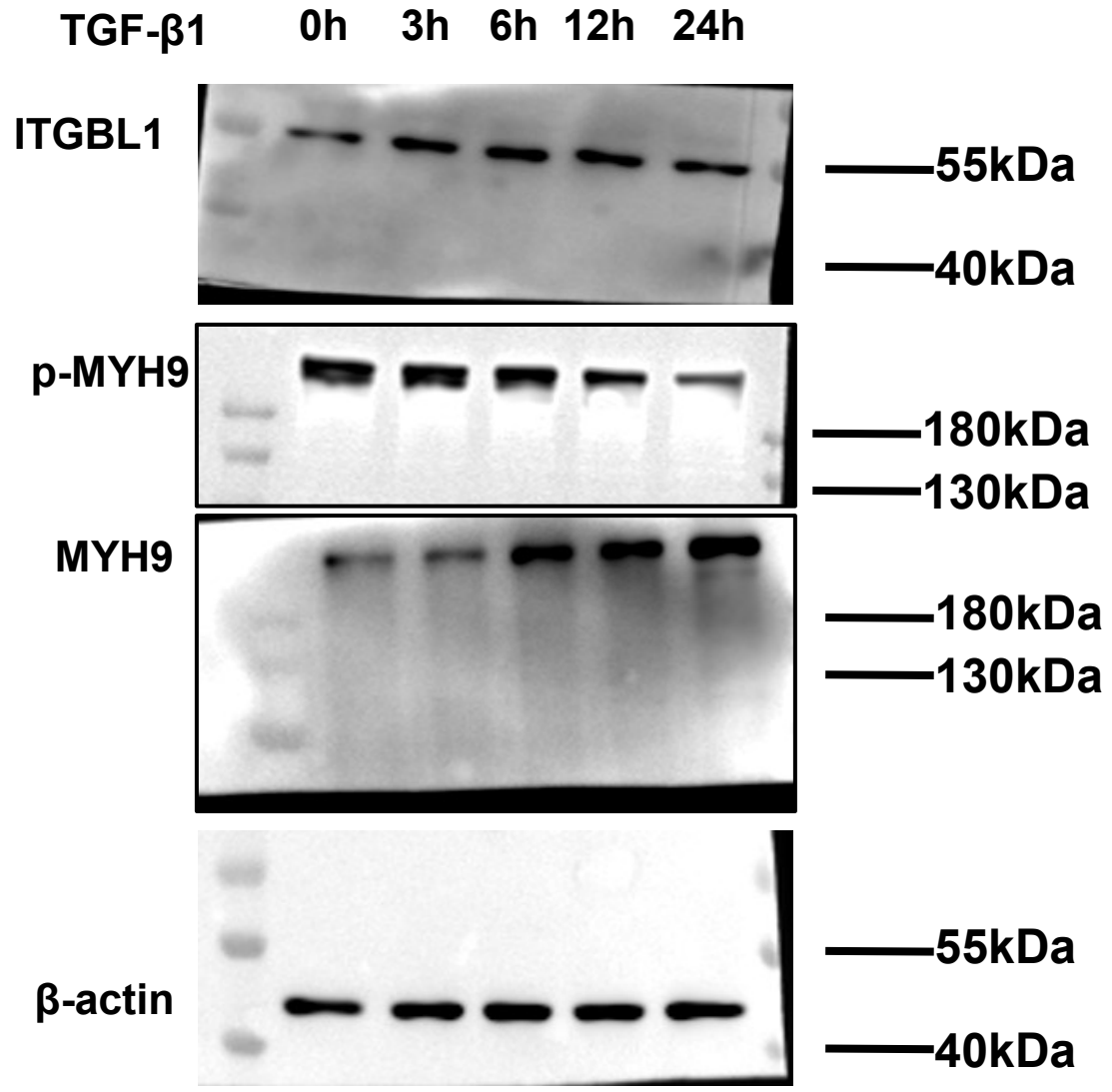

Uncropped Images of the original western blots

Figure S11F

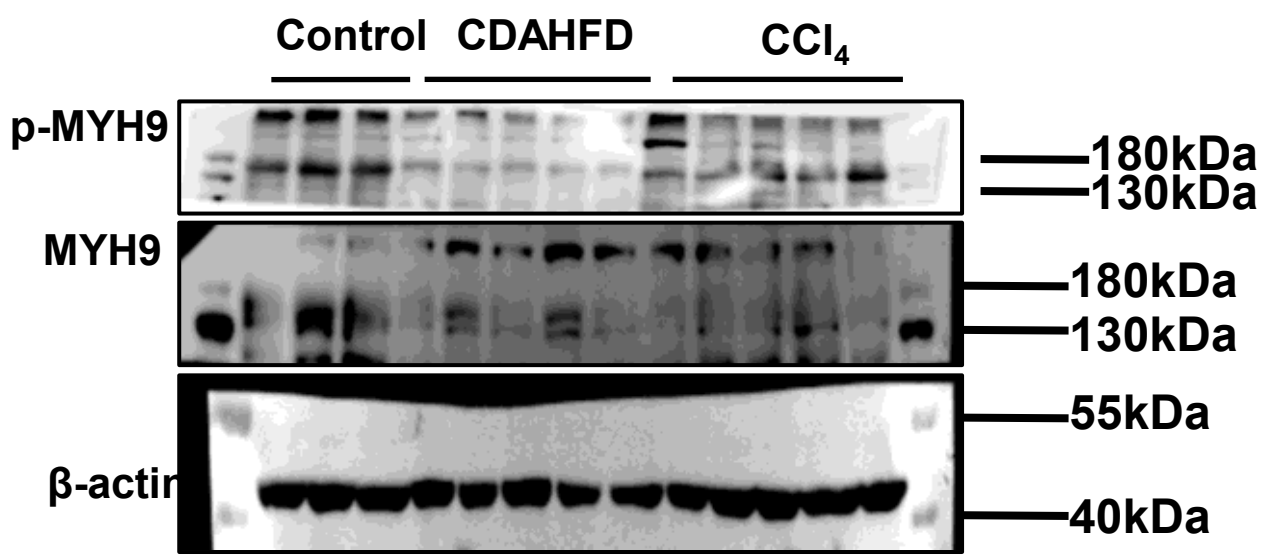

Figure S12C

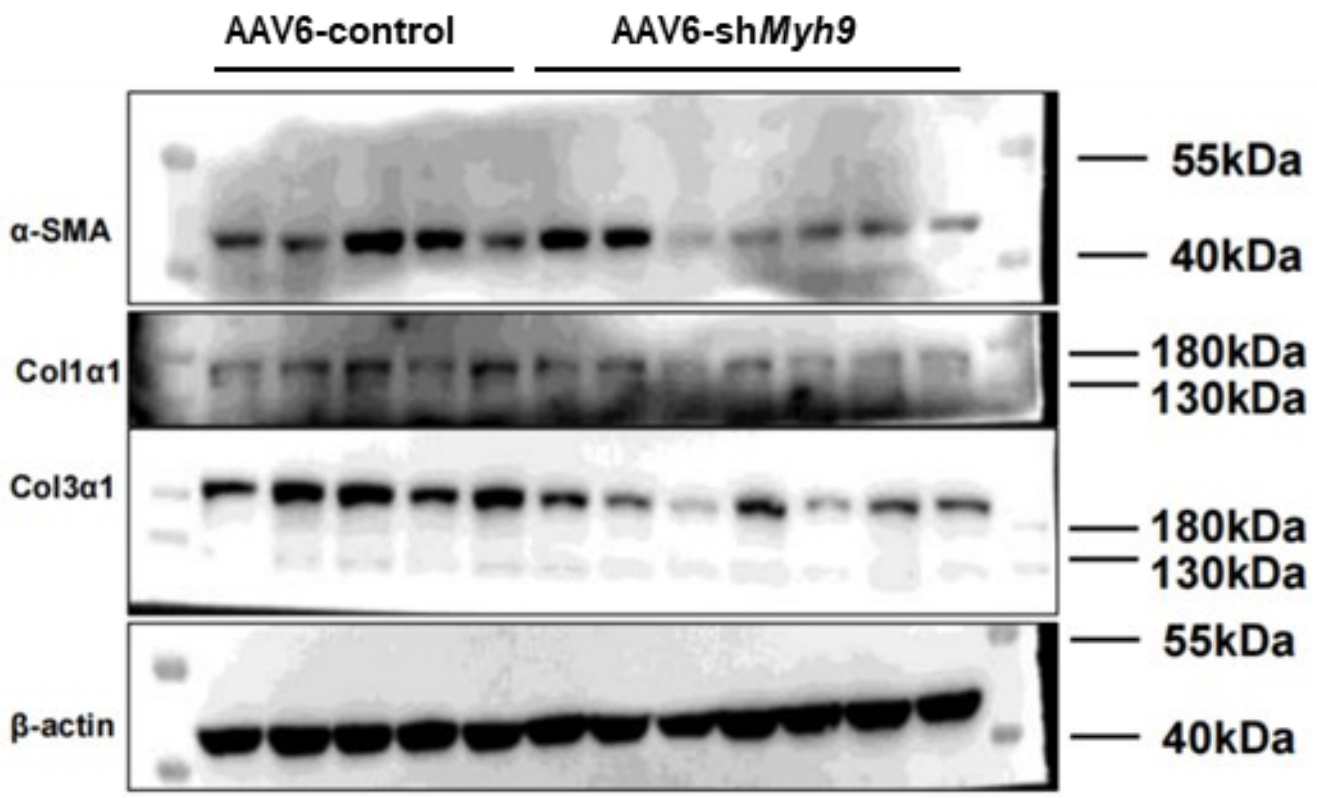

Figure S13E

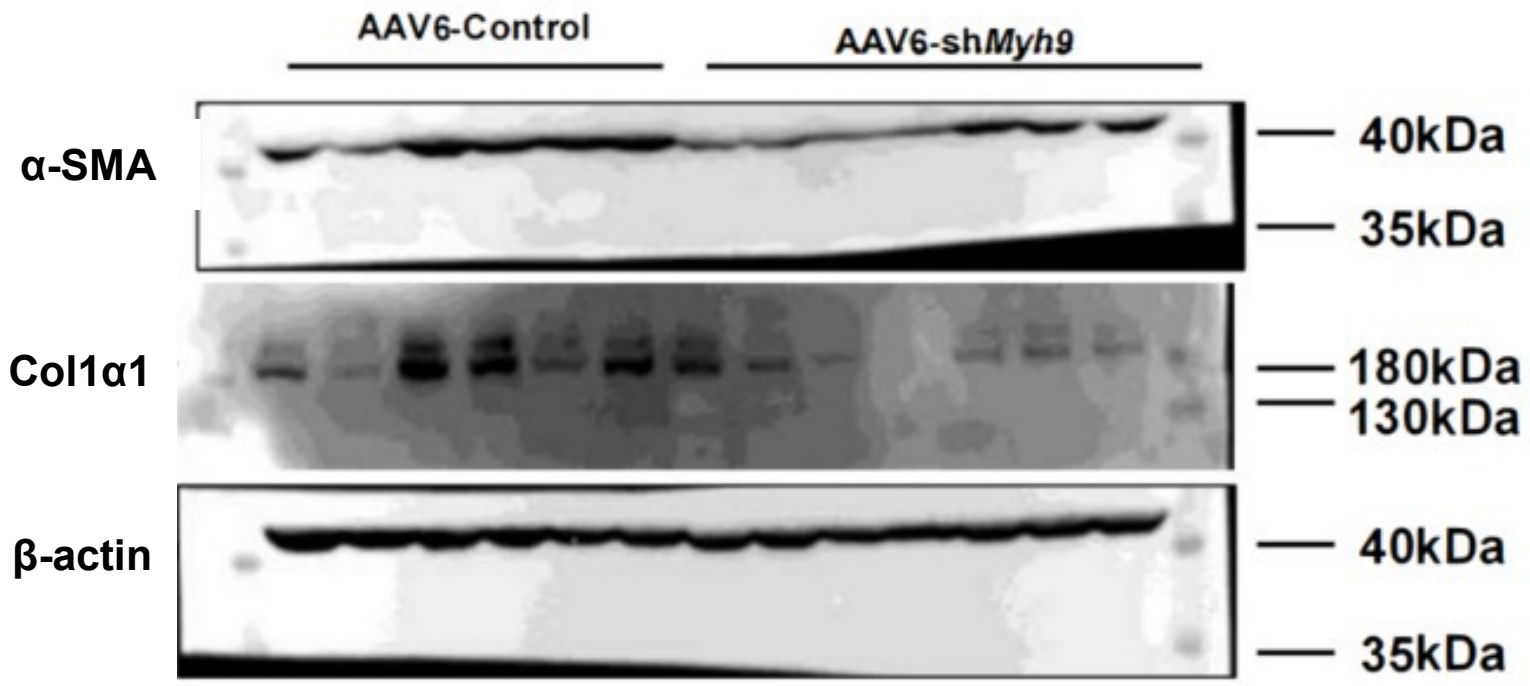

Supplement: Supplementary file 2 [file jci-136-203412-s056.pdf]
